# Disapproval aversion or inflated inequity acceptance? The impact of expressing emotions in ultimatum bargaining

**DOI:** 10.1007/s10683-017-9554-z

**Published:** 2017-11-13

**Authors:** Josie I. Chen, Kenju Kamei

**Affiliations:** 1 Department of Economics, National Taipei University, No.151, Daxue Rd., Sanxia Dist., New Taipei City, 23741 Taiwan, ROC; 2 Department of Economics and Finance, Durham University, Mill Hill Lane, Durham, DH1 3LB UK

**Keywords:** Experiment, Ultimatum game, Emotion, Rating, Disapproval aversion, C91, D03, D82, M21

## Abstract

Past experimental research has shown that when rating systems are available, buyers are more generous in accepting unfair offers in ultimatum bargaining. However, it also suggests that, under these conditions, sellers behave more fairly to avoid receiving negative feedback. This paper experimentally investigates which effect is stronger with the use of a rating system: buyers’ inflated inequity acceptance or sellers’ disapproval aversion. We explore this question by varying the information condition on the buyers’ side. Our experiment shows that in a setup where the size of the pie is common knowledge for both buyers and sellers, when a rating system is present, the sellers exhibit disapproval aversion but the buyers do not display greater acceptance of inequity. By contrast, when only sellers are aware of the size of the pie, sellers behave aggressively to exploit buyers and their behavior does not change in the presence of a rating system; however, buyers display greater acceptance of inequity when a rating system is present. We discuss how these results can be explained by a theoretical model that includes sellers’ social disapproval aversion and buyers’ disappointment aversion in addition to the players’ inequality aversion.

**Electronic supplementary material:**

The online version of this article (10.1007/s10683-017-9554-z) contains supplementary material, which is available to authorized users.

## Introduction

In recent decades, economists have devoted considerable efforts to studying the impact of expressing emotions on people’s behavior when there is complete information and have found that emotional expression may affect both the senders and the recipients of the expression. On the one hand, it has been documented that people have preferences against receiving disapproval from others. Consequently, they behave pro-socially to ensure that they do not receive negative feedback.^1^ On the other hand, expressing emotions has also been known to affect the behavior of the senders of those emotions. For example, in a one-shot ultimatum game, buyers (responders) are more likely to accept unfair offers when given opportunities to express emotions (e.g., Xiao and Houser 2005; Güth and Levati 2007). This finding suggests that expressing negative emotions is a substitute for punishing matched sellers (proposers), which thus increases buyers’ *inequity acceptance*. However, which effect is more dominant when there is a rating system present in ultimatum bargaining: buyers’ inflated inequity acceptance or sellers’ disapproval aversion? Does the relative strength of these two effects differ by information condition? This paper is the first to study how the impact of expressing emotions differs according to whether the size of the pie is common knowledge to all players (complete information) or is only known to sellers (incomplete information).

Although past studies have used complete information setups to study the effects of expressing emotions, understanding such effects under the incomplete information setting is equally important for two particular reasons. First, the incomplete information setup is more realistic under some circumstances, in which sellers are better informed than buyers about the products they sell. On the one hand, such price settings as foods in grocery stores and standard items such as pens, university textbooks and music CDs in physical stores or on the online marketplace (e.g., Amazon.com) can be described as buyers having complete information. On the other hand, some transactions can be best described by containing incomplete information on the buyers’ side. Examples include used products, medical services, and education services such as higher education. Users usually become aware of the quality of the goods and services only after they have purchased or consumed them. A rating system is available in some cases (e.g., standard items or used products on the online marketplace, lectures at universities), but not for other cases (e.g., goods in grocery stores, used items in classified ads such as Craigslist).

Second, asymmetry of information between sellers and buyers is known to change the picture of the bargaining between them. Many experiments with complete information have demonstrated that people prefer fair outcomes in ultimatum games (for a survey, see Roth 1995). At the same time, however, past studies have shown that in incomplete information setups (a) sellers can become greedier and their offering prices can be close to what standard theory predicts and (b) buyers are more likely to accept unfair offers.^2^ These results may extend to an environment with a rating system. Moreover, the presence of a rating system may make buyers open to even more unfair offers with incomplete information. Buyers behave conservatively to avoid the disappointment that they may experience when the realized size of the pie is lower than their expectation. However, with a rating system present, buyers can release such negative emotions by using ratings; as such, buyers do not need to lower their acceptance level of inequity due to disappointment.

Our experiment is based on a finitely repeated ultimatum game. We design four treatments by varying two dimensions. The first dimension is constituted by whether buyers are given the opportunity to rate sellers or not. Sellers are informed of their own ratings after the transactions have been completed. The ratings are not disclosed to other group members and are not carried over from period to period.^3^ The second dimension is constituted by whether buyers are informed of the size of the pie or not (i.e., complete versus incomplete information). In each treatment, subjects are randomly assigned the role of either seller or buyer. Seller *j* has one commodity, the value of which is randomly drawn from integers between 0 and 40, and is then randomly matched with a buyer *i*. Seller *j* submits an offering price (*p*_*sj*_) and buyer *i* submits a purchase threshold (*p*_*bi*_). If *p*_*sj*_ ≤ *p*_*bi*_, then the transaction between *i* and *j* is closed.

We first theoretically describe how bargaining between seller *j* and buyer *i* could result in more unequal divisions under incomplete information than under complete information conditions. We then describe how a fairer or a less fair situation could hold as an equilibrium outcome with a rating system if players are inequality averse and sellers exhibit disapproval aversion. We then show that with a rating system present, buyers’ inflated inequity acceptance could dominate sellers’ disapproval aversion when the buyers are not aware of the size of the pie (incomplete information), because buyers dislike disappointment resulting from possibly a lower-than-expected size of the pie.

Our experiment results largely confirm the theoretical analyses regarding social disapproval aversion and disappointment aversion. First, the divisions of the pies were much more unequal with incomplete than with complete information. Second, sellers exhibited disapproval aversion with complete information, which is consistent with past research. Specifically, the sellers attempted to keep smaller shares of the pies when the rating system was available, compared with when it was not available. Third, and in sharp contrast, with incomplete information, sellers’ disapproval aversion did not affect their bargaining behavior. Whether or not the rating system was present, sellers aggressively attempted to take more from their buyers. Instead, buyers displayed greater acceptance of inequity when the rating system was present than otherwise. The enhanced buyers’ acceptance of unfair offers increased the inequality in the divisions of the pies. In short, our paper suggests that a rating system may have opposite effects depending on the information conditions.

The rest of the paper proceeds as follows: Sect. 2 describes our experimental design. Section 3 provides theoretical considerations. Section 4 reports the experiment results, while Sect. 5 concludes.

## Experimental design

Our experiment is based on a finitely repeated ultimatum game. At the onset of the experiment, subjects are randomly assigned to an interaction unit (group) with another nine subjects. A group of ten subjects is then randomly divided into two subgroups of five subjects. Five subjects in one subgroup are assigned the role of seller (proposer) and the five in the other subgroup are assigned the role of buyer (responder).^4^ The initially assigned roles do not change throughout the entire experiment. Subjects do not interact with subjects in other groups. The number of periods is 50 and there is no break between periods.

The structure of each period is identical. At the onset of a period *t*, each seller is randomly matched with a buyer in his group. Since there are five buyers and five sellers in a group, the probability that a seller is matched with the same buyer both in period *t* and period *t* − 1 is 20%. In each period, every seller has one commodity whose quality is the same across all five sellers in the group. The quality (true value) of the commodity, *q*_*t*_, is randomly (i.e., with a probability of 1/41) drawn from the set of integers ranging between 0 and 40 in each period. The random drawing process is independent across periods. The experimental design follows the standard ultimatum games with a strategy method. Each seller proposes a price, *p*_*sj*_, to sell a commodity to his matched buyer. They can sell at most one commodity. *p*_*sj*_ must be an integer ranging from 0 to 40. Each buyer simultaneously submits a purchase threshold, *p*_*bi*_, to her matched seller.^5^ If *p*_*sj*_ ≤ *p*_*bi*_, the deal between buyer *i* and seller *j* is closed; seller *j* obtains a payoff of *p*_*sj*_ − *q*_*t*_/2, and buyer *i* obtains a payoff of *q*_*t*_ − *p*_*sj*_. Here, we can interpret *q*_*t*_/2 as the production cost of a commodity or the value of it for the seller. If *p*_*sj*_ > *p*_*bi*_, the deal is not closed, and the payoffs for both players are zero in that period. Note that when a deal is closed but *q*_*t*_ − *p*_*sj*_ < 0, the buyer incurs a loss. Each player is informed of their own interaction outcome at the end of each period. Buyer *i* is then made aware of the seller’s offering price. However, seller *j* is not informed of the matched buyer’s purchase threshold; the seller is only informed of whether the offer was accepted. Subjects are paid based on the sum of their payoffs earned during all 50 periods.^6^ The number of periods, the assignment procedure of the roles, the distribution of *q*_*t*_, and the interaction rules, such as the formula for the payoffs, are common knowledge to the subjects.

We design four treatments by varying two dimensions in the experiment. The first dimension is whether the value of the commodity (*q*_*t*_) is known to both sellers and buyers, or is only known to sellers, before the transactions in each period. In the incomplete information condition, the buyers learn the realized value of *q*_*t*_ at the end of each period.^7^ The second dimension is whether there is a rating system available to buyers or not. The four treatments are referred to as the “No Rating, Complete Information” (N–C) treatment, the “Rating, Complete Information” (R–C) treatment, the “No Rating, Incomplete Information” (N–IC) treatment, and the “Rating, Incomplete Information” (R–IC) treatment.

In the R–C and R–IC treatments, each buyer is given an opportunity to rate their matched seller on a 10-point scale in every period after learning their transaction outcome (including their own and their matched seller’s payoffs). Buyers are instructed that the lowest number (0) means “very unfair,” 3 means “unfair,” 7 means “fair,” and the highest number (10) means “very fair.”

At the end of each treatment, demographic information, such as gender, is collected. These responses are used as control variables in the data analysis.

## Theoretical consideration and hypotheses

Each seller is randomly assigned an identification number, is randomly paired with a buyer, and then these interact with each other in each period. As discussed in Sect. 2, the number of interactions is finite and is common knowledge to both the buyers and the sellers. The standard theory therefore predicts the same behavior of the subjects in each stage game. The standard theory predictions are the same for the N–C and R–C treatments, and also for the N–IC and R–IC treatments, because the rating opportunities held by buyers do not affect the (material) payoffs for the buyers and sellers.

Our experiment uses the standard ultimatum game with a strategy method. Thus, there are multiple equilibrium outcomes in each treatment. First, in the N–C and R–C treatments, for a given *q*_*t*_, any division of the pie (*p* − *q*_*t*_/2)/(*q*_*t*_/2) × 100[%] for the seller and (*q*_*t*_ − *p*)/(*q*/2) × 100[%] for the buyer, where *p* ∈ [*q*_*t*_/2, *q*_*t*_], can be realized as an equilibrium outcome. Note that the size of the pie in this experiment is *q*_*t*_/2 (= *q*_*t*_ − *q*_*t*_/2). Under a Nash equilibrium, the same *p* is offered by a seller and is also set as a purchase threshold by the buyer, and their transaction is closed. In addition, there are many equilibrium outcomes where deals are not closed, and both sellers and buyers receive nothing (see Appendix A.1).

Second, in the N–IC and R–IC treatments, while a seller can condition his strategy on *q*_*t*_, the matched buyer selects a purchase threshold *p*_*b*_ irrespective of *q*_*t*_, as the buyer is not informed of the value of *q*_*t*_. We write *p*_*sj*_: [0, 40] → [0, 40] as the strategy of seller *j*, and *p*_*bi*_ ∈ [0, 40] as the strategy of buyer *i*.^8^ As shown in Appendix A.3, we find two kinds of Bayesian Nash equilibria (BNE). In the first kind of equilibrium, the seller has a clear advantage: the buyer obtains an expected payoff of 0 and only the seller obtains a positive payoff.^9^ In other words, only extremely unequal divisions of the pies are realized as equilibrium outcomes. In the second class of BNE, the transaction is not executed. The following is an example: the seller always posts a price so that *p*_*s*_ > 20, and the buyer sets her purchase threshold at 0.

### **Summary 1**

*Equilibrium Analysis Based on the Standard Theory.*

*There are multiple equilibria in all of the four treatments. In an equilibrium where a deal is closed, both the buyer and seller can obtain positive shares of the pie in the N*–*C and R*–*C treatments. However, in the N*–*IC and R*–*IC treatments, only a very unequal division of the pie is observed in a Bayesian Nash equilibrium where a deal is closed.*

Based on Summary 1, we now have the following testable hypothesis for the impact of the information condition on subjects’ divisions of the pies.

### **Hypothesis 1**

A more unequal division of the pie is realized in the N–IC and R–IC treatments than in the N–C and R–C treatments.

*Players’ Inequality Aversion and Sellers’ Disapproval Aversion*

Unlike as given in Summary 1, buyers obtain some positive payoffs and thus the divisions of the pies can be less unequal in equilibrium even in the incomplete information treatments, if we assume that people have other-regarding preferences. As an illustration, assume that all subjects have inequality-averse preferences (e.g., Fehr and Schmidt 1999) and that it is common knowledge. The utility function of the inequality-averse buyer can be expressed as:

1 $$u_{bi} (\pi_{bi} ,\, \pi_{sj} ) = \pi_{bi} - \mu_{i} \, \cdot f(\pi_{bi} - \pi_{sj} )$$

where *µ*_*i*_ indicates the utility weight of the inequality of buyer *i* and it differs by buyer. In our theoretical analysis, we use the following quadratic function as *f*(.)^10^:

2 $$f\left( {\pi_{bi} - \pi_{sj} } \right) = \left( {\pi_{bi} - \pi_{sj} } \right)^{2} .$$

The utility function of inequality-averse seller *j* can be defined likewise:

3 $$u_{sj} (\pi_{sj} , \pi_{bi} ) \, = \pi_{sj} - \mu_{j} \cdot f(\pi_{sj} - \pi_{bi} )$$

As shown in Appendix A.2, regardless of the information condition, the seller’s best response strategy (price) would depend on *q*_*t*_ and *µ*_*j*_, and in equilibrium where a deal is closed, both the seller and the buyer always obtain positive (expected) payoffs in all the treatments.^11^ As a result, for a given *q*_*t*_, the degree of inequality with regards to the division of the pies is mitigated to some degree.^12^

Next, we consider how the presence of a rating system may affect the players’ behaviors using the model with inequality aversion [Eqs. (1)–(3)]. As in past research, let us assume that a seller incurs a psychological loss when he receives a negative rating from the buyer; however, the seller receives a psychological gain when the rating is positive. With this assumption, the direction of the effects of the rating system does not differ by the information condition. We use the R–C treatment to illustrate possible consequences of expressing emotions, incorporating the framework used by Cooper and Lightle (2011, 2012) into our setup. For simplicity, we also assume that the psychological loss or gain of a seller is proportional to the difference from the neutral rating, 5, and is expressed as *c*·(*r* − 5), where *c* is a positive constant and *r* is the rating (∈ {0, 1, …, 9,10}). With this setup, the seller’s payoff is re-written by $\pi_{sj}^{{\prime }}$:

4 $$\pi_{sj}^{{\prime }} = \pi_{sj} + c \cdot \left( {r {-} 5} \right) .$$

We also assume that there are no costs on the buyer’s side because giving ratings is mandatory.^13^ In this framework, seller *j* selects *p*_*sj*_ to maximize his utility $u_{sj} (\pi_{sj}^{{\prime }}, \pi_{bi} )$. Buyer *i* selects *p*_*bi*_ and then *r* in the later rating stage to maximize her utility $u_{bi} (\pi_{bi} , \pi_{sj}^{{\prime }} )$. As detailed in Appendix A.5, the buyer would utilize the rating opportunity in order to shrink her disutility from inequality $(\text{i.e.},\mu_{i} \cdot f(\pi_{bi} - \pi_{sj}^{{\prime }} )$. This implies that when their transaction is closed (*p*_*bi*_ ≥ *p*_*sj*_), the buyer’s rating scores are negatively correlated with the seller’s offering prices (equivalently, the seller’s payoff).^14^ This analysis is summarized as Hypothesis 2 below:

### **Hypothesis 2**

Buyers give positive (negative) ratings to sellers who take less (more) from the pies when their transactions are closed.

As the disutility the buyer incurs from material inequality is diminished by acts of expressing emotions, the buyer shows more willingness to accept a higher price (i.e., an unfair division of the pie), compared with when the rating system is not available. Note that the buyer would accept an offer by matching *p*_*bi*_ with the seller’s offering price whenever $u_{bi} (\pi_{bi} ,\, \pi_{sj}^{{\prime }} ) \, \ge \, 0$ in equilibrium. However, as explained in Appendix A.5, there is not only a fairer equilibrium but also a less fair equilibrium with rating than without rating, due to psychological costs or gains associated with receiving negative or positive feedback. On the one hand, the disapproval-averse seller would attempt to keep less by setting a lower price in the R–C (R–IC) than in the N–C (N–IC) treatment when by doing so the seller expects to avoid incurring a psychological cost from receiving negative feedback. On the other hand, the seller would conversely attempt to keep more in the R–C (R–IC) than in the N–C (N–IC) treatment when he expects to receive negative feedback. The former (latter) situation leads to an equilibrium in which fairer (less fair) divisions of the pies are realized, compared with a situation without a rating possibility.

### **Summary 2**

*Equilibrium Analysis Based on Inequality Aversion and Disapproval Aversion.*

*(a) Given q, buyers exhibit more willingness to accept unfair offers in the R*–*C (R*–*IC) treatment than in the N*–*C (N*–*IC) treatment. However, (b) there exists not only a fairer equilibrium but also a less fair equilibrium in the R*–*C (R*–*IC) treatment than in the N*–*C (N*–*IC) treatment.*

*Buyers’ Disappointment Aversion*

Under which information condition could buyers exhibit stronger inequity acceptance: complete or incomplete information? We now explain that stronger inequity acceptance may be observed in the incomplete information setting due to buyers’ disappointment aversion. A large body of literature suggests that a subject could incur a disutility from disappointment if realized outcomes are lower than his/her certainty equivalent in risky decisions (see Gul 1991; Routledge and Zin 2010 for theoretical models). If buyers exhibit disappointment aversion in our context, their purchase thresholds would differ between the R–IC and N–IC treatments, even without disapproval aversion (see Appendix Section A.6 for an illustrative analysis in a simple setting). This results from two forces: (a) disappointment-averse buyers in the N–IC treatment submit low purchase thresholds to avoid disappointment from a possibly lower *q*; but (b) disappointment aversion would not affect buyers’ behaviors in the R–IC treatment because of the rating opportunity.^15^ In contrast to the incomplete information setup, buyers do not experience such disappointment in the R–C and N–C treatments as they are aware of *q*_*t*_ when making decisions. The likely impact of buyers’ disappointment aversion with incomplete information suggests that the difference between buyers’ inequity acceptance with and without rating could be greater in the incomplete information setup than in the complete information setup.

### **Summary 3**

*Analysis Based on Inequality Aversion and Buyers’ Disappointment Aversion.*

*A stronger degree of inflated inequity acceptance is observed in the incomplete information setup than in the complete information setup.*

We explained that the theoretical analyses do not provide a point prediction (Summary 2(b)). One may wonder to which equilibrium subjects’ interactions could converge through adjustments to their strategy over the course of repetition.^16^ We could provide a hypothesis to this question using past research findings and Summary 3. On the one hand, as mentioned in Sect. 1, people are known to preferably avoid receiving disapproval from others and therefore behave more fairly when a rating system is available in complete information settings (e.g., Masclet et al. 2003; Dugar 2013; Ellingsen and Johannesson 2008; López-Pérez and Vorsatz 2010; Xiao and Houser 2009). This suggests that subjects’ interactions could converge towards a fairer equilibrium in the R–C treatment. In such an equilibrium, sellers’ disapproval aversion can dominate buyers’ inflated inequity acceptance. On the other hand, a less fair equilibrium may instead be realized with incomplete information, because of Summary 3. Due to disappointment-averse motives, buyers could exhibit lower acceptance of inequity in the N–IC than in the N–C treatment. However, due to the presence of the rating system, buyers in the R–IC treatment do not need to care about disutility from disappointment and might accordingly become more vulnerable to their sellers’ exploitable behaviors. Reflecting buyers’ vulnerability, their interactions could converge towards a less fair equilibrium where sellers no longer exhibit disapproval aversion in the R–IC treatment. These considerations provide our third hypothesis:

### **Hypothesis 3**

(a) Sellers exhibit strong disapproval aversion in the R–C treatment. (b) By contrast, in the R–IC treatment, buyers exhibit strong inequity acceptance.

## Results

Four sessions per treatment—two at Brown University and two at the National Taiwan University—were conducted. A total of 320 students participated in the experiment. All instructions were neutrally framed (see the Appendix for the instructions).^17^ The experiment was programmed and conducted using the z-Tree software (Fischbacher 2007). All participants were students at either Brown University or National Taiwan University.^18^ No subjects participated in more than one session. Each session contained two groups, which consisted of ten subjects each. The value of the commodity (*q*_*t*_) was randomly selected in each period, and the same value was used for *q*_*t*_ for both groups.^19^ The sessions lasted one hour on average. No communication between the subjects was permitted during the sessions. Subjects had to answer several control questions to ensure their understanding of the experiment before the sessions began.

Section 4 is devoted to an analysis of the subjects’ behaviors, linking to the hypotheses formulated in Sect. 3. We first go over the descriptive statistics in Sect. 4.1. We next report buyers’ rating behaviors in Sect. 4.2. Lastly, we study the impact of each treatment factor while also considering the panel structure of the data in Sect. 4.3.

### Bargaining between sellers and buyers and their interaction outcomes

Table 1 shows the key results by treatment separately for the USA and Taiwan sessions. In this table, in order to study subjects’ bargaining behaviors, we calculated the amounts that a seller attempted to keep, which we call the “keep” of the seller in the paper, and the share of it out of the size of the pie, which we call the “keep share” of the seller. As the size of the pie in this experiment is *q*_*t*_/2 (= *q*_*t*_ − *q*_*t*_/2), the seller *j*’s keep value and keep share are each calculated as ($p_{sj}^{t} - q_{t} /2$) and $(p_{sj}^{t} - q_{t} /2 )/\left( {q_{t} /2} \right)$, respectively. Likewise, we calculated the payoff for a buyer based on the lowest acceptable offer specified by the buyer, i.e., (*q*_*t*_ − *p*_*bi*_^*t*^); which we call the “keep” of the buyer. We also define buyer *i*’s “keep share” as (*q*_*t*_ − *p*_*bi*_^*t*^)/(*q*_*t*_/2).

**Table 1:** Summary of results

Treatment name	Rating	Information condition	Number of subjects (number of groups)	Avg. value of commodity: $\overline{q}$ ^#2^	Average keep and average keep share				Bargaining outcomes	
					Sellers’ keep: $\overline{{p_{s} - \frac{q}{2}}}$	Sellers’ keep share: $\overline{{\left( {p_{s} - \frac{q}{2}} \right)/\left( {\frac{q}{2}} \right)}}$	Buyers’ keep: $\overline{{q - p_{b} }}$	Buyers’ keep share: $\overline{{\left( {q - p_{b} } \right)/\left( {\frac{q}{2}} \right)}}$	Avg. shares of sellers in closed deals^#1^ (%)	Average acceptance rate (%)
			(1)	(2)	(3)	(4)	(5)	(6)	(7)	(8)
*(I) USA sessions*										
N–C (No Rating, Complete Information)	No	*q*_*t*_ is known to both sellers and buyers	40 (4)	18.57 [12.80]	5.30 [4.22]	0.56 [0.25]	2.56 [3.54]	0.23 [2.05]	51.61	81.60
R–C (Rating, Complete Information)	Yes	*q*_*t*_ is known to both sellers and buyers	40 (4)	18.54 [12.23]	4.85 [4.31]	0.49 [0.23]	2.56 [4.85]	0.25 [1.12]	45.52	81.70
N–IC (No Rating, Incomplete Information)	No	*q*_*t*_ is known only to sellers	40 (4)	20.94 [11.64]	8.03 [5.15]	1.53 [2.26]	2.18 [13.45]	− 1.03 [3.72]	176.68	60.60
R–IC (Rating, Incomplete Information)	Yes	*q*_*t*_ is known only to sellers	40 (4)	22.78 [11.86]	9.04 [5.19]	2.04 [5.40]	0.23 [14.60]	− 1.88 [7.55]	243.88	64.00
All data	–	–	160 (16)	20.21 [12.27]	6.80 [5.06]	1.16 [3.00]	1.88 [10.41]	− 0.61 [4.45]	118.35	71.98

Treatment name	Rating	Information condition	Number of subjects (Number of groups)	Avg. value of commodity: $\overline{q}$ ^#2^	Average keep and average keep share				Bargaining outcomes	
					Sellers’ keep: $\overline{{p_{s} - \frac{q}{2}}}$	Sellers’ keep share: $\overline{{\left( {p_{s} - \frac{q}{2}} \right)/\left( {\frac{q}{2}} \right)}}$	Buyers’ keep: $\overline{{q - p_{b} }}$	Buyers’ keep share: $\overline{{\left( {q - p_{b} } \right)/\left( {\frac{q}{2}} \right)}}$	Avg. shares of sellers in closed deals^#1^	Average acceptance rate (%)
			(9)	(10)	(11)	(12)	(13)	(14)	(15)	(16)
*(II) Taiwan sessions*										
N–C (No Rating, Complete Information)	No	*q*_*t*_ is known to both sellers and buyers	40 (4)	21.50 [12.25]	6.35 [4.65]	0.73 [2.66]	3.44 [3.41]	0.35 [0.32]	51.50%	73.20
R–C (Rating, Complete Information)	Yes	*q*_*t*_ is known to both sellers and buyers	40 (4)	19.58 [11.84]	5.76 [4.59]	0.54 [0.18]	3.54 [2.83]	0.39 [0.22]	49.74%	78.00
N–IC (No Rating, Incomplete Information)	No	*q*_*t*_ is known only to sellers	40 (4)	22.51 [11.28]	8.14 [4.69]	2.06 [5.14]	3.05 [13.00]	− 1.53 [6.55]	238.42%	60.60
R–IC (Rating, Incomplete Information)	Yes	*q*_*t*_ is known only to sellers	40 (4)	19.92 [11.97]	8.26 [4.72]	2.14 [4.50]	− 0.41 [14.28]	− 2.11 [7.11]	240.40%	65.10
All data	–	–	160 (16)	20.88 [11.90]	7.13 [4.79]	1.36 [3.74]	2.41 [10.04]	− 0.72 [4.94]	134.69%	69.23

The numbers in squared bracket are standard errors. #1 The average share of buyers in a given treatment is 100% minus the value in this column. #2 The average size of the pie is  $\overline{q}/2$

Among others, four clear findings, each of which holds both for the USA and Taiwan sessions, were obtained. First, consistent with Hypothesis 1, the divisions of the pies drastically differed according to the information condition (see columns (7) and (15)). The average realized shares of sellers for closed deals were around 45% to 52% in the N–C and R–C treatments. However, sellers became more selfish with incomplete information. Unlike the N–C and R–C treatments, the average keep shares of sellers were significantly higher than 1 in the N–IC and R–IC treatments (columns (4) and (12)).^20^^,^ 21 As a result, the average realized shares of sellers were much higher than 100% in the N–IC and R–IC treatments (columns (7) and (15)).^22^ Due to the sellers’ aggressive behaviors, buyers’ average acceptance rates were significantly lower with incomplete than with complete information for each comparison (columns (8) and (16)).^23^^,^ 24

#### *Result 1*

Hypothesis 1 holds. This can be explained by sellers’ attempts to take more from buyers with incomplete information. Due to the sellers’ aggressive behavior, buyers’ acceptance rates were lower with incomplete information than with complete information.

Second, consistent with Hypothesis 3(a), the average sellers’ keep and keep share were both lower in the R–C than in the N–C treatment (columns (3), (4), (11), and (12)).^25^ This suggests that sellers exhibited disapproval aversion in the R–C treatment. However, the buyers’ bargaining behaviors are almost at the same levels between the R–C and N–C treatments (columns (5), (6), (13), and (14)). Third, in clear contrast, the average sellers’ keep and keep share were both *larger* with rating than without rating in the incomplete information setting. This suggests that sellers did not exhibit disapproval aversion in the R–IC treatment. However, consistent with Hypothesis 3(b), buyers’ behaviors were significantly affected by the presence of the rating system. Both buyers’ keep and keep shares were far lower in the R–IC than in the N–IC treatment (see again columns (5), (6), (13), and (14)).^26^

#### *Result 2*

(i) With complete information, consistent with Hypothesis 3(a), sellers’ keep values and keep shares were both lower in the R–C than in the N–C treatment. (ii) With incomplete information, consistent with Hypothesis 3(b), buyers’ keep values and keep shares were both lower in the R–IC than in the N–IC treatment.

Fourth, the impact of the rating system on subjects’ bargaining outcomes differs according to the information condition (columns (7) and (15)), driven by Result 2. With complete information, the presence of the rating system reduced the realized shares of sellers from the closed trades. With incomplete information, by contrast, it instead *increased* realized the shares of sellers and, accordingly, the division of the pies became more unequal between sellers and buyers.

### Buyers’ rating behaviors

As explained in Sect. 4.1, subjects’ behaviors were consistent with Hypothesis 3. However, whether sellers are disapproval averse or not, buyers would become more tolerant to unfair offers with a rating system than they are without a rating system if buyers dislike disappointment from a possibly lower *q* (see Sect. 3). To what extent do our data fit the theoretical implications obtained based on disapproval aversion? To address this question, we will test Hypothesis 2.

We take a regression approach in which the dependent variable is a rating score given by buyer *i* to seller *j* (Table 2). In this regression, either the matched seller *j*’s keep (columns (1) and (2)) or keep share (columns (3) and (4)) is included as an independent variable.^27^ First, the estimation shows that when their transactions are closed, these two independent variables are both negative predictors for the rating scores sellers receive from buyers, both in the R–C and the R–IC treatments. This is consistent with Hypothesis 2 and in line with findings from past research (e.g., Xiao and Houser 2005; Ellingsen and Johannesson 2008; Lumeau et al. 2015). This suggests that, at least, buyers believe that sellers would dislike receiving disapproval also with incomplete information, and Result 2(ii) may mean that the impact of buyers’ disappointment aversion exceeds that of sellers’ disapproval aversion.

**Table 2:** The determinants of the rating decisions by buyers (Dependent variable: Rating that buyer *i* gave to the matched seller *j* in period *t* ∈ {1, 2, …, 50})

Independent variables	(1)	(2)	(3)	(4)
(a) Seller’s keep in period *t* (i.e., *p*_*sj,t*_ − *q*_*t*_/2)	− 0.575*** (0.063)	− 0.525*** (0.076)	–	–
(b) $\frac{{{\text{Seller's\;keep\;in\;period }}t}}{{q_{t} /2}}$	–	–	− 0.856*** (0.325)	− 0.435 (0.662)
(c) Deal closed dummy {which equals 1 if the trade is closed; 0 otherwise}	1.404*** (0.576)	4.862*** (0.682)	2.697*** (0.526)	2.471*** (0.670)
(d) Complete information dummy {which equals 1 for the NC and R–C treatments; 0 otherwise}	0.935* (0.494)	− 2.658*** (0.781)	1.119** (0.564)	5.179*** (1.011)
(e) Interaction term between variable (a) and variable (c)	–	− 0.448*** (0.060)	–	–
(f) Interaction term between variable (a) and variable (d)	–	0.483*** (0.076)	–	–
(g) Interaction term between variable (b) and variable (c)	–	–	–	− 0.548 (0.769)
(h) Interaction term between variable (b) and variable (d)	–	–	–	− 7.603*** (1.448)
Period Number (= {1, 2, …, 50})	− 0.012** (0.005)	− 0.008 (0.006)	− 0.014*** (0.006)	− 0.012** (0.006)
Constant	7.508*** (1.202)	6.962*** (1.144)	4.209*** (1.109)	4.202*** (1.188)
# of observations	4000	4000	3800	3800
# of left-censored observations	688	688	584	584
# of right-censored observations	856	856	812	812
Log likelihood	− 8232.86	− 7966.17	− 8066.05	− 7919.92
Wald chi^2^	197.35	229.02	55.90	102.87
Prob > chi^2^	< 0.0001	< 0.0001	< 0.0001	< 0.0001
Two-sided *p* value to the null that seller’s keep or keep share is a negative predictor for a seller’s rating score				
*When deals were closed*				
R–C treatment	–	< 0.0001^#1^	–	< 0.0001^#3^
R–IC treatment	–	< 0.0001^#2^	–	0.0448^#4^
*When deals were not closed*				
R–C treatment	–	0.3511^#5^	–	< 0.0001^#6^
R–IC treatment	–	< 0.0001^#7^	–	0.5113^#7^

Individual random-effects tobit regressions with bootstrap standard errors (200 replications). Numbers in parenthesis are standard errors. Control variables include buyers’ demographic variables: a USA dummy (= 1 if sessions were conducted in the USA; 0 otherwise), a female dummy (= 1 if female; 0 otherwise), number of economics courses taken, general political orientation (1 = very conservative to 7 = very liberal) and income of the subject’s family. We omitted the coefficient estimates of these demographic variables to conserve space as these are not related to the hypotheses in the paper. #1 H_0_: variable (a) + variable (e) + variable (f) = 0. #2 H_0_: variable (a) + variable (e) = 0. #3 H_0_: variable (b) + variable (g) + variable (h) = 0. #4 H_0_: variable (b) + variable (g) = 0. #5 H_0_: variable (a) + variable (f) = 0. #6 H_0_: variable (b) + variable (h) = 0. #7 *p* value for the coefficient estimate of variable (a) or (b)

*, **, and *** indicate significance at the 0.10 level, at the 0.05 level and at the 0.01 level, respectively

Second, Table 2 also shows that even when their transactions were not closed, sellers’ keep shares (sellers’ keep values) were significantly negatively correlated with the matched buyers’ ratings in the R–C (R–IC) treatment. This is not consistent with the theoretical analysis discussed based on sellers’ social disapproval aversion in Sect. 3. This may mean that sellers’ intentions to take more, even if unsuccessful, may negatively affect the matched buyers’ welfare and, as such, buyers use the rating opportunities to deal with such psychological disutility.

#### *Result 3*

Whether transactions were closed or not, buyers were more likely to give negative ratings to sellers who attempted to take more from the pies.^28^

### Treatment effects of the information condition and the rating system

We saw that the information condition has a clear impact on sellers’ bargaining behaviors in Sect. 4.1. We also found that the impact of the rating opportunity differs by the information condition, which is consistent with Hypothesis 3. This section is devoted to an analysis of the treatment effects of the information condition and the rating system while controlling for the structure of the panel data. We combined data from both the USA and Taiwan subjects in the analysis, as the general patterns of their behaviors were similar between the two subject groups (Sect. 4.1, Table 1).

**Fig. 1:**
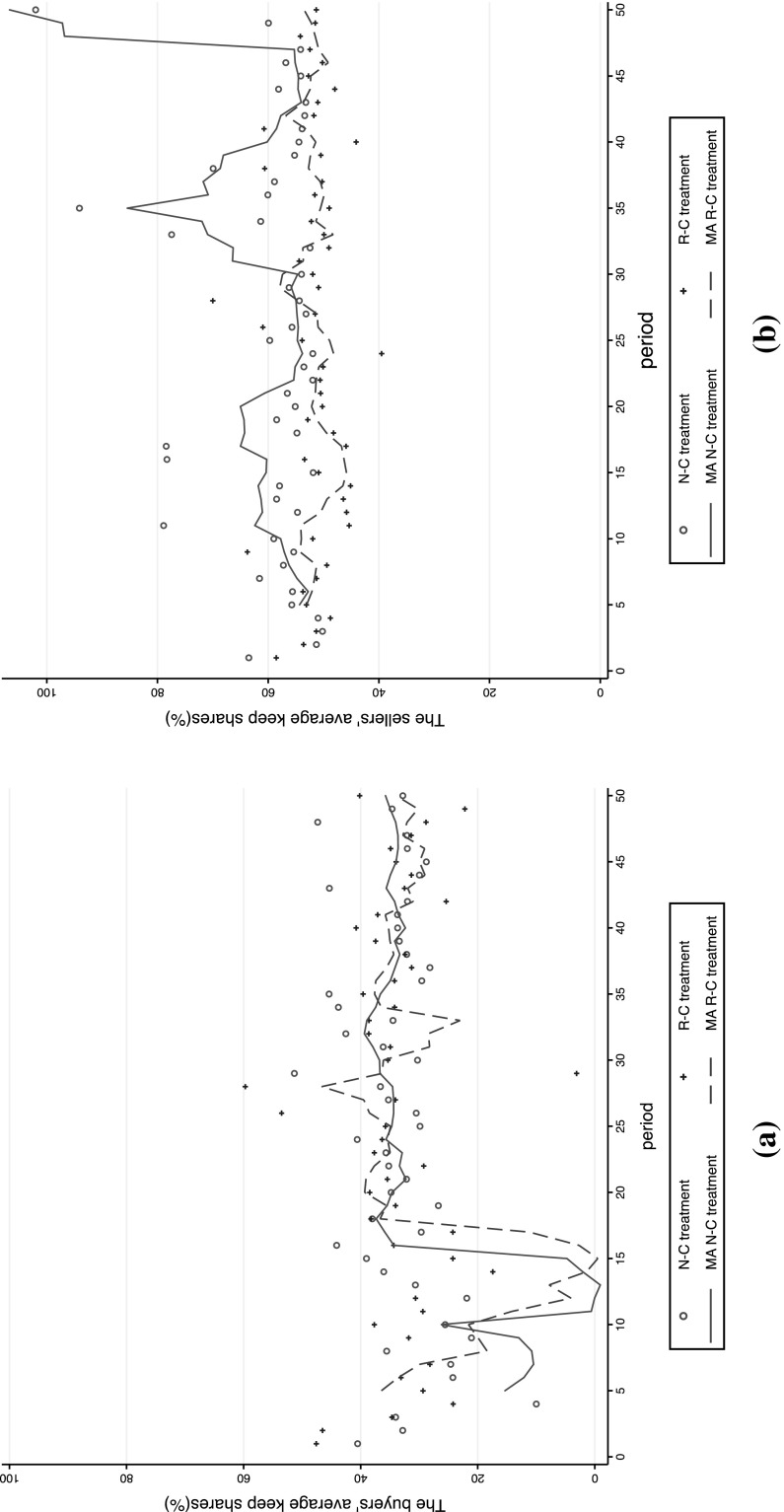
Period-by-period average keep shares of buyers and sellers in the N–C and R–C treatments. **a** Buyers’ average keep shares: (*q* − *p*_*b*_)/(*q*/2). **b** Sellers’ average keep shares: (*p*_*s*_ − *q*/2)/(*q*/2). *Notes*: Two observations in the N–C treatment and two observations in the R–C treatment in figure (**a**), and two observations in the N–C treatment in figure (**b**) are not shown because the values were above 110 or below 0%. The lines of MA indicate simple moving averages of the previous five observations

**Fig. 2:**
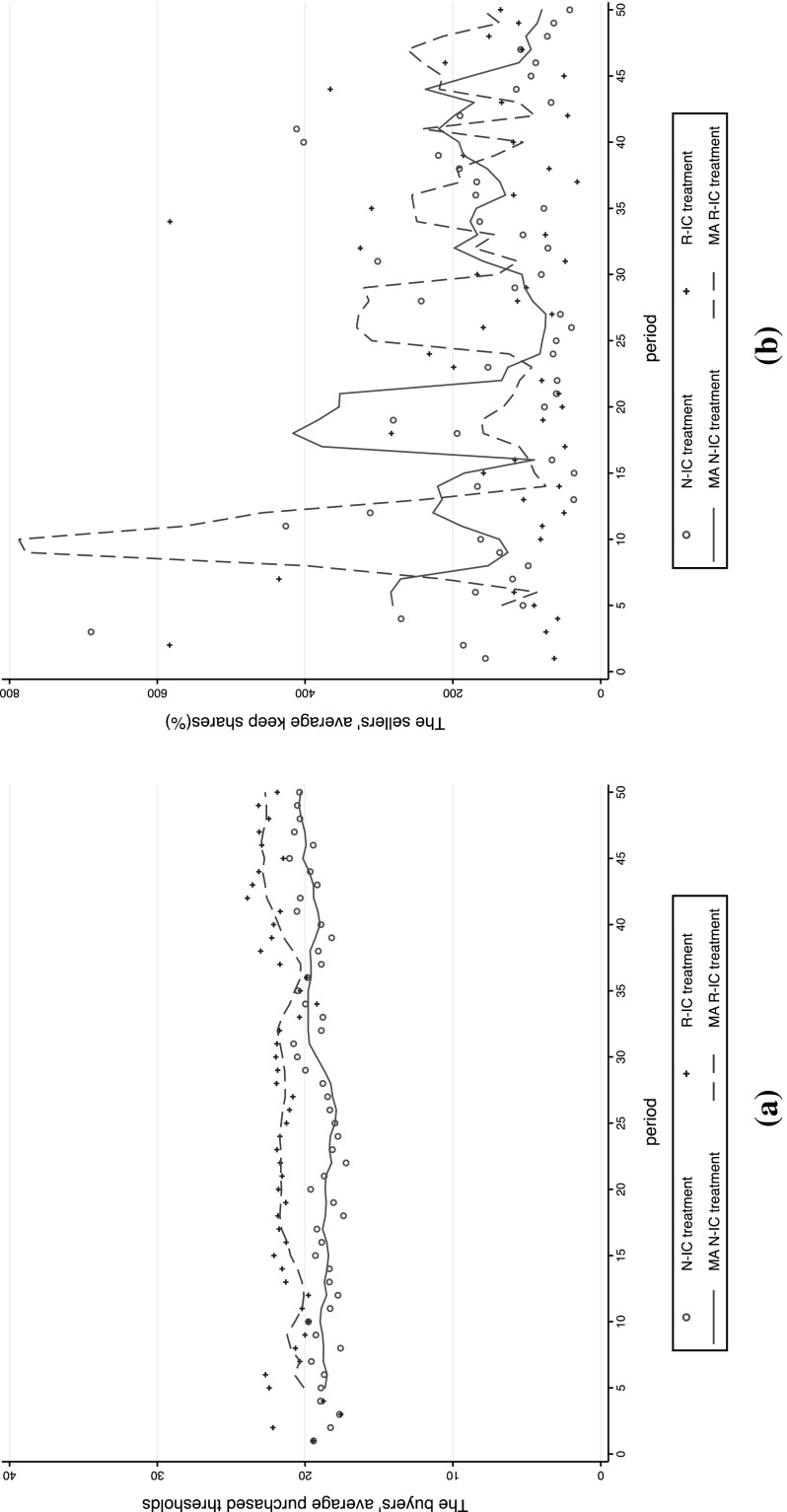
Period-by-period buyers’ average purchase thresholds and sellers’ average keep shares in the N–IC and R–IC treatments. **a** Buyers’ average purchase thresholds: *p*_*b.*_ **b** Sellers’ average keep shares: (*p*_*s*_ − *q*/2)/(*q*/2). *Notes*: One observation in the N–IC treatment and four observations in the R–IC treatment in figure (**b**) are not shown because the values were above 800 or below 0%. The lines of MA indicate simple moving averages of the previous five observations

We first give an overview of the trends of subjects’ bargaining behaviors. Figure 1 reports the trends of buyers’ and sellers’ keep shares in the two complete information treatments. It shows that sellers’ keep shares were lower in the R–C than in the N–C treatment in most periods. In contrast, there were no specific patterns for the trends of buyers’ keep shares. These results resonate with the idea that sellers are disapproval-averse agents and thus consistently attempt to keep smaller shares in the R–C treatment to avoid receiving negative feedback, compared with the N–C treatment. This picture dramatically changed with the incomplete information setups (Fig. 2). In Fig. 2, we drew the trend of *p*_*bi*_, but not buyers’ keep shares, as buyers were not aware of *q* when submitting *p*_*bi*_. First, buyers’ purchase thresholds were consistently higher with rating (the R–IC treatment) than without rating (the N–IC treatment). By contrast, the trends of sellers’ keep shares were on average at very high levels and were similar between the N–IC and R–IC treatments. This resonates with the idea that buyers become more inequity-acceptable when they have an ex-post opportunity to rate as the buyers do not need to care about disappointment due to a possibly lower *q* in the incomplete information setting.

The results we obtained in Sect. 4.1 and the patterns seen at Figs. 1 and 2 are largely confirmed by a formal analysis, where individual random-effects linear regression are used (Table 3).^29^ First, consistent with Result 1, whether the rating system is available or not, sellers attempt to take significantly more from the pies with incomplete than with complete information (variables (b) and (c), Wald test 1 in columns (1) and (2)). Second, the impact of the rating system differs clearly according to the information condition. On the one hand, consistent with Result 2(i), sellers keep less in the complete information setup when the rating system is present than otherwise. This effect is significant at the 10% level (variable (a) in columns (1) and (2)). Parallel to this result, buyers did not display greater acceptance of inequity in the R–C treatment (variable (a) in columns (3) and (4)). On the other hand, with incomplete information, buyers attempt to keep less with rating than without rating, consistent with Result 2(ii). This effect is significant at the 1% level (Wald test 2 in columns (3) and (4)). We thus conclude that what equilibrium outcome is realized with rating may largely depend on the information conditions.

**Table 3:** The effects of each treatment factor on subjects’ bargaining behaviors

Dependent variable	Seller *j*’s keep in period *t* (= (*p*_*sj*_^*t*^ − *q*_*t*_/2))		Buyer *i*’s keep in period *t* (= (*q*_*t*_ − *p*_*bi*_^*t*^))	
Independent variables	(1)	(2)	(3)	(4)
(a) The R–C treatment dummy {= 1 for the R–C treatment; = 0 otherwise}	− 0.373* (0.206)	− 0.340* (0.202)	0.541* (0.294)	0.949** (0.428)
(b) The N–IC treatment dummy {= 1 for the N–IC treatment; = 0 otherwise}	2.103*** (0.328)	2.049*** (0.302)	− 0.616 (0.618)	− 1.506** (0.656)
(c) The R–IC treatment dummy {= 1 for the R–IC treatment; = 0 otherwise}	2.618*** (0.477)	2.577*** (0.445)	− 3.060*** (0.543)	− 3.774*** (0.479)
Value of commodity in period *t* (i.e., *q*_*t*_)	–	0.033 (0.063)	–	0.566*** (0.112)
Period = {1, 2, …, 50}	–	0.002 (0.007)	–	− 0.018 (0.015)
Constant	5.672*** (0.442)	4.940*** (1.292)	3.137*** (0.640)	− 7.321*** (2.119)
# of observations	8000	8000	8000	8000
Wald chi^2^	443.82	951.07	147.46	330.69
Prob > chi^2^	< 0.0001	< 0.0001	< 0.0001	< 0.0001
Two-sided *p* values (Wald Chi square test results)				
Test 1				
Ho: R–C = R–IC [i.e., variable (a) = variable (c)]	< 0.0001	< 0.0001	< 0.0001	< 0.0001
Test 2				
Ho: N–IC = R–IC [i.e., variable (b) = variable (c)]	0.2054	0.1687	0.0013	0.0017

Random-effects linear regressions with robust standard errors clustered by session ID. Numbers in parenthesis are standard errors

Control variables include a USA dummy (= 1 if sessions were conducted in the USA; 0 otherwise), a female dummy (= 1 if female; 0 otherwise), number of economics courses taken, general political orientation (1 = very conservative to 7 = very liberal) and income of the subject’s family. We omitted the coefficient estimates of these demographic variables to conserve space since these are not related to the hypotheses in the paper

*, **, and *** indicate significance at the 0.10 level, at the 0.05 level and at the 0.01 level, respectively

#### *Result 4*

Results 1 and 2 hold also when we test the impact of each treatment factor while controlling for the panel data structure.

## Conclusions

This paper investigated the effects of expressing emotions in a finitely repeated ultimatum game. In the treatments where both sellers and buyers were aware of the value of the commodity, sellers exhibited disapproval aversion with a rating system present. In contrast, buyers did not display greater acceptance of inequity in that condition. The picture changed drastically once buyers were uninformed of the value of the commodity. With the incomplete information setup, sellers no longer exhibited disapproval aversion, and they attempted to take much larger shares from buyers regardless of the presence of a rating system. Buyers, who were put in weaker positions, became more open to accepting unfair offers if a rating system was available.

As a final remark, we note that although our results are clear, there are many avenues for future research. For example, details of the experimental setups may affect the direction or degree of the effects of expressing emotions. For instance, the payoffs of buyers were negative if *p*_*s*_ > *q* in our design. Our setup is reasonable for a wide variety of circumstances, but it would also be a useful follow-up study to examine the same question in a setup where sellers are required to split the pie so that both sellers and buyers obtain non-negative payoffs. Second, it would also be useful to perform a robustness check using different games, such as prisoner’s dilemma games, to establish the behavioral regularity of our findings. It is possible that the relative strength of disapproval aversion may differ in other games. Finally, needless to say, more replication studies are essential as results may depend on various factors such as culture and populations, although we found similar patterns between the USA and Taiwan.

## Electronic supplementary material

Below is the link to the electronic supplementary material.

Supplementary material 1 (PDF 544 kb)

Supplementary material 2 (PDF 641 kb)

## Footnotes

1 For example, see Masclet et al. (2003) and Dugar (2013) in public goods games, Ellingsen and Johannesson (2008) and Xiao and Houser (2009) in a dictator game, and López-Pérez and Vorsatz (2010) in a prisoner’s dilemma game.

2 See, for example, Straub and Murnighan (1995), Rapoport et al. (1996), Croson (1996), Güth et al. (1996) and Mitzkewitz and Nagel (1993).

3 This design was employed because our aim was to measure the effects of a rating system itself without reputation effects.

4 In the experimental sessions, two subsets of subjects are called “buyers” and “sellers” as written in the paper. The framing of buyers and sellers is often used in experiments with ultimatum games (e.g., Roth et al. 1991).

5 Strategy methods are widely used in experiments with ultimatum games. The benefit of using strategy methods is the ability to elicit the upper bound of the buyer’s purchase decision (acceptance). With the standard sequential direct-response method, we only observe a buyer’s acceptance to a specific offer, not the threshold. Past studies have also found that there is no difference in the mean offer or mean acceptance rate between the two methods with complete information. They also indicate little difference in subjects’ behavior between the two methods when used in ultimatum games with incomplete information on the buyer's side (see Brandts and Charness 2011 for a survey).

6 The participation fee is guaranteed even if a subject’s accumulated payoff is negative. In the experiment, 0, 0, 11.25 and 18.75% of the buyers in the N–C, R–C, N–IC and R–IC treatments, respectively, received only participation fees due to their negative accumulated payoffs.

7 This setup of the incomplete information condition was used in past research such as Rapoport et al. (1996). For example, in Rapoport et al. (1996), the size of the pie in an ultimatum game is randomly distributed from a uniform distribution [*a*, *b*] and the realized size is information known only to the sellers.

8 Although we use a discrete interval {0, 1, …, 40} for the choice space in the experiment, we use a continuous interval [0, 40] for simplicity in our theoretical analysis.

9 For instance, the following is an equilibrium: the seller proposes *p*_*s*_ = $p_{s} \left( {q_{t} } \right) = c$ for $q_{t} \le 2c$; and $p_{s} \left( {q_{t} } \right) = q_{t}$ for $q_{t} > 2c$, and the buyer submits *p*_*b*_ = *c* as her purchase threshold. Here, *c* is any constant that is less than or equal to 20. Although there are multiple BNE of this class, the expected payoff for the seller differs by equilibrium and is maximized when the following equilibrium is realized: *p*_*s*_ = $p_{s} \left( {q_{t} } \right) = 20$ for all *q*_*t*_, and *p*_*b*_ = 20. The expected payoff for the seller is then $10$. This implies that the Pareto dominant BNE is that the seller always proposes to sell the commodity at a price of 20, and the buyer sets the purchase threshold at the price of 20.

10 Our choice of a quadratic function is due to its tractability, but we would not lose many important implications because of this choice. Quadratic functional forms are sometimes used in theoretical analyses of subjects’ behaviors. For example, see Cappelen et al. (2013) and Kamei (2016).

11 The seller’s best offering price is increasing in *q*_*t*_, but with a slope of less than 1, when *q*_*t*_ is sufficiently smaller than the seller’s belief regarding the buyer’s purchase threshold, as the seller prefers fairer outcomes. If a realized *q*_*t*_ is in some range close to a given buyer’s purchase threshold *p*_*bi*_, the seller attempts to submit *p*_*bi*_ as his offering price, although the seller offers a price strictly greater than *p*_*bi*_ when *q*_*t*_ is sufficiently large (Appendix Figure A.2). The buyer, given *p*_*sj*_, attempts to submit *p*_*sj*_ as her purchase threshold, as long as the buyer’s utility is non-negative.

12 Another other-regarding preference model is an intention-based social preference model such as a reciprocity model (e.g., Rabin 1993; Charness and Rabin 2002; Dufwenberg and Kirchsteiger 2004; Falk and Fischbacher 2006; Cox et al. 2007). In a reciprocity model, agents react hostilely to hostile acts taken toward them by their opponents. In our experiment, buyers have the opportunity to reject unfair offers proposed by sellers. Thus, such a reciprocity model would also predict a fairer division of the potential gain between the two parties.

13 Adding a show-up fee in the payoff function of a player does not change the calculation. For notational simplicity, we did not include the show-up fee in the payoff functions.

14 When it is not closed, the buyer gives a rating of 5 (the neutral rating) to the seller.

15 Buyers can cancel out the negative emotions from disappointment by releasing them through rating acts.

16 Both buyers and sellers would change their action choices over time even if they have stable inequality-averse preferences as the design of each treatment is based on the ultimatum game with a strategy method and has multiple equilibria. As in the discussion in Cooper and Dutcher (2011), the subjects’ learning process is determined by the distribution of their beliefs regarding others’ behaviors if they hold inequality-averse or reciprocal preferences. The reinforcement learning theory may also account for the learning behaviors of some subjects.

17 We first wrote the instructions in English. Chen and her research assistant (both native speakers of Mandarin) translated them carefully to Mandarin so that there was no wording with positive or negative connotations.

18 They were recruited by solicitation emails via the BUSSEL (Brown University Social Science Experimental Laboratory) for the USA sessions, and via TASSEL (Taiwan Social Science Experimental Laboratory) for the Taiwan sessions. The sessions in the USA were conducted from July to September 2013 and July and August in 2016. The sessions in Taiwan were conducted in September and October 2016. The numbers of female subjects were 79 (49.38% of the subjects) for the USA sessions and 58 (36.25% of the subjects) for the Taiwan sessions. The numbers of subjects with economics majors were 28 (17.50% of the subjects) for the USA sessions and 25 (15.63% of the subjects) for the Taiwan sessions. Subjects were privately paid immediately at the end of the session. The average earnings (including show-up fee) were $17.0 in the USA sessions and 338.3 NT dollars (around $10.6) in the Taiwan sessions. We note that the minimum hourly wage in Taiwan was 133 NT dollars as of September 2016.

19 This feature was employed due to its simplicity. Nevertheless, this feature may have potentially caused subjects’ behaviors within sessions to become correlated with each other (e.g., Fréchette 2012). We include session clustering or use bootstrap standard errors when we perform the regression analyses in Sects. 4.2 and 4.3.

20 The null hypothesis that sellers’ average keep shares are equal to 1 is rejected in each of the four comparisons, according to one-sided *t* tests (*p* < .0001, *p* < .001, *p* < .0001, and *p* < .01 in the Taiwan N–IC, the USA N–IC, the Taiwan R–IC, and the USA R–IC sessions, respectively).

21 38.0% (32.2%) and 33.3% (42.2%) of the sellers’ offering prices were greater than *q* in the N–IC and R–IC treatments, respectively, for the USA (Taiwan) sessions. These percentages are significantly higher than the same percentages seen in the corresponding complete information treatments, which are 4.1% (1.8%) and 4.5% (7.1%), for the USA (Taiwan) sessions, according to two-sided chi-squared tests (*p* value < .001). We note that the percentage of events in which buyers received negative payoffs is significantly larger in the N–IC (R–IC) than in the N–C (R–C) treatment, regardless of the subject pool (*p* value < .001, two-sided chi-squared test).

22 The sellers’ average realized shares are significantly different between the N–C and N–IC treatments in the USA (*p* *<* .0001) and in Taiwan (*p* *<* .0001), according to two-sided Mann–Whitney tests. Likewise, there are significant differences between the R–C and R–IC treatments in the USA (*p* *<* .0001) and in Taiwan (*p* *<* .0001).

23 The difference in the average acceptance rate between the N–C and N–IC treatments is significant for each of the USA and Taiwan sessions, according to a Mann–Whitney test based on the average rates where sellers’ offers were accepted over 50 periods (*p* value < .001, two-sided). The same holds for a comparison between the R–C and R-IC treatments, whether with the USA subjects or Taiwan subjects. See also Appendix Table B.1.

24 The levels of acceptance rates were high from the onset, and then rose from period to period at small rates in all treatments (Appendix Table B.1). This suggests that the acceptable ranges of buyers largely coincided with those of sellers from earlier periods and they just gradually needed to adjust their action choices over time.

25 A two sided Mann–Whitney test, based on pooled data of sellers’ average decisions, found that the difference in sellers’ keep share is significant between the N–C and R–C treatments at *p* = .0220.

26 A two sided Mann–Whitney test, based on pooled data of buyers’ average decisions, found that the difference in buyers’ keep (keep share) is significant between the N-IC and R-IC treatments at *p* = .0138 (*p* = .0262).

27 The buyer’s payoff or share based on the seller’s keep was not included as an independent variable because it is a linear transformation of the seller’s keep or keep share.

28 To supplement Table 2, we also estimated the Heckman two-stage selection model, with the dependent variable in the first stage being whether transactions were closed or not. This model has an advantage in that we can allow coefficients of all the independent variables in the second stage to be different by whether deals were closed or not. We used buyers’ last period purchase thresholds as an instrument. Results we obtained in this additional analysis were qualitatively similar to that of Table 2. See Appendix Table B.2.

29 We did not include the interaction terms between the period number variable and the treatment dummies as independent variables because of two reasons. First, none of the interaction terms obtain significant coefficients even if we include them. Second, we suffer from serious colinearity problems if we add these terms. The variance inflation factors for variables (a), (b) and (c) are much larger than 5 if these interaction terms are included.
