# Supplementary material for: Disapproval aversion or inflated inequity acceptance? The impact of expressing emotions in ultimatum bargaining
Source: Exp Econ. 2017 Nov 13;21(4):836–57. doi: 10.1007/s10683-017-9554-z (PMC6223739; doi:10.1007/s10683-017-9554-z)
Supplement: Supplementary file 1 — Supplementary material 1 (PDF 544 kb) [file 10683_2017_9554_MOESM1_ESM.pdf]

Supplementary Online Instruction Appendix for

Chen and Kamei, 2017,

"Disapproval Aversion or Inflated Inequity Acceptance?
The Impact of Expressing Emotions in Ultimatum Bargaining"[1]

This Appendix contains the full sets of instructions used in Chen and Kamei (2017).

Table of Contents

Part A: The Instructions in English

A.1. Instructions of the N-C Treatment .... p. 2

A.2. Instructions of the R-C Treatment .... p. 7

A.3. Instructions of the N-IC Treatment .... p. 13

A.4. Instructions of the R-IC Treatment .... p. 18

Part B: The Instructions Translated into Mandarin

B.1. Instructions of the N-C Treatment .... p. 24

B.2. Instructions of the R-C Treatment .... p. 29

B.3. Instructions of the N-IC Treatment .... p. 34

B.4. Instructions of the R-IC Treatment .... p. 38

[1] Correspondence: Kenju Kamei, Department of Economics and Finance, Durham University, Mill Hill Lane, Durham, DH1 3LB, United Kingdom. Email: kenju.kamei@gmail.com, kenju.kamei@durham.ac.uk.

Part A: The Instructions in English

A.1. Instructions of the N-C Treatment

Instructions:

You are now participating in a decision-making experiment in which you will earn an amount of money that depends on your decisions and on the decisions of other participants. During the experiment your earnings will be calculated in points. At the end of the experiment, points will be converted to U.S. dollars at the following rate:

14 points = $1

(This means each point will exchange for around 7 cents of real money)

In this experiment, $5 is guaranteed for your participation. That is, your today's payment is the greater of $5 or $5 plus the converted earnings in the session. At the end of the experiment, your total earnings will be paid out to you in cash. Your decisions are anonymous.

The experiment has 50 periods in total. There are 20 participants in total.

At the onset of the experiment, each individual including you, will be randomly assigned to a group of 10 participants, consisting of five "buyers" and "sellers." The roles of buyer and seller will also be assigned randomly. That is, you will be assigned either role with a probability of 50%. During the experiment, you will engage in a series of interactions, each of which will involve you and one other participant in your group. Your initial assigned role, either buyer or seller, will not change throughout the session. You will not interact with participants in the other group in the experiment.

At the onset of each period, each buyer will be matched with a seller. A buyer will not be matched with more than one seller. The matching is completely random: the probability that a buyer is matched with a specific seller is exactly 1/5, since there are five sellers in each group.

You will make the same kinds of decisions every period. The following instructions explain the details of a randomly chosen period, period *t*.

In period *t*, each seller holds an identical object whose value is different by period. The value of the object, *q*, is randomly drawn from a set of integers, {0, 1, 2, …, 40}, for each group of 10 participants. That is, each number is equally likely to be drawn: the probability that a specific number, say 12, is chosen as the value of an object equals 1/41. Neither your previous choices

nor the previous values of the objects affect the random drawing process. After the value of the object is determined, it will be informed to both sellers and buyers.
Sellers then propose prices, *p*, to sell the object to their matched buyers. Each seller can choose any integer from {0, 1, …, 40} as the price (*p*) of the object. While sellers are in the process of choosing their prices, buyers are asked to choose their threshold of purchasing the object, *x*, from any integer {0, 1, …, 40}. Once all buyers and sellers make their decisions, their transactions will be exerted in pairs.

An example of the computer screen for a seller appears below:

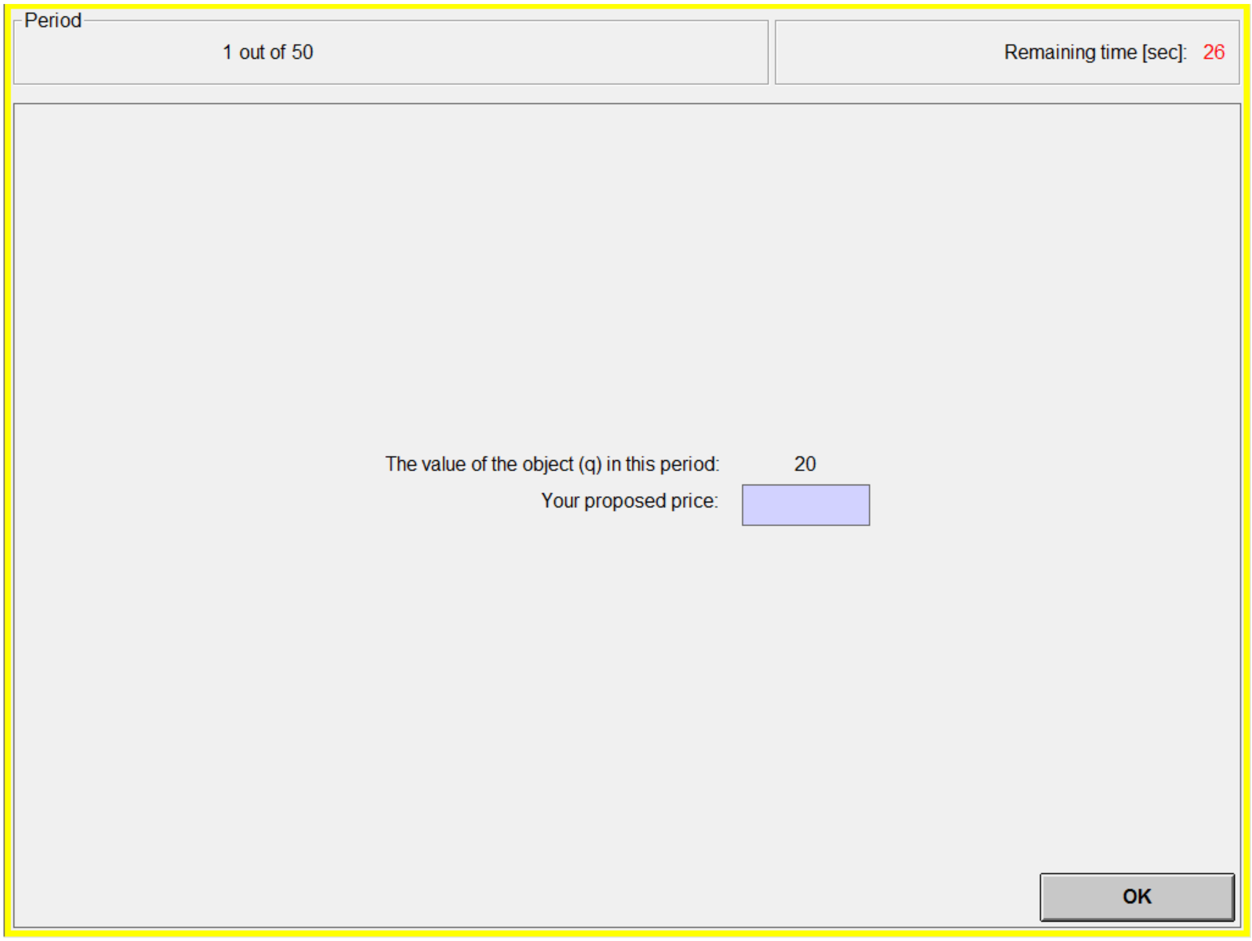

Note: Numbers shown are illustrations only.

An example of the computer screen for a buyer appears below:

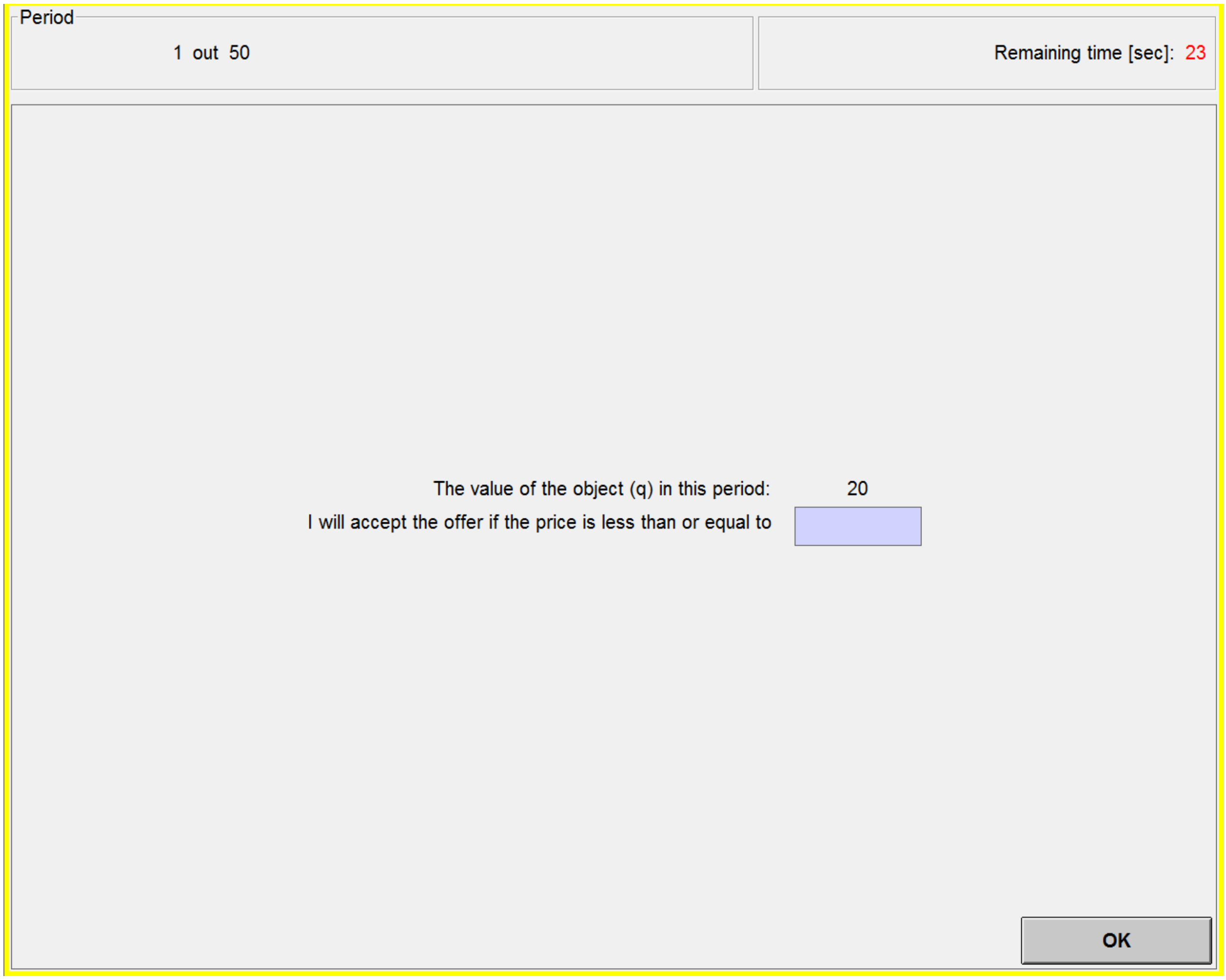

Note: Numbers shown are illustrations only.

If $p \leq x$, then, the transaction will be completed, and the buyer receives:

$$q - p,$$

and the seller receives:

$$p - \frac{1}{2} \cdot q.$$

Your points (earnings) will be accumulated over periods. If you have negative earnings in a period, then they will be deducted from those you gain in other periods.

By contrast, if $p > x$, then the transaction will not be completed, and both the buyer and seller receive nothing.

Example: Suppose that the value of the object is 20, and the seller proposes to sell the object at a price of 14 in period 5. If his or her matched buyer's upper limit of buying price is 16, which is

higher than 14, then the transaction is completed, and he or she receives earnings of 6 (= 20 – 14), and the seller obtains 4 (= 14 – ½ * 20). If the buyer's upper limit of buying price is 5, which is less than 14, then, their transaction will not be completed. In that case, both the buyer and seller receive nothing during that period.

Once all buyers make their decisions (accept or reject), you are informed of: whether or not the offer is accepted in your pair and both of your and your partner's earnings. Once everyone in a session has reviewed their transaction results, you will move on to the next period, $t + 1$. In period $t + 1$ as well, each buyer is randomly matched with a seller in your group, and the value of an object is randomly drawn from the set, $\{0, 1, 2, \ldots, 40\}$. These random drawing processes are not affected by your period t's decisions or your transaction results.

Once 50 rounds of interactions are completed, the main part of the experiment is over. You will be asked to answer several questions about yourself, such as gender and year in school. Neither the information about your decisions during the experiment nor your responses to these questions can be linked to you as an individual.

At the end of the experiment, your accumulated points will be converted to US dollars, and you will privately receive your payment in cash. You will not know how much others earn in the experiment; nor will the other participants know your payment in the experiment.

Comprehension Question:

Before we begin, please respond to the following questions in order for you to check your understanding. At this time, please raise your hands if you have any questions. When we confirm that every participant has correctly answered the questions, we will start the experiment.

1. How many periods are there in the experiment?

2. Out of the 10 participants in your group, how many of them are assigned the role of "buyer"?

Note: As explained, each subject's role, either buyer or seller, does not change during the session.

3. Suppose that you are a "seller," and have been matched with a buyer in period 5. What is the probability that you will be matched with the same buyer in period 6?

4. Suppose that you're assigned the role of "buyer." Your matched seller proposes selling an object at a particular price, and you will submit the upper limit of your buying price. Which of the following statement is correct, concerning the value of the object, when you are making your decisions?

(a) Both you and the seller are informed of the value of the object.

(b) Only the seller is informed of the value of the object.

(c) Only you are informed of the value of the object.

(d) Neither you nor the seller is informed of the value of the object.

A.2. Instructions of the R-C Treatment

Instructions:

You are now participating in a decision-making experiment in which the amount of money you earn will depend on your decisions and on the decisions of other participants. During the experiment your earnings will be calculated in points. At the end of the experiment, points will be converted to U.S. dollars at the following rate:

14 points = $1

(This means each point will exchange for around 7 cents of real money)

In this experiment, $5 is guaranteed for your participation. That is, your today's payment is the greater of $5 or $5 plus the converted earnings in the session. At the end of the experiment, your total earnings will be paid out to you in cash. Your decisions are anonymous.

The experiment has 50 periods in total. There are 20 participants in total.

At the onset of the experiment, each individual including you will be randomly assigned to a group of 10 participants, consisting of five "buyers" and "sellers." The roles of buyer and seller will also be assigned randomly. That is, you will be assigned either role with a probability of 50%. During the experiment, you will engage in a series of interactions, each of which will involve you and one other participant in your group. Your initial assigned role, either buyer or seller, will not change throughout the session. You will not interact with participants in the other group during the experiment.

At the onset of each period, each buyer will be matched with a seller. A buyer will not be matched with more than one seller. The matching is completely random: the probability that a buyer is matched with a specific seller is exactly 1/5, since there are five sellers in each group.

You will make the same kinds of decisions every period. The following instructions explain the details of a randomly chosen period, period *t*.

In period *t*, each seller holds an identical object whose value is different by period. The value of the object, *q*, is randomly drawn from a set of integers, {0, 1, 2, …, 40}, for each group of 10 participants. That is, each number is equally as likely to be drawn: the probability that a specific number, say 12, is chosen as the value of an object equals 1/41. Neither your previous choices nor the previous values of the objects affect the random drawing process. After the value of the object is determined, it will be informed to both sellers and buyers.

Sellers then propose prices, p, to sell the object to their matched buyers. Each seller can choose any integer from $\{0, 1, \ldots, 40\}$ as the price (p) of the object. While sellers are in the process of choosing their prices, buyers are asked to choose their threshold of purchasing the object, x, from any integer $\{0, 1, \ldots, 40\}$. Once all buyers and sellers make their decisions, their transactions will be exerted in pairs.

An example of the computer screen for a seller appears below:

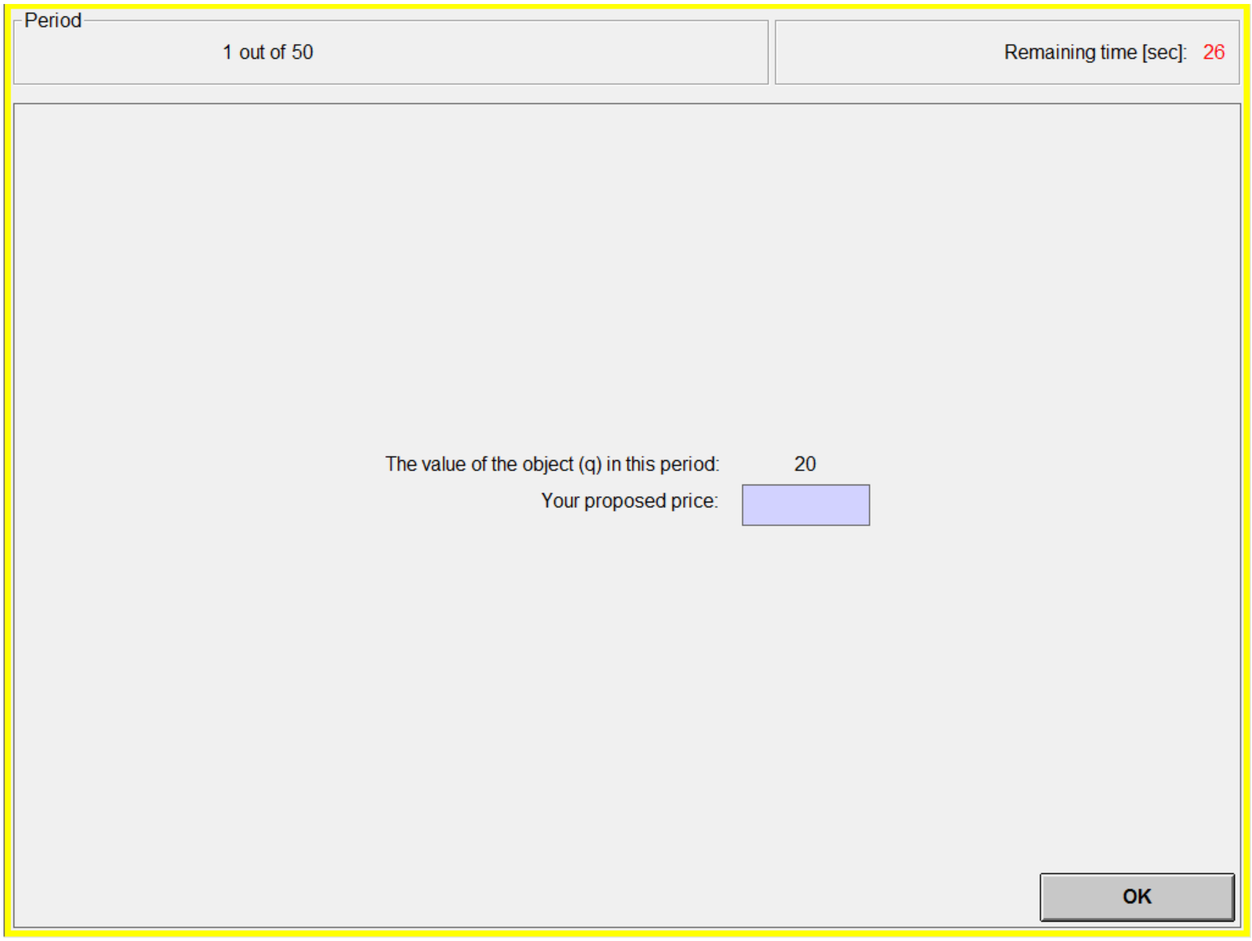

Note: Numbers shown are illustrations only.

An example of the computer screen for a buyer appears below:

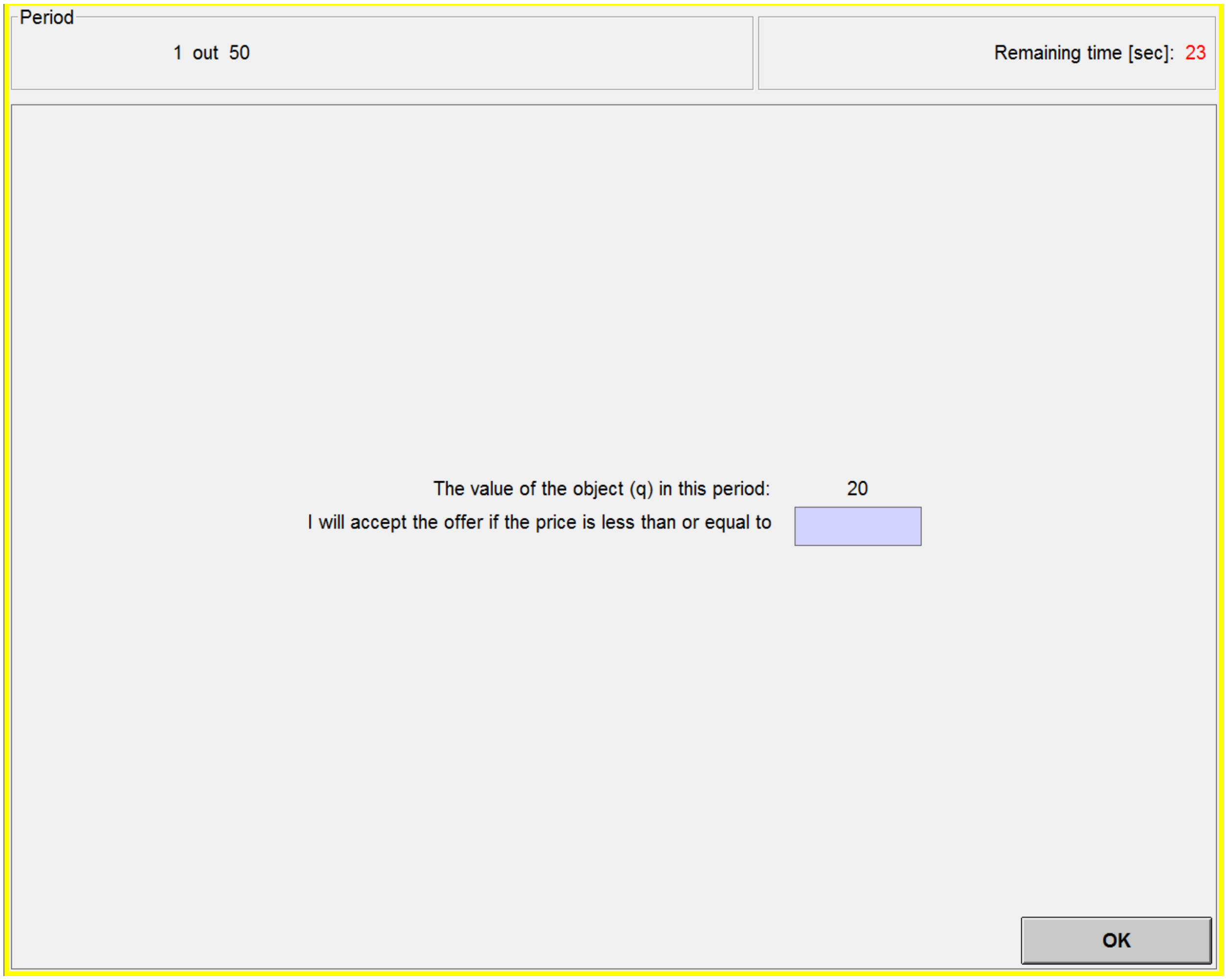

Note: Numbers shown are illustrations only.

If $p \leq x$, then, the transaction will be completed, and the buyer receives:

$$q - p,$$

and the seller receives:

$$p - \frac{1}{2} \cdot q.$$

Your points (earnings) will be accumulated over periods. If you have negative earnings in a period, then they will be deducted from those you gain in other periods.

By contrast, if $p > x$, then the transaction will not be completed, and both the buyer and seller receive nothing.

Example: Suppose that the value of the object is 20, and the seller proposes to sell the object at a price of 14 in period 5. If his or her matched buyer's upper limit of buying price is 16, which is

higher than 14, then the transaction is completed, and he or she receives earnings of 6 (= 20 – 14), and the seller obtains 4 (= 14 – ½ * 20). If the buyer's upper limit of buying price is 5, which is less than 14, then, their transaction will not be completed. In that case, both the buyer and seller receive nothing during that period.

Once all buyers make their decisions (accept or reject), you are informed of: whether or not the offer is accepted in your pair and both of your and your partner's earnings. In this stage, buyers are asked to give feedback on their matched sellers' proposed prices by choosing an integer from {0, 1, 2, 3, 4, 5, 6, 7, 8, 9, 10}.The lowest number, 0, means "very unfair," 3 means "unfair," 7 means "fair," and the highest number, 10, means "very fair." The feedback that a buyer gives neither affects his or her earnings nor does it affect the matched seller's earnings. While buyers are in the process of giving feedback, sellers are asked about their potential selling price if they have an opportunity to sell an object whose value is the same as in period *t* in a later period. The sellers' responses are hypothetical, and do not affect their or their matched buyers' earnings in period *t*; nor is their decision binding in the later periods.

An example of the computer screen for a buyer appears below:

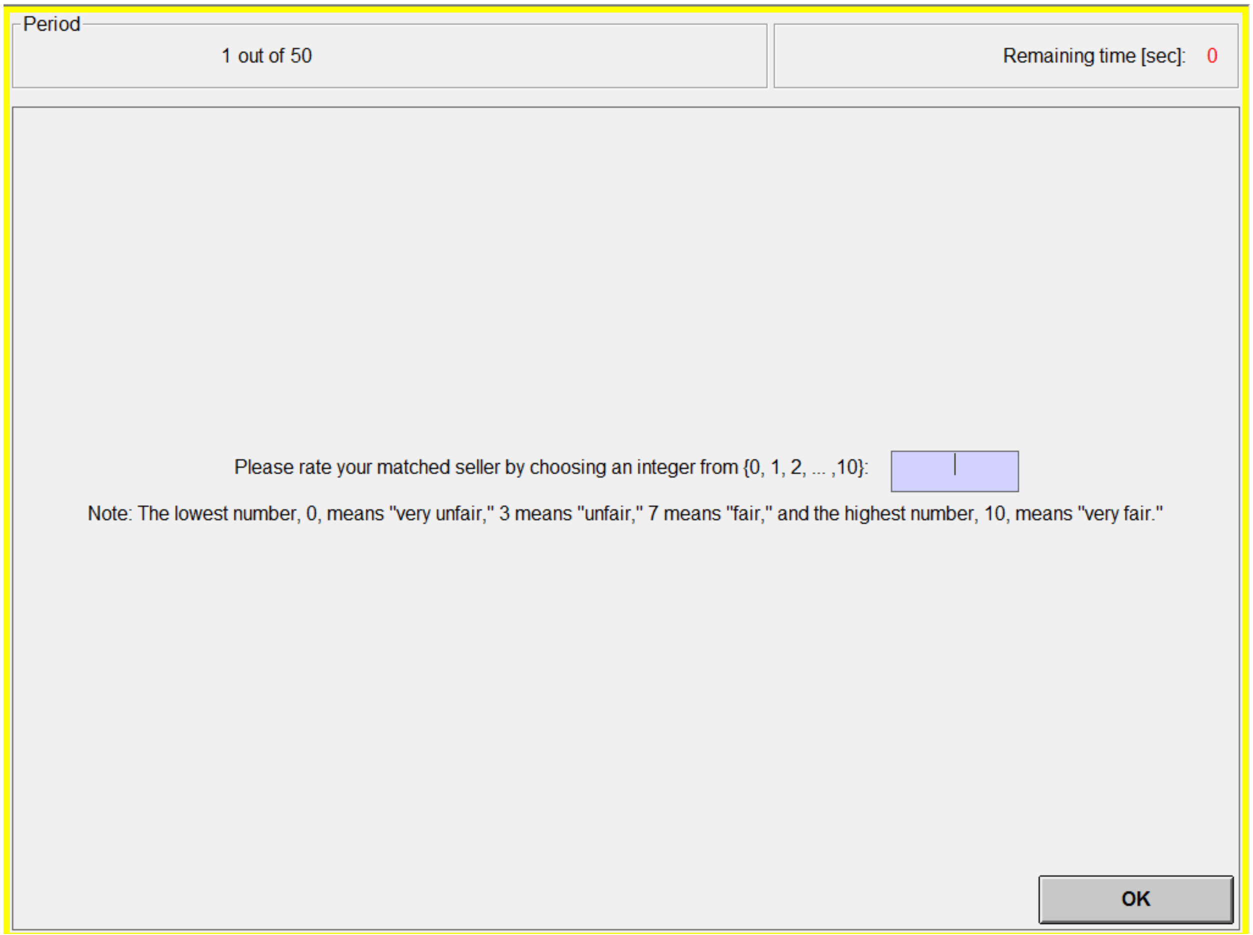

Once all buyers and sellers submit their answers, each seller is informed of the rating given by their matched buyer.

Once everyone in a session has reviewed their feedback, you will move on to the next period, *t* + 1. In period *t* +1 as well, each buyer is randomly matched with a seller in your group, and the value of an object is randomly drawn from the set, {0, 1, 2, …, 40}. These random drawing processes are not affected by your period *t*'s decisions or your transaction results.

Once 50 rounds of interactions are completed, the main part of the experiment is over. You will be asked to answer several questions about yourself, such as gender and year in school. Neither information about your decisions during the experiment nor your responses to these questions can be linked to you as an individual.

At the end of the experiment, your accumulated points will be converted to US dollars, and you will privately receive your payment in cash. You will not know how much others earn in the experiment; nor will the other participants know your payment in the experiment.

Comprehension Question:

Before we begin, please respond to the following questions in order for you to check your understanding. At this time, please raise your hands if you have any questions. When we confirm that every participant has correctly answered the questions, we will start the experiment.

1. How many periods are there in the experiment?

2. Out of the 10 participants in your group, how many of them are assigned the role of "buyer"?
   Note. As explained, each subject's role, either buyer or seller, does not change during the session.

3. Suppose that you are a "seller". What is the probability that you will be matched with the same buyer in both of the current period and the next period? [ ]

4. Suppose that you're assigned the role of "buyer." Your matched seller proposes to sell an object at some price, and you will submit the upper limit of your buying price. Which of the following statement is correct, concerning the value of the object, when you are making the decisions?

   (a) Both you and the seller are informed of the value of the object.
   (b) Only the seller is informed of the value of the object.
   (c) Only you are informed of the value of the object.
   (d) Neither you nor the seller is informed of the value of the object.

5. At the end of each period, each buyer rates their matched seller on a 10-point scale. Which of the following statement is correct concerning the rating that a buyer gives?

   (a) Only the matched seller is informed of the rating.
   (b) All participants in the session are informed of the rating.
   (c) Both the matched seller and the other four sellers in his or her group are informed of the rating.
   (d) None in the session is informed of the rating.

A.3. Instructions of the N-IC Treatment

Instructions:

You are now participating in a decision-making experiment in which you will earn an amount of money that depends on your decisions and on the decisions of other participants. During the experiment your earnings will be calculated in points. At the end of the experiment, points will be converted to U.S. dollars at the following rate:

14 points = $1

(This means each point will exchange for around 7 cents of real money)

In this experiment, $5 is guaranteed for your participation. That is, your today's payment is the greater of $5 or $5 plus the converted earnings in the session. At the end of the experiment, your total earnings will be paid out to you in cash. Your decisions are anonymous.

The experiment has 50 periods in total. There are 20 participants in total.

At the onset of the experiment, each individual including you, will be randomly assigned to a group of 10 participants, consisting of five "buyers" and "sellers." The roles of buyer and seller will also be assigned randomly. That is, you will be assigned either role with a probability of 50%. During the experiment, you will engage in a series of interactions, each of which will involve you and one other participant in your group. Your initial assigned role, either buyer or seller, will not change throughout the session. You will not interact with participants in the other group in the experiment.

At the onset of each period, each buyer will be matched with a seller. A buyer will not be matched with more than one seller. The matching is completely random: the probability that a buyer is matched with a specific seller is exactly 1/5, since there are five sellers in each group.

You will make the same kinds of decisions every period. The following instructions explain the details of a randomly chosen period, period t.

In period t, each seller holds an identical object whose value is different by period. The value of the object, q, is randomly drawn from a set of integers, $\{0, 1, 2, \ldots, 40\}$, in this session. That is, each number is equally likely to be drawn: the probability that a specific number, say 12, is chosen as the value of an object equals 1/41. Neither your previous choices nor the previous values of the objects affect the random drawing process. After the value of the object is determined, it will be informed to sellers, but not to buyers.

Sellers then propose prices, p, to sell the object to their matched buyers. Each seller can choose any integer from {0, 1, …, 40} as the price (p) of the object. While sellers are in the process of choosing their prices, buyers are asked to choose their threshold of purchasing the object, x, from any integer {0, 1, …, 40}. Once all buyers and sellers make their decisions, their transactions will be exerted in pairs.

An example of the computer screen for a seller appears below:

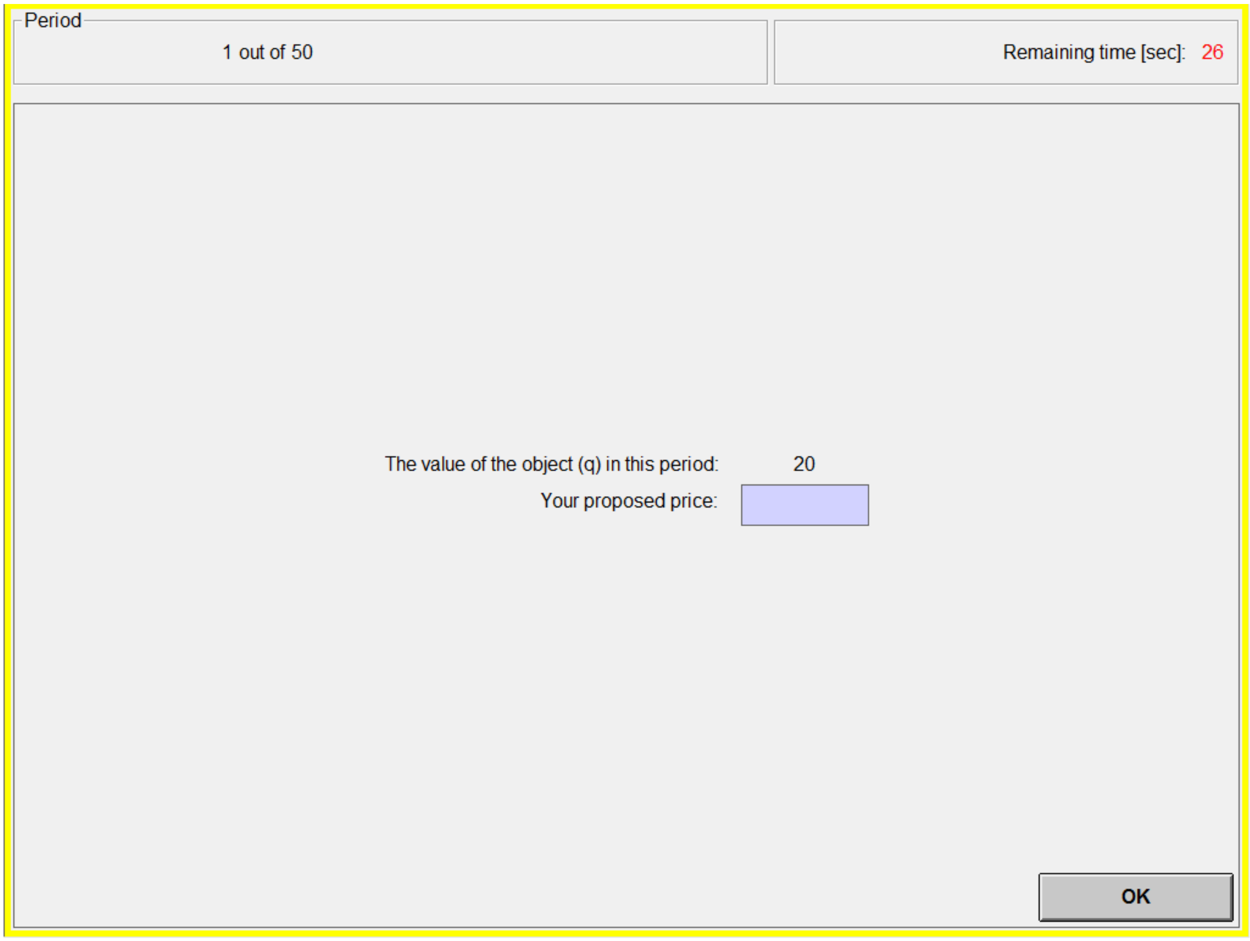

Note: Numbers shown are illustrations only.

An example of the computer screen for a buyer appears below:

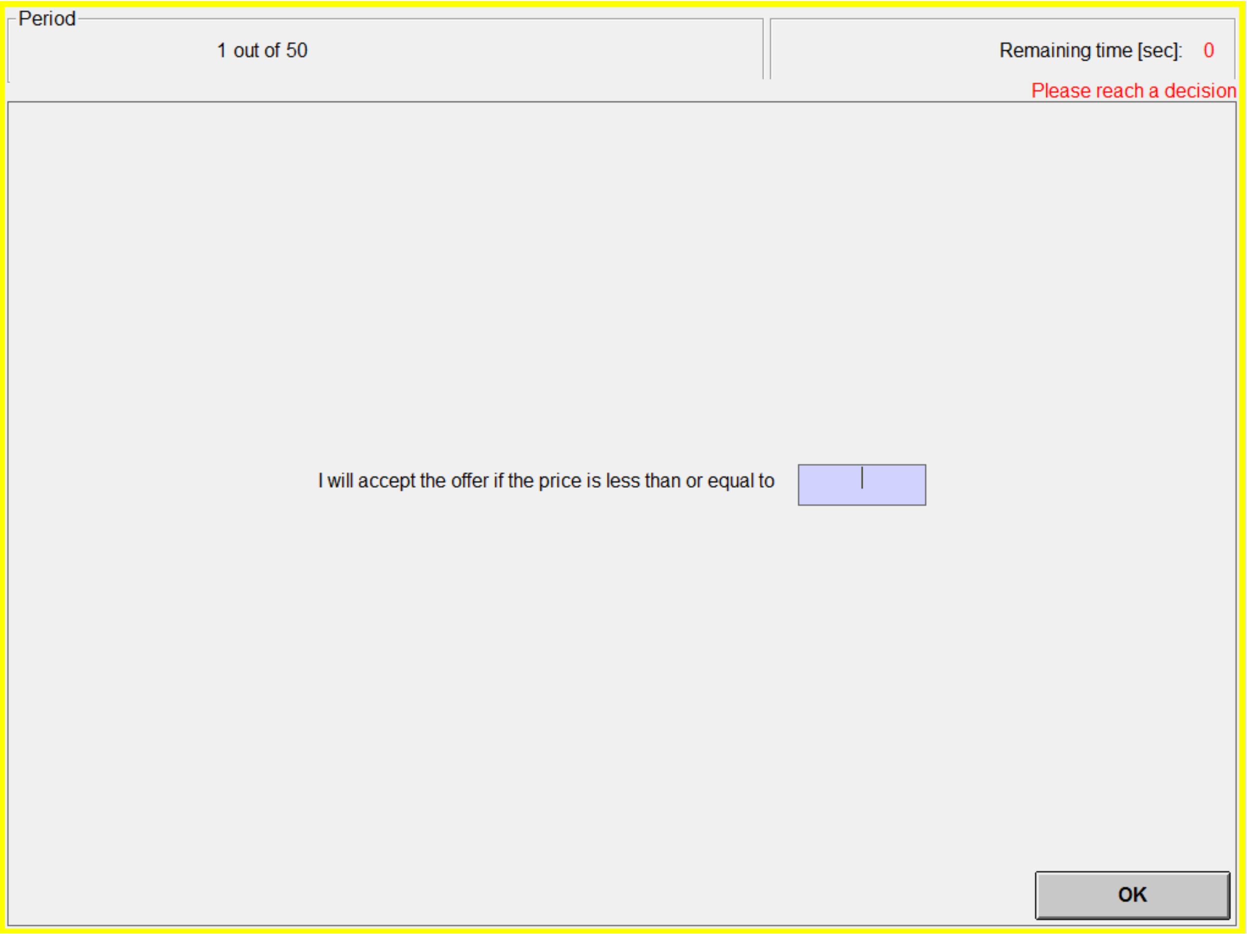

If $p \leq x$, then, the transaction will be completed, and the buyer receives:

$$q - p,$$

and the seller receives:

$$p - \frac{1}{2} \cdot q.$$

Your points (earnings) will be accumulated over periods. If you have negative earnings in a period, then they will be deducted from those you gain in other periods.

By contrast, if $p > x$, then the transaction will not be completed, and both the buyer and seller receive nothing.

Example: Suppose that the value of the object is 20, and the seller proposes to sell the object at a price of 14 in period 5. If his or her matched buyer's upper limit of buying price is 16, which is higher than 14, then the transaction is completed, and he or she receives earnings of 6 (= 20 – 14), and the seller obtains 4 (= 14 – ½ * 20). If the buyer's upper limit of buying price is 5,

which is less than 14, then, their transaction will not be completed. In that case, both the buyer and seller receive nothing during that period.

Once all buyers make their decisions (accept or reject), you are informed of: the value of the object, whether or not the offer is accepted in your pair, and both your and your partner's earnings. Once everyone in a session has reviewed their transaction results, you will move on to the next period, $t + 1$. In period $t + 1$ as well, each buyer is randomly matched with a seller in your group, and the value of an object is randomly drawn from the set, $\{0, 1, 2, \ldots, 40\}$. These random drawing processes are not affected by your period *t*'s decisions or your transaction results.

Once 50 rounds of interactions are completed, the main part of the experiment is over. You will be asked to answer several questions about yourself, such as gender and year in school. Neither the information about your decisions during the experiment nor your responses to these questions can be linked to you as an individual.

At the end of the experiment, your accumulated points will be converted to US dollars, and you will privately receive your payment in cash. You will not know about how much others earn in the experiment; nor will the other participants know your payment in the experiment.

Comprehension Question:

Before we begin, please respond to the following questions in order for you to check your understanding. At this time, please raise your hands if you have any questions. When we confirm that every participant has correctly answered the questions, we will start the experiment.

1. How many periods are there in the experiment?

2. Out of the 10 participants in your group, how many of them are assigned the role of "buyer"? Note: As explained, each subject's role, either buyer or seller, does not change during the session.

3. Suppose that you are a "seller," and have been matched with a buyer in period 5. What is the probability that you will be matched with the same buyer in period 6?

4. Suppose that you are assigned the role of “buyer.” Your matched seller proposes selling an object at a particular price, and you will submit the upper limit of your buying price. Which of the following statement is correct concerning the value of the object when you are making your decisions?
   (a) Both you and the seller are informed of the value of the object.
   (b) Only the seller is informed of the value of the object.
   (c) Only you are informed of the value of the object.
   (d) Neither you nor the seller is informed of the value of the object.

A.4. Instructions of the R-IC Treatment

Instructions:

You are now participating in a decision-making experiment in which the amount of money you earn will depend on your decisions and on the decisions of other participants. During the experiment your earnings will be calculated in points. At the end of the experiment, points will be converted to U.S. dollars at the following rate:

14 points = $1

(This means each point will exchange for around 7 cents of real money)

In this experiment, $5 is guaranteed for your participation. That is, your today's payment is the greater of $5 or $5 plus the converted earnings in the session. At the end of the experiment, your total earnings will be paid out to you in cash. Your decisions are anonymous.

The experiment has 50 periods in total. There are 20 participants in total.

At the onset of the experiment, each individual including you will be randomly assigned to a group of 10 participants, consisting of five "buyers" and "sellers." The roles of buyer and seller will also be assigned randomly. That is, you will be assigned either role with a probability of 50%. During the experiment, you will engage in a series of interactions, each of which will involve you and one other participant in your group. Your initial assigned role, either buyer or seller, will not change throughout the session. You will not interact with participants in the other group during the experiment.

At the onset of each period, each buyer will be matched with a seller. A buyer will not be matched with more than one seller. The matching is completely random: the probability that a buyer is matched with a specific seller is exactly 1/5, since there are five sellers in each group.

You will make the same kinds of decisions every period. The following instructions explain the details of a randomly chosen period, period t.

In period t, each seller holds an identical object whose value is different by period. The value of the object, q, is randomly drawn from a set of integers, $\{0, 1, 2, \ldots, 40\}$, in this session. That is, each number is equally as likely to be drawn: the probability that a specific number, say 12, is chosen as the value of an object equals 1/41. Neither your previous choices nor the previous values of the objects affect the random drawing process. After the value of the object is determined, it will be informed to sellers, but not to buyers.

Sellers then propose prices, p, to sell the object to their matched buyers. Each seller can choose any integer from {0, 1, …, 40} as the price (p) of the object. While sellers are in the process of choosing their prices, buyers are asked to choose their threshold of purchasing the object, x, from any integer {0, 1, …, 40}. Once all buyers and sellers make their decisions, their transactions will be exerted in pairs.

An example of the computer screen for a seller appears below:

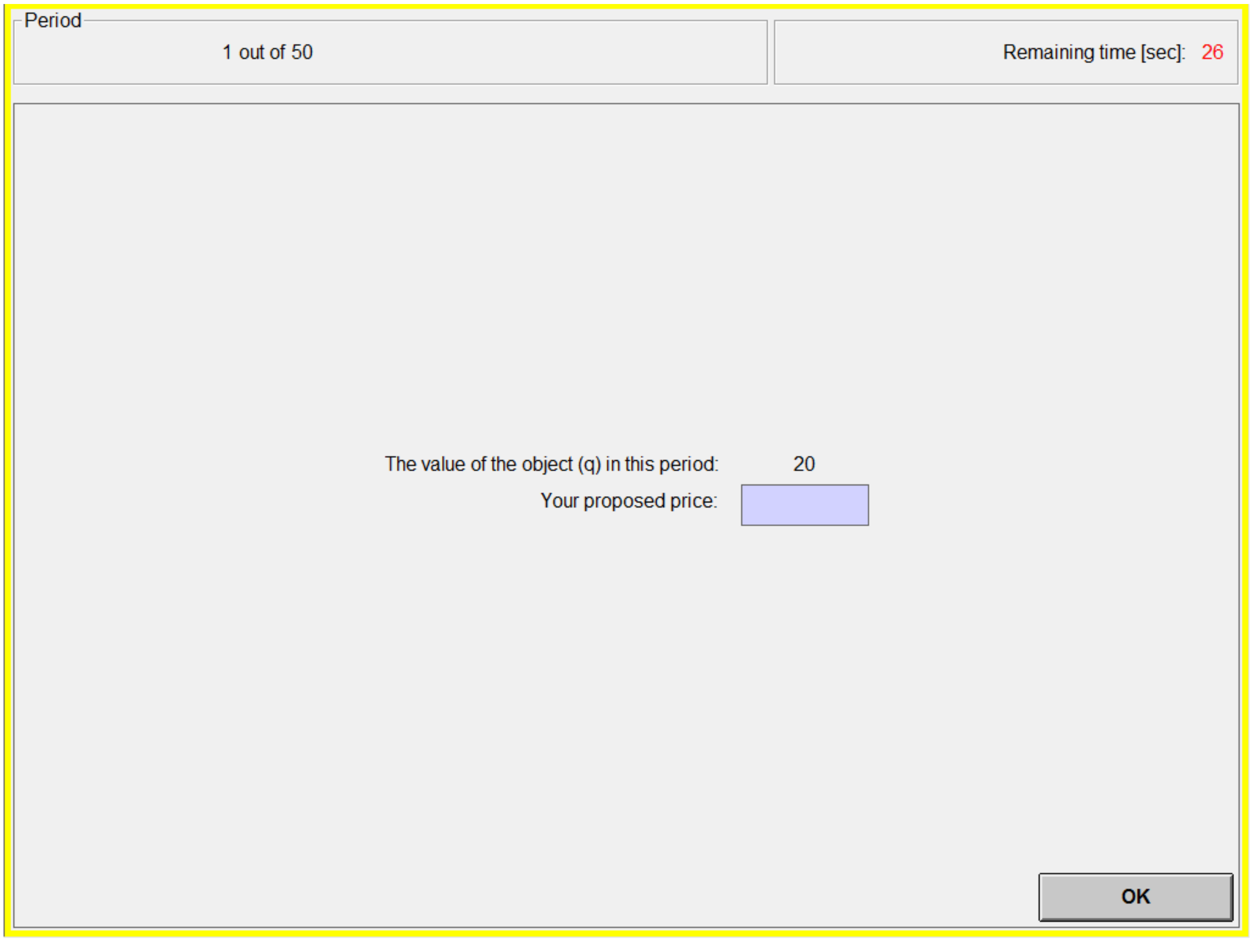

Note: Numbers shown are illustrations only.

An example of the computer screen for a buyer appears below:

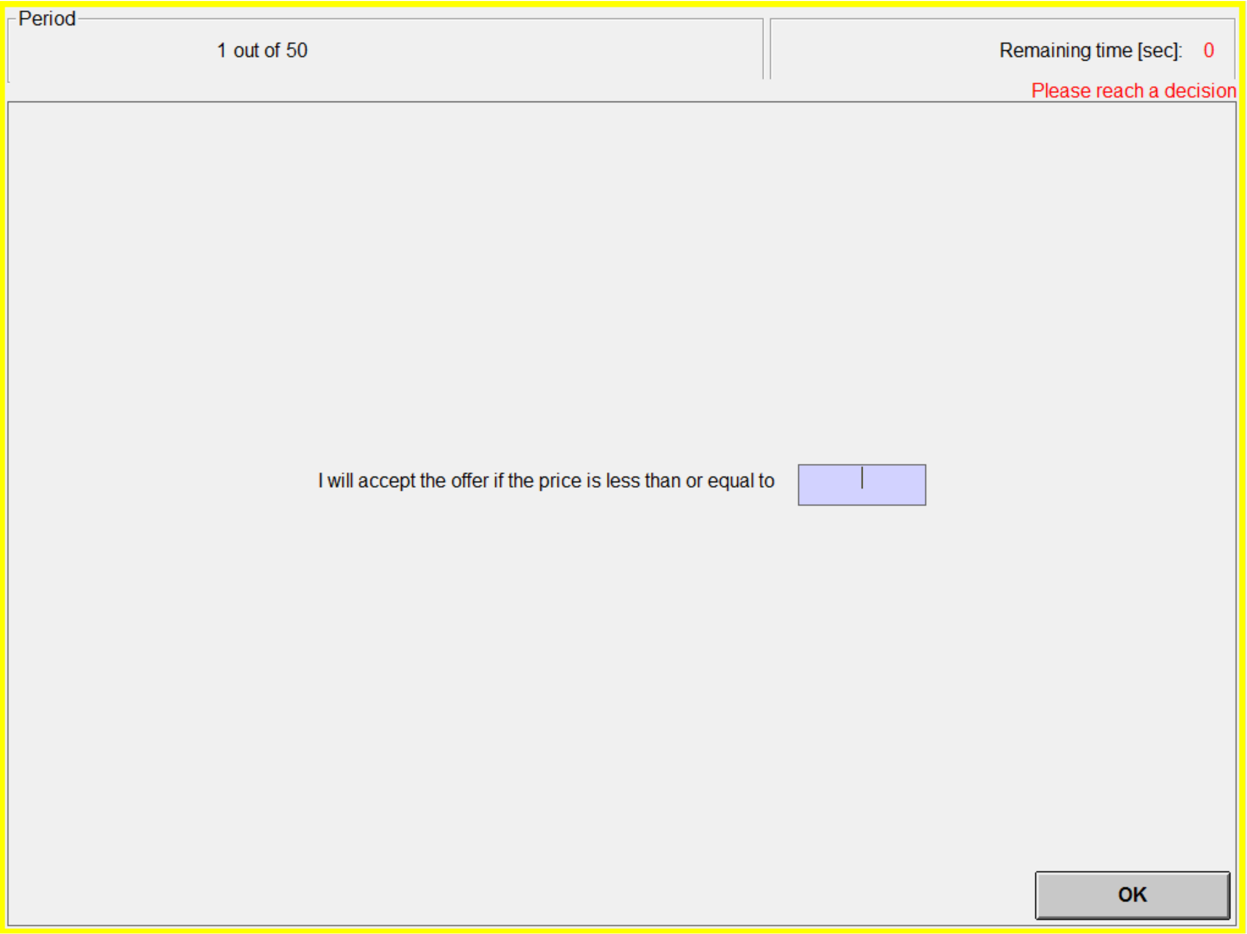

If $p \leq x$, then, the transaction will be completed, and the buyer receives:

$$q - p,$$

and the seller receives:

$$p - \frac{1}{2} \cdot q.$$

Your points (earnings) will be accumulated over periods. If you have negative earnings in a period, then they will be deducted from those you gain in other periods.

By contrast, if $p > x$, then the transaction will not be completed, and both the buyer and seller receive nothing.

Example: Suppose that the value of the object is 20, and the seller proposes to sell the object at a price of 14 in period 5. If his/ her matched buyer's upper limit of buying price is 16, which is higher than 14, then the transaction is completed, and he/ she receives earnings of 6 (= 20 – 14), and the seller obtains 4 (= 14 – ½ * 20). If the buyer's upper limit of buying price is 5, which is

less than 14, then, their transaction will not be completed. In that case, both the buyer and seller receive nothing during that period.

Once all buyers make their decisions (accept or reject), you are informed of: the value of the object, whether or not the offer is accepted in your pair, and both your and your partner's earnings. In this stage, buyers are asked to give feedback on their matched sellers' proposed prices by choosing an integer from {0, 1, 2, 3, 4, 5, 6, 7, 8, 9, 10}. The lowest number, 0, means "very unfair," 3 means "unfair," 7 means "fair," and the highest number, 10, means "very fair." The feedback that a buyer gives neither affects his/her earnings nor does it affect the matched seller's earnings. While buyers are in the process of giving feedback, sellers are asked about their potential selling price if they have an opportunity to sell an object whose value is the same as in period *t* in a later period. The sellers' responses are hypothetical, and do not affect their or their matched buyers' earnings in period *t*; nor is their decision binding in the later periods.

An example of the computer screen for a buyer appears below:

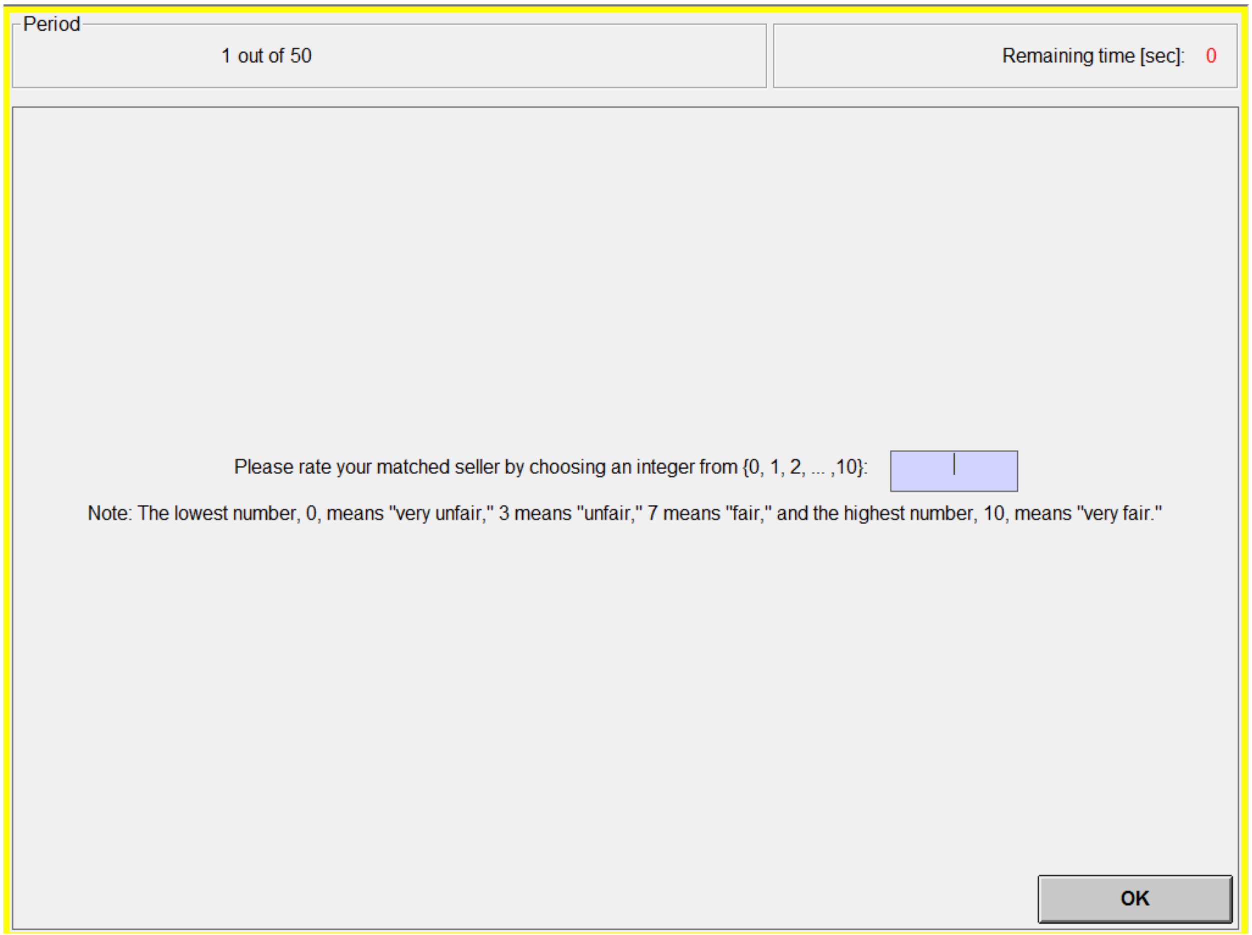

Once all buyers and sellers submit their answers, each seller is informed of the rating given by their matched buyer.

Once everyone in a session has reviewed their feedback, you will move on to the next period, t + 1. In period t +1 as well, each buyer is randomly matched with a seller in your group, and the value of an object is randomly drawn from the set, {0, 1, 2, …, 40}. These random drawing processes are not affected by your period t's decisions or your transaction results.

Once 50 rounds of interactions are completed, the main part of the experiment is over. You will be asked to answer several questions about yourself, such as gender and year in school. Neither information about your decisions during the experiment nor your responses to these questions can be linked to you as an individual.

At the end of the experiment, your accumulated points will be converted to US dollars, and you will privately receive your payment in cash. You will not know about how much others earn in the experiment; nor will the other participants know your payment in the experiment.

Comprehension Question:

Before we begin, please respond to the following questions in order for you to check your understanding. At this time, please raise your hands if you have any questions. When we confirm that every participant has correctly answered the questions, we will start the experiment.

1. How many periods are there in the experiment?

2. Out of the 10 participants in your group, how many of them are assigned the role of "buyer"? Note. As explained, each subject's role, either buyer or seller, does not change during the session.

3. Suppose that you are a "seller," and have been matched with a buyer in period 5. What is the probability that you will be matched with the same buyer in period 6?

4. Suppose that you are assigned the role of "buyer." Your matched seller proposes to sell an object at some price, and you will submit the upper limit of your buying price. Which of the following statement is correct, concerning the value of the object, when you are making the decisions?
   (a) Both you and the seller are informed of the value of the object.

(b) Only the seller is informed of the value of the object.
(c) Only you are informed of the value of the object.
(d) Neither you nor the seller is informed of the value of the object.

5. At the end of each period, each buyer rates his/her matched seller on a 10-point scale. Which of the following statement is correct concerning the rating that a buyer gives?
   (a) Only the matched seller is informed of the rating.
   (b) All participants in the session are informed of the rating.
   (c) Both the matched seller and the other four sellers in his or her group are informed of the rating.
   (d) None in the session is informed of the rating.

Part B: The Instructions Translated into Mandarin

B.1. Instructions of the N-C Treatment

實驗說明

在今天的決策實驗中，你所得到的報酬，將根據你的決策、以及其他參與者的決策而定。在實驗過程中，你的報酬將會以「法幣」來計算。實驗結束時，你所獲得的法幣將會以下列的比率轉換成新台幣：

法幣 1 元＝新台幣 1.4 元

只要你完整的參與了本實驗，你至少會獲得定額車馬費新台幣 100 元。也就是說，你今天的報酬為以下兩者之中較高的金額：新台幣 100 元、或新台幣 100 元加上實驗中的法幣兌換收入。實驗時你的決策是完全匿名的，在實驗結束後，每個人都會個別領取報酬，你沒有義務告訴其他人你的報酬多寡。這個實驗共有 50 回合，共有 20 名參與者。

實驗開始時，電腦會隨機將每 10 位參與者分成一組，實驗中，你只會和自己這小組的參與者互動。每一個小組裡有 5 位買家和 5 位賣家，電腦會隨機指定你是買家還是賣家。也就是說，你被指派到擔任買家或是賣家的機率各是 50%。每一個回合都是一個買家和一個賣家互動。你最初被指派的角色，無論是買家還是賣家，都不會在實驗過程中有所變動。

每一回合開始時，電腦會隨機將一位買家和一位賣家配對，一位買家不會跟超過一位賣家配對。分配過程是完全隨機的：由於每個小組皆有 5 位賣家，因此一位買家跟一位特定賣家配對的機率正好為 1/5。你將會在每一回合做相同形式的決策。

接下來的實驗說明，將詳細地解釋隨機選取的一個回合，第 t 回合。

在第 t 回合，每一位賣家都持有一個相同的物品，這個物品的價值在每回合都不同。

在此回合，物品的價值（q）隨機抽自一組整數，{0, 1, 2, ⋯, 40}。也就是說，每一個數字被抽到的機率是相同的：任一個特定的數字（假設為 12）被選擇當作一個物品的價值的機率為 1/41。你在 t 回合前的選擇、和 t 回合前的每一回合物品價值，皆不會影響此隨機抽取的過程。每一回合中，物品價值被決定後，電腦將會告知買家和賣家真實的物品價值。

每一個賣家被告知物品價值後，會提出物品售價（p）給與他配對的買家。每一位賣家可從{0, 1, ⋯, 40}當中選擇任何一個整數當作物品的售價（p）。在賣家選擇售價的**同時**，買

家從{0, 1, ⋯, 40}中選擇任一整數，當作購買物品的最高願付價格（x）。一旦所有買家和賣家都做好決策後，交易將會開始。

以下的例子是賣家的電腦螢幕畫面：

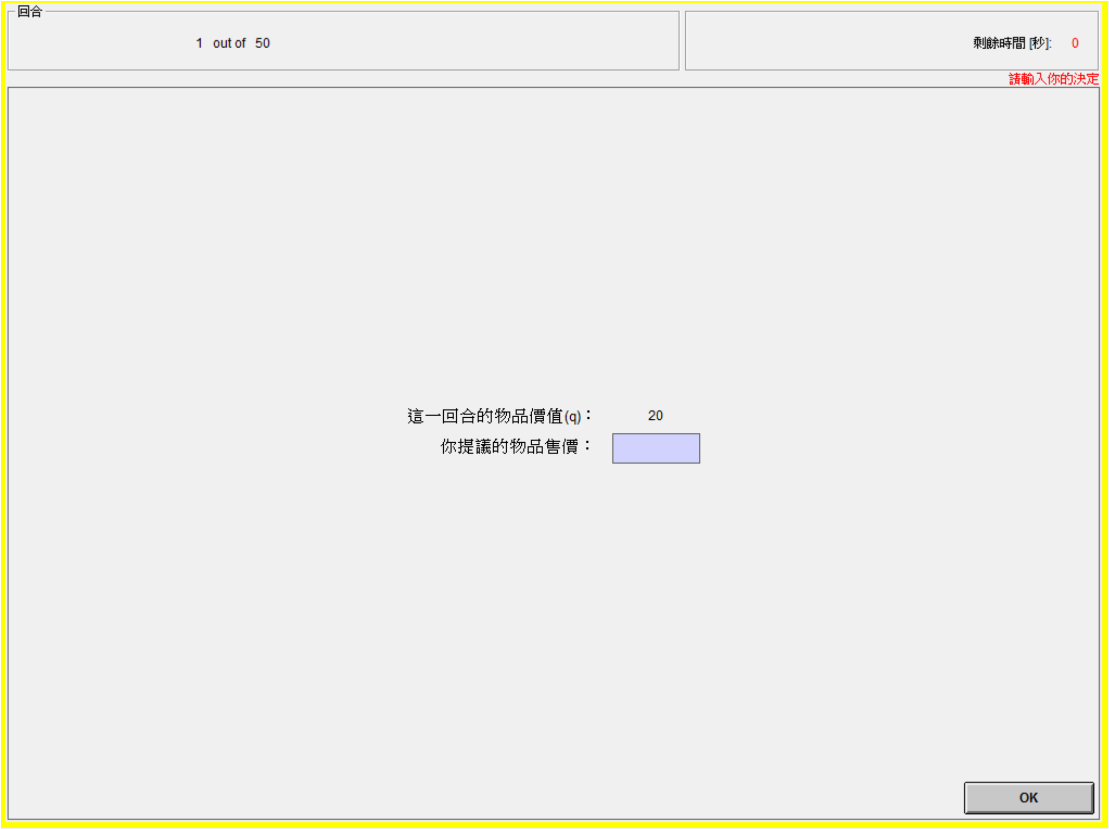

註：本圖數字僅為說明使用。

以下的例子是買家的電腦螢幕畫面：

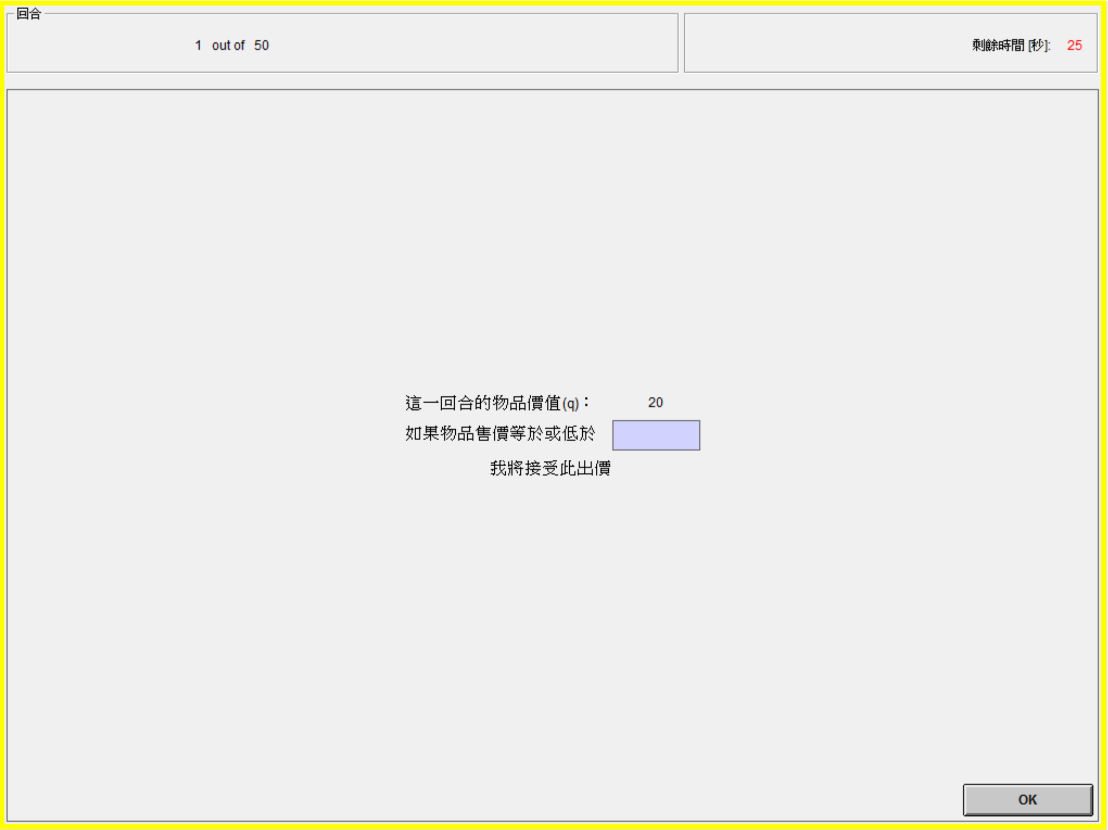

註：本圖數字僅為說明使用。

如果 $p \leq x$，則此交易將會完成，買家得到

$$q - p$$

且賣家得到

$$p - \frac{1}{2} \cdot q$$

你擁有的法幣（報酬）將會隨著回合而累積。如果你某一回合得到的法幣為負值，電腦計算累積法幣時，會從你其他回合所擁有的法幣中扣除。

相反的，如果 $p > x$，則此交易將不會完成，此時買賣雙方皆不會獲得任何法幣(報酬)。

舉一個例子：假設物品的價值為 20，有一位賣家在第 5 回合所提出的物品售價為 14，他/她配對到的買家的最高願付價格為 16。在這個情況下，因為買家的願付價格(16)大於賣家的物品售價(14)，交易完成，買家得到的法幣為 6 (= 20 – 14)，賣家得到的法幣為 4 (= 14 – ½ * 20) 。如果買家的最高願付價格為 5，因為買家的願付價格(5)小於賣家的物品售價(14），在這個情況下，交易不會完成，買賣雙方在這個回合都不會獲得任何的法幣。

每一回合中，當所有買家做出他們的決策後（接受或拒絕），所有人將會知道以下資訊：出價是否有被接受、以及買家和賣家在該回合所獲得的法幣。

當所有人查看完他們的交易結果後，第 t 回合結束，下一個回合（第 $t+1$ 回合）開始。與第 t 回合相同，在第 $t+1$ 回合中，電腦會將每位買家隨機配對到相同小組的其中一位賣家，電腦也會隨機在{0, 1, 2, ⋯, 40}間隨機抽取物品的價值。 這個隨機抽取的過程，不會受到任何參與者在第 t 期做的決策、或第 t 期交易結果的影響。

一旦 50 回合結束，實驗的主要部分即結束。你將會被要求填寫一份問卷，問卷的內容包括了性別、學校年級等。你在實驗中的決策、或你問卷填寫的內容，都不會與你的個人資料相連結。

在實驗結束時，你累積的法幣將會兌換成新台幣，每個人都會個別領取報酬，你沒有義務告訴其他人你的報酬多寡。

理解測驗：

在實驗開始前，請回答下列問題，以確保你對實驗有足夠的了解。作答期間，如果有任何問題，請舉手。在我們確定每一位參與者都能正確的回答這些問題後，我們將開始進行實驗。

1. 這個實驗共有幾個回合？
2. 在你的小組中，包括你共有 10 位參與者，其中有多少人會擔任買家？
   註：如前所述，每一位參與者皆不會在實驗中變動角色（買家或賣家）。
3. 假如你是賣家，在第 5 回合和某買家配對。你在第 6 回合與同一位買家配對到的機率是多少？
4. 假如你是買家。你配對到的賣家提出了一個物品價格，在這個同時，你要提出對物品的最高願付價格。當你在做這個決策時，下列關於物品價值的敘述，哪一項是正確的？

   (a) 你和賣家都知道這個物品的價值。

(b) 只有賣家知道這個物品的價值。
(c) 只有你知道這個物品的價值。
(d) 你和賣家都不會知道這個物品的價值。

B.2. Instructions of the R-C Treatment

實驗說明

在今天的決策實驗中，你所得到的報酬，將根據你的決策、以及其他參與者的決策而定。在實驗過程中，你的報酬將會以「法幣」來計算。實驗結束時，你所獲得的法幣將會以下列的比率轉換成新台幣：

法幣 1 元＝新台幣 1.4 元

只要你完整的參與了本實驗，你至少會獲得定額車馬費新台幣 100 元。也就是說，你今天的報酬為以下兩者之中較高的金額：新台幣 100 元、或新台幣 100 元加上實驗中的法幣兌換收入。實驗時你的決策是完全匿名的，在實驗結束後，每個人都會個別領取報酬，你沒有義務告訴其他人你的報酬多寡。這個實驗共有 50 回合，共有 20 名參與者。

實驗開始時，電腦會隨機將每 10 位參與者分成一組，實驗中，你只會和自己這小組的參與者互動。每一個小組裡有 5 位買家和 5 位賣家，電腦會隨機指定你是買家還是賣家。也就是說，你被指派到擔任買家或是賣家的機率各是 50%。每一個回合都是一個買家和一個賣家互動。你最初被指派的角色，無論是買家還是賣家，都不會在實驗過程中有所變動。

每一回合開始時，電腦會隨機將一位買家和一位賣家配對，一位買家不會跟超過一位賣家配對。分配過程是完全隨機的：由於每個小組皆有 5 位賣家，因此一位買家跟一位特定賣家配對的機率正好為 1/5。你將會在每一回合做相同形式的決策。

接下來的實驗說明，將詳細地解釋隨機選取的一個回合，第 t 回合。

在第 t 回合，每一位賣家都持有一個相同的物品，這個物品的價值在每回合都不同。

在此回合，物品的價值（q）隨機抽自一組整數，{0, 1, 2, …, 40}。也就是說，每一個數字被抽到的機率是相同的：任一個特定的數字（假設為 12）被選擇當作一個物品的價值的機率為 1/41。你在 t 回合前的選擇、和 t 回合前的每一回合物品價值，皆不會影響此隨機抽取的過程。每一回合中，物品價值被決定後，電腦將會告知買家和賣家真實的物品價值。

每一個賣家被告知物品價值後，會提出物品售價（p）給與他配對的買家。每一位賣家可從{0, 1, …, 40}當中選擇任何一個整數當作物品的售價（p）。在賣家選擇售價的**同時**，買家從{0, 1, …, 40}中選擇任一整數，當作購買物品的最高願付價格（x）。一旦所有買家和賣家都做好決策後，交易將會開始。

以下的例子是賣家的電腦螢幕畫面：

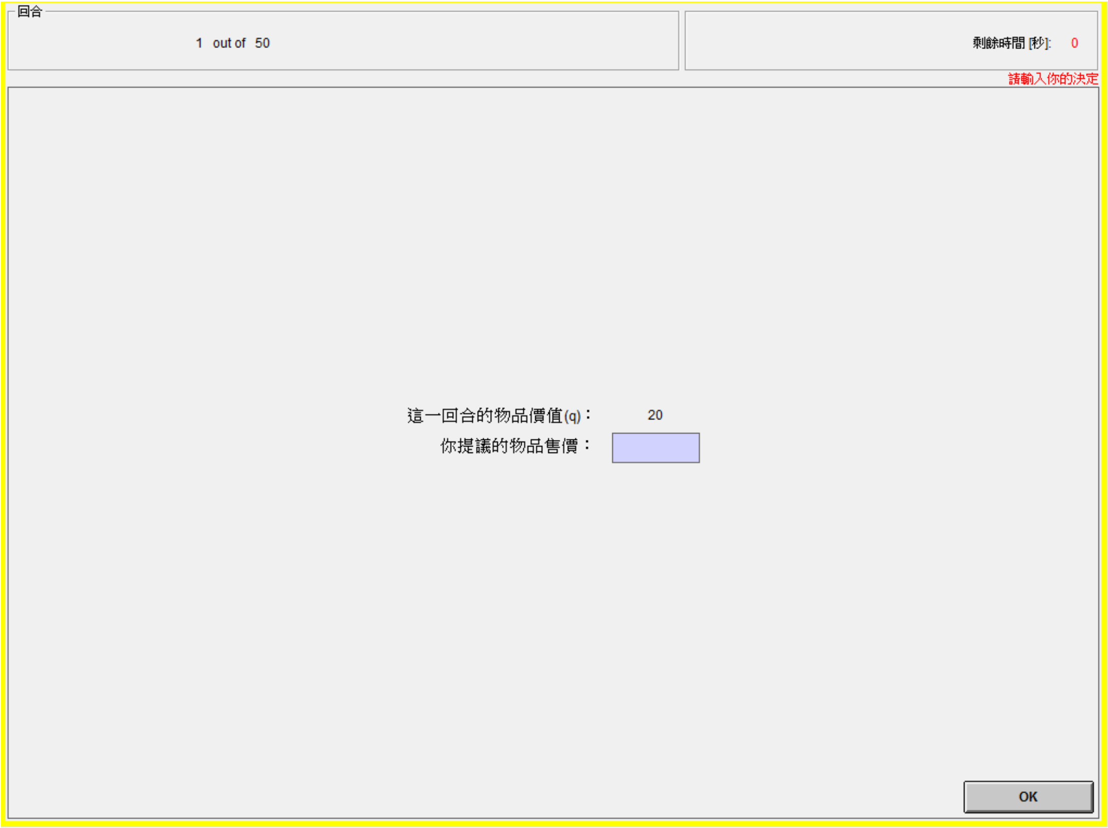

註：本圖數字僅為說明使用。

以下的例子是買家的電腦螢幕畫面：

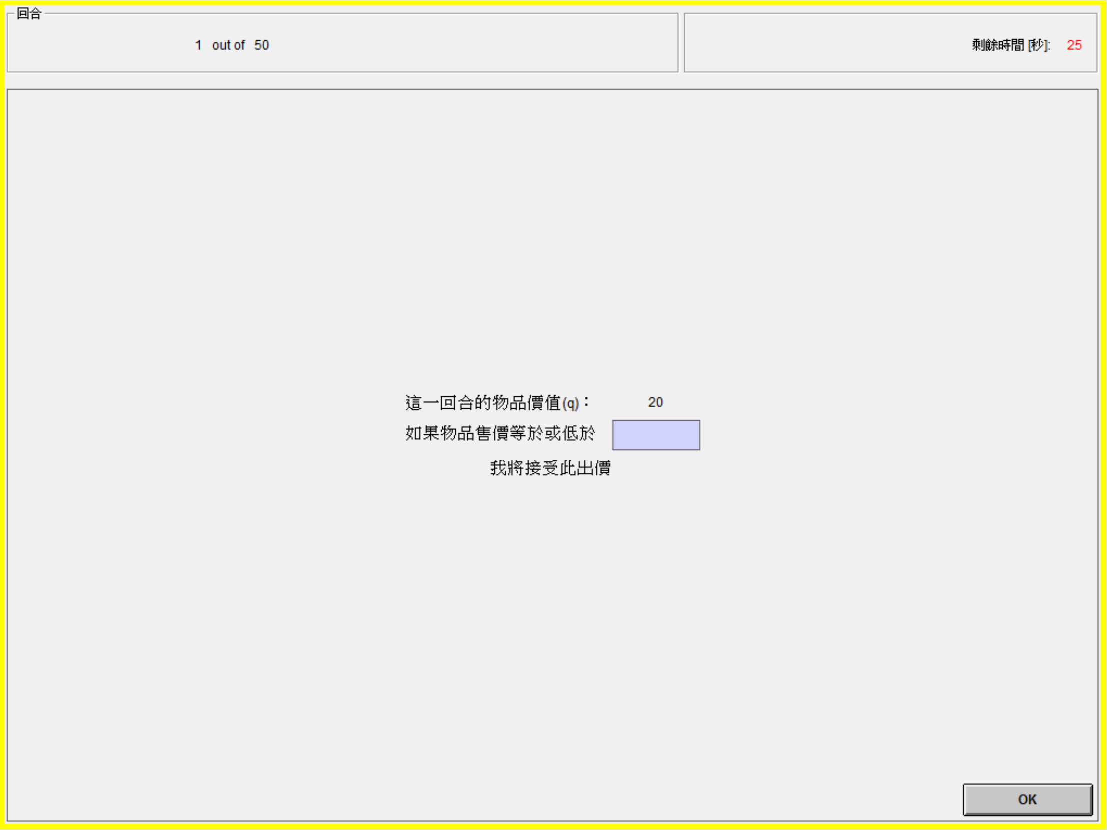

註：本圖數字僅為說明使用。

如果 $p \leq x$，則此交易將會完成，買家得到

$$q - p$$

且賣家得到

$$p - \frac{1}{2} \cdot q$$

你擁有的法幣（報酬）將會隨著回合而累積。如果你某一回合得到的法幣為負值，電腦計算累積法幣時，會從你其他回合所擁有的法幣中扣除。

相反的，如果 $p > x$，則此交易將不會完成，此時買賣雙方皆不會獲得任何法幣(報酬)。

舉一個例子：假設物品的價值為 20，有一位賣家在第 5 回合所提出的物品售價為 14，他/她配對到的買家的最高願付價格為 16。在這個情況下，因為買家的願付價格(16)大於賣家的物品售價(14)，交易完成，買家得到的法幣為 6 (= 20 – 14)，賣家得到的法幣為 4 (= 14 – ½ * 20) 。如果買家的最高願付價格為 5，因為買家的願付價格(5)小於賣家的物品售價(14），在這個情況下，交易不會完成，買賣雙方在這個回合都不會獲得任何的法幣。

每一回合中，當所有買家做出他們的決策後（接受或拒絕），所有人將會知道以下資訊：出價是否有被接受、以及買家和賣家在該回合所獲得的法幣。在這個階段，買家可以從{0, 1, 2, 3, 4, 5, 6, 7, 8, 9, 10}選出一個整數，做為賣家公平與否的評價：最低的數字 0，表示非常不公平，3 表示不公平，7 表示公平，而最高的數字 10，表示非常公平。這些買家所做的評價，不會影響到他/她的報酬，也不會影響到他所配對的賣家的報酬。

在買家給予賣家評價的同時，電腦會要求賣家回答：在之後的回合中，如果你/妳有機會出售與第 t 回合價值相同的物品，你會把物品售價訂在多少？賣家的這個回應是假設性的，不會影響買賣雙方在第 t 回合得到的法幣，也不會影響到之後他們所做的決策。

以下的例子是買家的電腦螢幕畫面：

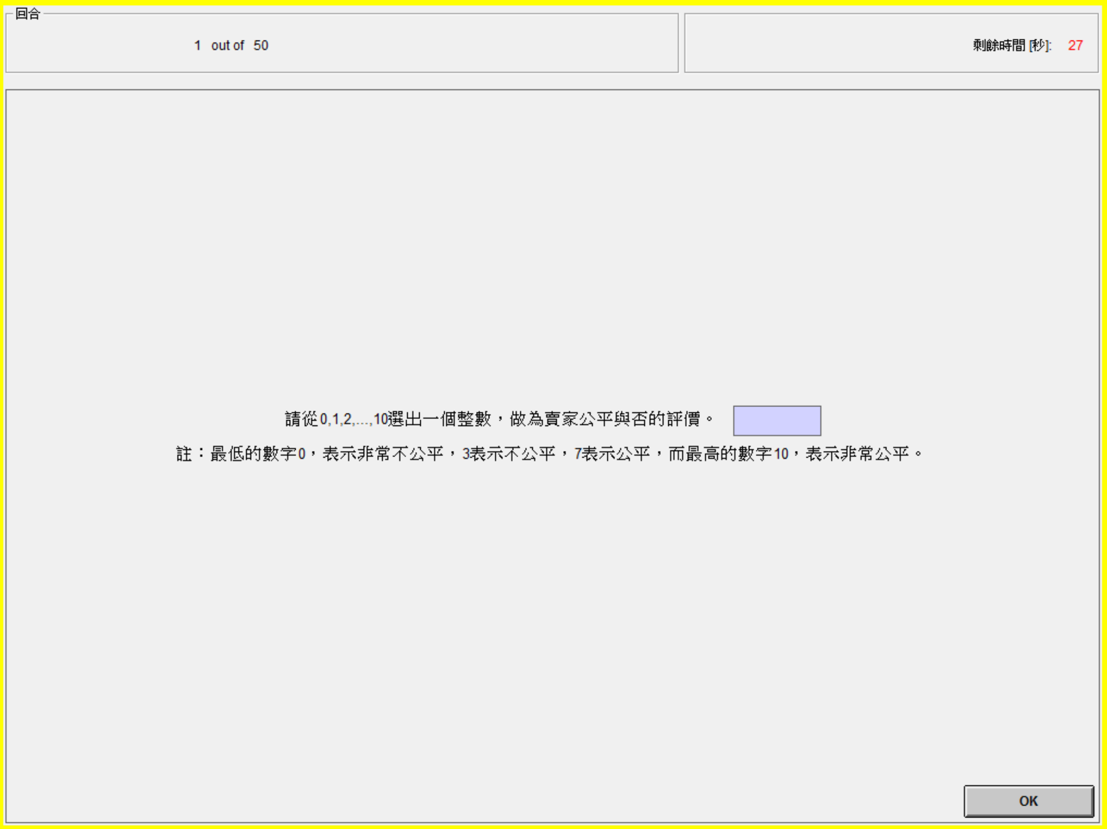

一旦所有買家和賣家提交出他們的答案後，電腦會告知每位賣家，他所配對的買家給他們的評價。

當所有人查看完他們的評價後，第 t 回合結束，下一個回合（第 $t+1$ 回合）開始。與第 t 回合相同，在第 $t+1$ 回合中，電腦會將每位買家隨機配對到相同小組的其中一位賣家，電腦也會隨機在{0, 1, 2, ⋯, 40}間隨機抽取物品的價值。 這個隨機抽取的過程，不會受到任何參與者在第 t 期做的決策、或第 t 期交易結果的影響。

一旦 50 回合結束，實驗的主要部分即結束。你將會被要求填寫一份問卷，問卷的內容包括了性別、學校年級等。你在實驗中的決策、或你問卷填寫的內容，都不會與你的個人資料相連結。

在實驗結束時，你累積的法幣將會兌換成新台幣，每個人都會個別領取報酬，你沒有義務告訴其他人你的報酬多寡。

理解測驗：

在實驗開始前，請回答下列問題，以確保你對實驗有足夠的了解。作答期間，如果有任何問題，請舉手。在我們確定每一位參與者都能正確的回答這些問題後，我們將開始進行實驗。

1. 這個實驗共有幾個回合？
2. 在你的小組中，包括你共有 10 位參與者，其中有多少人會擔任買家？
   註：如前所述，每一位參與者皆不會在實驗中變動角色（買家或賣家）。
3. 假如你是賣家，在第 5 回合和某買家配對。你在第 6 回合與同一位買家配對到的機率是多少？
4. 假如你是買家。你配對到的賣家提出了一個物品價格，在這個同時，你要提出對物品的最高願付價格。當你在做這個決策時，下列關於物品價值的敘述，哪一項是正確的？
   (a)你和賣家都知道這個物品的價值。
   (b) 只有賣家知道這個物品的價值。
   (c) 只有你知道這個物品的價值。
   (d) 你和賣家都不會知道這個物品的價值
5. 每一回合結束時，每一位買家會去評價他配對的賣家，評價以 10 分為滿分。下列關於買家給予評價的敘述，哪一項是正確的？
   (a) 只有與買家配對的賣家會知道他自己的評價。
   (b) 所有實驗參與者都會知道這個賣家的評價。
   (c) 與買家配對的賣家、和小組裡的其他 4 位賣家，都會被知道這個賣家的評價。
   (d) 沒有人會知道這個評價。

B.3. Instructions of the N-IC Treatment

實驗說明

在今天的決策實驗中，你所得到的報酬，將根據你的決策、以及其他參與者的決策而定。在實驗過程中，你的報酬將會以「法幣」來計算。實驗結束時，你所獲得的法幣將會以下列的比率轉換成新台幣：

法幣 1 元＝新台幣 1.4 元

只要你完整的參與了本實驗，你至少會獲得定額車馬費新台幣 100 元。也就是說，你今天的報酬為以下兩者之中較高的金額：新台幣 100 元、或新台幣 100 元加上實驗中的法幣兌換收入。實驗時你的決策是完全匿名的，在實驗結束後，每個人都會個別領取報酬，你沒有義務告訴其他人你的報酬多寡。

這個實驗共有 50 回合，共有 20 名參與者。

實驗開始時，電腦會隨機將每 10 位參與者分成一組，實驗中，你只會和自己這小組的參與者互動。每一個小組裡有 5 位買家和 5 位賣家，電腦會隨機指定你是買家還是賣家。也就是說，你被指派到擔任買家或是賣家的機率各是 50%。每一個回合都是一個買家和一個賣家互動。你最初被指派的角色，無論是買家還是賣家，都不會在實驗過程中有所變動。

每一回合開始時，電腦會隨機將一位買家和一位賣家配對，一位買家不會跟超過一位賣家配對。分配過程是完全隨機的：由於每個小組皆有 5 位賣家，因此一位買家跟一位特定賣家配對的機率正好為 1/5。你將會在每一回合做相同形式的決策。

接下來的實驗說明，將詳細地解釋隨機選取的一個回合，第 t 回合。

在第 t 回合，每一位賣家都持有一個相同的物品，這個物品的價值在每回合都不同。

在此回合，物品的價值（q）隨機抽自一組整數，{0, 1, 2, ⋯, 40}。也就是說，每一個數字被抽到的機率是相同的：任一個特定的數字（假設為 12）被選擇當作一個物品的價值的機率為 1/41。你在 t 回合前的選擇、和 t 回合前的每一回合物品價值，皆不會影響此隨機抽取的過程。每一回合中，物品價值被決定後，電腦將會告知賣家真實的物品價值，但不會告知買家。

每一個賣家被告知物品價值後，會提出物品售價（p）給與他配對的買家。每一位賣家可從{0, 1, ⋯, 40}當中選擇任何一個整數當作物品的售價（p）。在賣家選擇售價的**同時**，買家從{0, 1, ⋯, 40}中選擇任一整數，當作購買物品的最高願付價格（x）。一旦所有買家和賣家都做好決策後，交易將會開始。

以下的例子是賣家的電腦螢幕畫面：

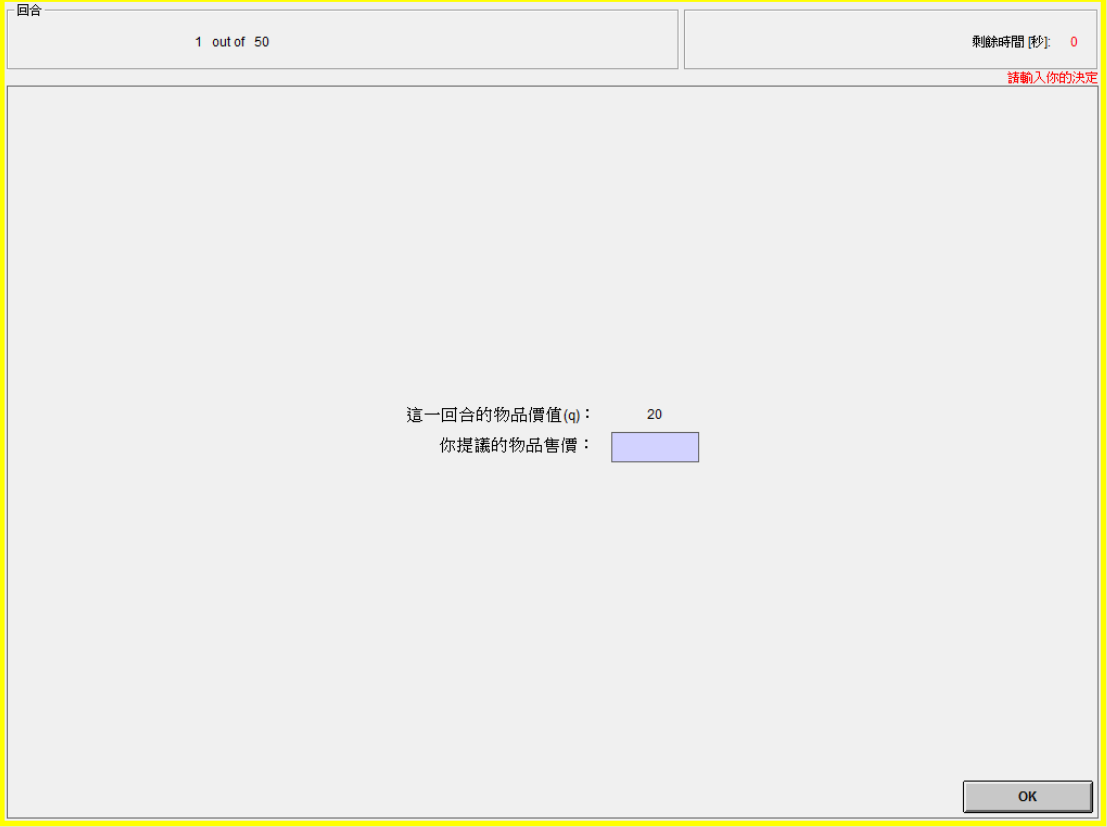

註：本圖數字僅為說明使用。

以下的例子是買家的電腦螢幕畫面：

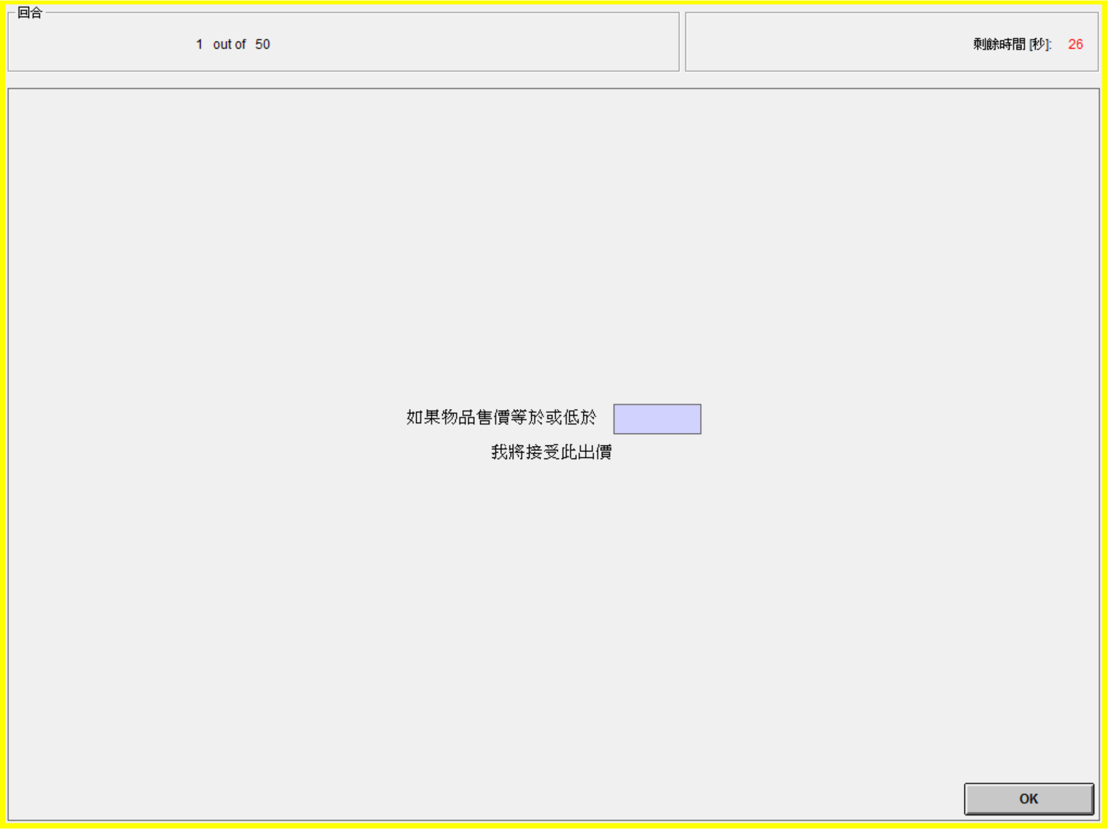

如果 $p \leq x$，則此交易將會完成，買家得到

$$q - p$$

且賣家得到

$$p - \frac{1}{2} \cdot q$$

你擁有的法幣（報酬）將會隨著回合而累積。如果你某一回合得到的法幣為負值，電腦計算累積法幣時，會從你其他回合所擁有的法幣中扣除。

相反的，如果 $p > x$，則此交易將不會完成，此時買賣雙方皆不會獲得任何法幣(報酬)。

舉一個例子：假設物品的價值為 20，有一位賣家在第 5 回合所提出的物品售價為 14，他/她配對到的買家的最高願付價格為 16。在這個情況下，因為買家的願付價格(16)大於賣家

的物品售價(14)，交易完成，買家得到的法幣為 6 (= 20 － 14)，賣家得到的法幣為 4 (= 14 － ½ * 20) 。如果買家的最高願付價格為 5，因為買家的願付價格(5)小於賣家的物品售價(14)，在這個情況下，交易不會完成，買賣雙方在這個回合都不會獲得任何的法幣。

每一回合中，當所有買家做出他們的決策後（接受或拒絕），所有人將會知道以下資訊：物品的價值、出價是否有被對方接受、以及買家和賣家在該回合所獲得的法幣。

當所有人查看完他們的交易結果後，第 t 回合結束，下一個回合（第 $t+1$ 回合）開始。與第 t 回合相同，在第 $t+1$ 回合中，電腦會將每位買家隨機配對到相同小組的其中一位賣家，電腦也會隨機在{0, 1, 2, ⋯, 40}間隨機抽取物品的價值。 這個隨機抽取的過程，不會受到任何參與者在第 t 期做的決策、或第 t 期交易結果的影響。

一旦 50 回合結束，實驗的主要部分即結束。你將會被要求填寫一份問卷，問卷的內容包括了性別、學校年級等。你在實驗中的決策、或你問卷填寫的內容，都不會與你的個人資料相連結。

在實驗結束時，你累積的法幣將會兌換成新台幣，每個人都會個別領取報酬，你沒有義務告訴其他人你的報酬多寡。

理解測驗：

在實驗開始前，請回答下列問題，以確保你對實驗有足夠的了解。作答期間，如果有任何問題，請舉手。在我們確定每一位參與者都能正確的回答這些問題後，我們將開始進行實驗。

1. 這個實驗共有幾個回合？
2. 在你的小組中，包括你共有 10 位參與者，其中有多少人會擔任買家？
   註：如前所述，每一位參與者皆不會在實驗中變動角色（買家或賣家）。
3. 假如你是賣家，在第 5 回合和某買家配對。你在第 6 回合與同一位買家配對到的機率是多少？
4. 假如你是買家。你配對到的賣家提出了一個物品價格，在這個同時，你要提出對物品的最高願付價格。當你在做這個決策時，下列關於物品價值的敘述，哪一項是正確的？

   (a) 你和賣家都知道這個物品的價值。
   (b) 只有賣家知道這個物品的價值。
   (c) 只有你知道這個物品的價值。
   (d) 你和賣家都不會知道這個物品的價值。

B.4. Instructions of the R-IC Treatment

實驗說明

在今天的決策實驗中，你所得到的報酬，將根據你的決策、以及其他參與者的決策而定。在實驗過程中，你的報酬將會以「法幣」來計算。實驗結束時，你所獲得的法幣將會以下列的比率轉換成新台幣：

法幣 1 元＝新台幣 1.4 元

只要你完整的參與了本實驗，你至少會獲得定額車馬費新台幣 100 元。也就是說，你今天的報酬為以下兩者之中較高的金額：新台幣 100 元、或新台幣 100 元加上實驗中的法幣兌換收入。實驗時你的決策是完全匿名的，在實驗結束後，每個人都會個別領取報酬，你沒有義務告訴其他人你的報酬多寡。這個實驗共有 50 回合，共有 20 名參與者。

實驗開始時，電腦會隨機將每 10 位參與者分成一組，實驗中，你只會和自己這小組的參與者互動。每一個小組裡有 5 位買家和 5 位賣家，電腦會隨機指定你是買家還是賣家。也就是說，你被指派到擔任買家或是賣家的機率各是 50%。每一個回合都是一個買家和一個賣家互動。你最初被指派的角色，無論是買家還是賣家，都不會在實驗過程中有所變動。

每一回合開始時，電腦會隨機將一位買家和一位賣家配對，一位買家不會跟超過一位賣家配對。分配過程是完全隨機的：由於每個小組皆有 5 位賣家，因此一位買家跟一位特定賣家配對的機率正好為 1/5。你將會在每一回合做相同形式的決策。

接下來的實驗說明，將詳細地解釋隨機選取的一個回合，第 t 回合。

在第 t 回合，每一位賣家都持有一個相同的物品，這個物品的價值在每回合都不同。

在此回合，物品的價值（q）隨機抽自一組整數，{0, 1, 2, ⋯, 40}。也就是說，每一個數字被抽到的機率是相同的：任一個特定的數字（假設為 12）被選擇當作一個物品的價值的機率為 1/41。你在 t 回合前的選擇、和 t 回合前的每一回合物品價值，皆不會影響此隨機抽取的過程。每一回合中，物品價值被決定後，電腦將會告知賣家真實的物品價值，但不會告知買家。

每一個賣家被告知物品價值後，會提出物品售價（p）給與他配對的買家。每一位賣家可從{0, 1, ⋯, 40}當中選擇任何一個整數當作物品的售價（p）。在賣家選擇售價的**同時**，買家從{0, 1, ⋯, 40}中選擇任一整數，當作購買物品的最高願付價格（x）。一旦所有買家和賣家都做好決策後，交易將會開始。

以下的例子是賣家的電腦螢幕畫面：

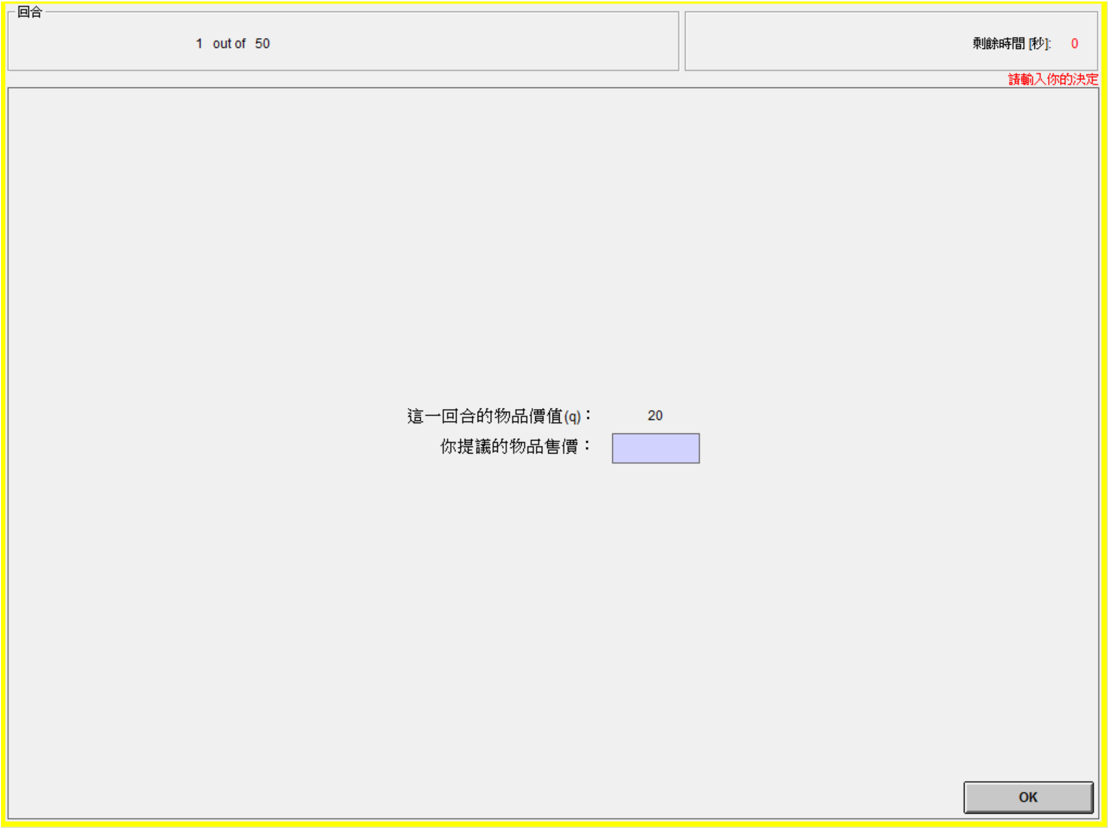

註：本圖數字僅為說明使用。

以下的例子是買家的電腦螢幕畫面：

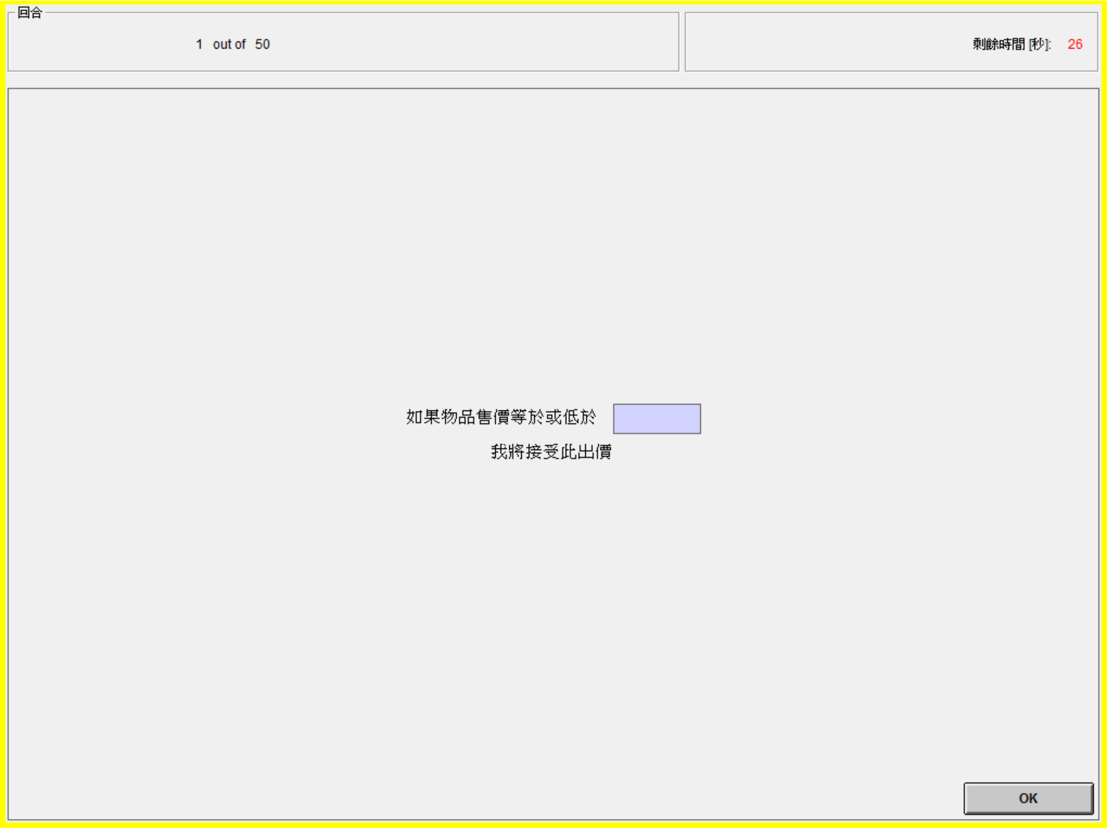

如果 $p \leq x$，則此交易將會完成，買家得到

$$q - p$$

且賣家得到

$$p - \frac{1}{2} \cdot q$$

你擁有的法幣（報酬）將會隨著回合而累積。如果你某一回合得到的法幣為負值，電腦計算累積法幣時，會從你其他回合所擁有的法幣中扣除。

相反的，如果 $p > x$，則此交易將不會完成，此時買賣雙方皆不會獲得任何法幣(報酬)。

舉一個例子：假設物品的價值為 20，有一位賣家在第 5 回合所提出的物品售價為 14，他/她配對到的買家的最高願付價格為 16。在這個情況下，因為買家的願付價格(16)大於賣家

的物品售價(14)，交易完成，買家得到的法幣為 6 (= 20 – 14)，賣家得到的法幣為 4 (= 14 – ½ * 20) 。如果買家的最高願付價格為 5，因為買家的願付價格(5)小於賣家的物品售價(14），在這個情況下，交易不會完成，買賣雙方在這個回合都不會獲得任何的法幣。

每一回合中，當所有買家做出他們的決策後（接受或拒絕），所有人將會知道以下資訊：物品的價值、出價是否有被對方接受、以及買家和賣家在該回合所獲得的法幣。在這個階段，買家可以從{0, 1, 2, 3, 4, 5, 6, 7, 8, 9, 10}選出一個整數，做為賣家公平與否的評價：最低的數字 0，表示非常不公平，3 表示不公平，7 表示公平，而最高的數字 10，表示非常公平。這些買家所做的評價，不會影響到他/她的報酬，也不會影響到他所配對的賣家的報酬。

在買家給予賣家評價的同時，電腦會要求賣家回答：在之後的回合中，如果你/妳有機會出售與第 t 回合價值相同的物品，你會把物品售價訂在多少？賣家的這個回應是假設性的，不會影響買賣雙方在第 t 回合得到的法幣，也不會影響到之後他們所做的決策。

以下的例子是買家的電腦螢幕畫面：

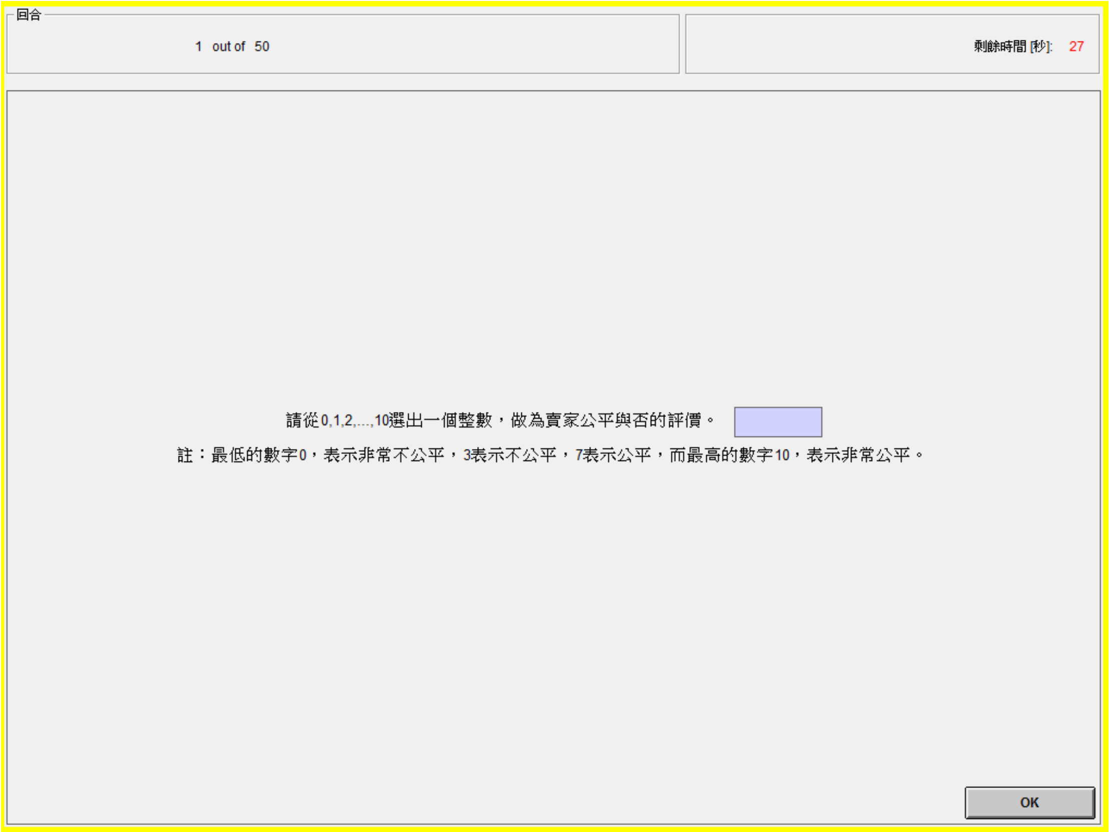

一旦所有買家和賣家提交出他們的答案後，電腦會告知每位賣家，他所配對的買家給他們的評價。

當所有人查看完他們的評價後，第 t 回合結束，下一個回合（第 $t+1$ 回合）開始。與第 t 回合相同，在第 $t+1$ 回合中，電腦會將每位買家隨機配對到相同小組的其中一位賣家，電腦也會隨機在{0, 1, 2, ···, 40}間隨機抽取物品的價值。 這個隨機抽取的過程，不會受到任何參與者在第 t 期做的決策、或第 t 期交易結果的影響。

一旦 50 回合結束，實驗的主要部分即結束。你將會被要求填寫一份問卷，問卷的內容包括了性別、學校年級等。你在實驗中的決策、或你問卷填寫的內容，都不會與你的個人資料相連結。

在實驗結束時，你累積的法幣將會兌換成新台幣，每個人都會個別領取報酬，你沒有義務告訴其他人你的報酬多寡。

理解測驗：

在實驗開始前，請回答下列問題，以確保你對實驗有足夠的了解。作答期間，如果有任何問題，請舉手。在我們確定每一位參與者都能正確的回答這些問題後，我們將開始進行實驗。

1. 這個實驗共有幾個回合？
2. 在你的小組中，包括你共有 10 位參與者，其中有多少人會擔任買家？
   註：如前所述，每一位參與者皆不會在實驗中變動角色（買家或賣家）。
3. 假如你是賣家，在第 5 回合和某買家配對。你在第 6 回合與同一位買家配對到的機率是多少？
4. 假如你是買家。你配對到的賣家提出了一個物品價格，在這個同時，你要提出對物品的最高願付價格。當你在做這個決策時，下列關於物品價值的敘述，哪一項是正確的？

   (e) 你和賣家都知道這個物品的價值。
   (f) 只有賣家知道這個物品的價值。
   (g) 只有你知道這個物品的價值。
   (h) 你和賣家都不會知道這個物品的價值

5. 每一回合結束時，每一位買家會去評價他配對的賣家，評價以 10 分為滿分。下列關於買家給予評價的敘述，哪一項是正確的？

   (a) 只有與買家配對的賣家會知道他自己的評價。
   (b) 所有實驗參與者都會知道這個賣家的評價。

(c) 與買家配對的賣家、和小組裡的其他 4 位賣家，都會被知道這個賣家的評價。
(d) 沒有人會知道這個評價。
