# Supplementary material for: Disapproval aversion or inflated inequity acceptance? The impact of expressing emotions in ultimatum bargaining
Source: Exp Econ. 2017 Nov 13;21(4):836–57. doi: 10.1007/s10683-017-9554-z (PMC6223739; doi:10.1007/s10683-017-9554-z)
Supplement: Supplementary file 2 — Supplementary material 2 (PDF 641 kb) [file 10683_2017_9554_MOESM2_ESM.pdf]

Electronic Supplementary Material for Chen and Kamei, 2017,

"Disapproval Aversion or Inflated Inequity Acceptance? The Impact of Expressing Emotions in Ultimatum Bargaining"[1]

This Appendix contains theoretical analyses and additional tables that supplement Chen and Kamei (2017).

Table of Contents

Appendix A: Theoretical Analysis . . . . p. 2

Appendix B: Additional Tables . . . . p. 16

[1] Correspondence: Kenju Kamei, Department of Economics and Finance, Durham University, Mill Hill Lane, Durham, DH1 3LB, United Kingdom. Email: kenju.kamei@gmail.com, kenju.kamei@durham.ac.uk.

Appendix A: Theoretical Analysis

A.1. *Standard Theory Predictions for the N-C and R-C Treatments*

For an assigned $q \in [0, 40]$, a seller maximizes his payoff with respect to the price p_s, given the paired buyer's purchase threshold p_b,:

$$max_{p_s}\left\{\left(p_s - \frac{1}{2}q\right) \cdot Prob\{p_s \leq p_b\}\right\}.$$

Likewise, the buyer maximizes her payoff with respect to the purchase threshold p_b, given the paired seller's offering price p_s:

$$max_{p_b}\{(q - p_s) \cdot Prob\{p_s \leq p_b\}\}.$$

Thus, the best responses of the seller and buyer are calculated as below:

Seller: $p_s = p_b$ if $p_b - \frac{1}{2}q \geq 0$; $p_s = \xi$ such that $\xi > p_b$ if $p_b - \frac{1}{2}q < 0$.

Buyer: $p_b = p_s$ if $q - p_s \geq 0$; $p_b = \nu$ such that $\nu < p_s$ if $q - p_s < 0$.

These best response correspondences are depicted as below:

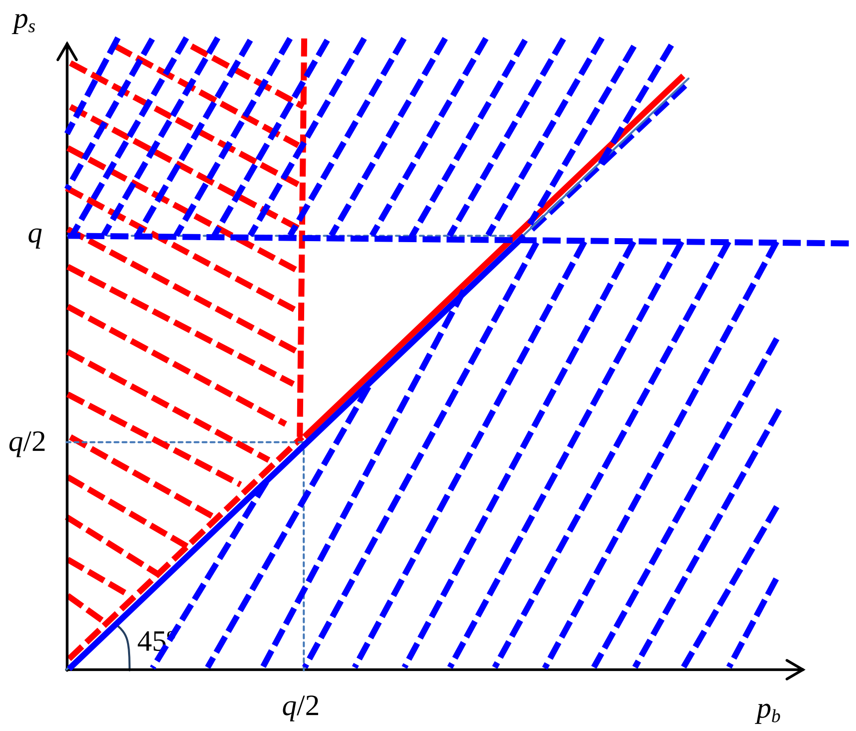

Figure A.1: The Best Response Strategies of the Seller and Buyer

In Figure A.1, the red line and the red dash region indicate the best responses of the seller, whereas the blue line and the blue dash region indicate the best responses of the buyer. From this, the set of Nash equilibria where trades between the seller and the buyer are closed is summarized as below:

$$\left\{(p_b, p_s) | p_s = p_b = x \in \left[\frac{1}{2}q, q\right]\right\}.$$

In each equilibrium, the payoff for the seller (π_s) is $x - q/2$ and that for the buyer (π_b) is $q - x$. The set of Nash equilibria where trades between the seller and the buyer are not closed is summarized as below:

$$\left\{(p_b, p_s) | p_s > q \text{ and } p_b < \frac{1}{2}q\right\}.$$

A.2. *Inequality-Averse Preferences and Best Response Strategies for the N-C and R-C Treatments*

Suppose that a seller is inequality-averse as defined in the paper: $u_{sj}(\pi_{sj}, \pi_{bi}) = \pi_{sj} - \mu_j \cdot (\pi_{sj} - \pi_{bi})^2$, where $\pi_{bi} = (q - p_{sj}) \cdot 1_{\{p_{sj} \le p_{bi}\}}$ and $\pi_{sj} = \left(p_{sj} - \frac{1}{2}q\right) \cdot 1_{\{p_{sj} \le p_{bi}\}}$. Here, $1_{\{p_{sj} \le p_{bi}\}} = 1$ when $p_{sj} \le p_{bi}$; = 0 otherwise. Then, given the matched buyer's strategy, p_{bi}, for each $q \in [0,40]$, seller *j* maximizes the following payoff with respect to p_{sj}:

$$\left\{\pi_{sj} - \mu_j \cdot (\pi_{sj} - \pi_{bi})^2\right\} \cdot 1_{\{p_{sj} \le p_{bi}\}}$$

$$= \left\{p_{sj} - \frac{1}{2}q - \mu_j \cdot \left(2p_{sj} - \frac{3}{2}q\right)^2\right\} \cdot 1_{\{p_{sj} \le p_{bi}\}}, \qquad \text{(A1)}$$

The term within the first curly bracket is maximized at: $p_{sj} = \frac{1}{8\mu_j} + \frac{3}{4}q$, as the derivative of it with respect to p_{sj} is: $1 + 6\mu_j \cdot q - 8\mu_j \cdot p_{sj}$. The value in the first curly bracket at $p_{sj} = \frac{1}{8\mu_j} + \frac{3}{4}q$ reduces to:

$$\frac{1}{4}q + \frac{1}{16\mu_j}.$$

Thus, given p_{bi}, if *q* is small enough that $q \le \frac{4}{3}p_{bi} - \frac{1}{6\mu_j}$ so that $p_{sj} \le p_{bi}$, the seller's best response function is given by: $p_{sj} = \frac{1}{8\mu_j} + \frac{3}{4}q$. In contrast, if *q* is large enough that $q > \frac{4}{3}p_{bi} - \frac{1}{6\mu_j}$, then $p_{sj} = p_{bi}$ is the seller's best response function if the value in the curly bracket is still positive

at $p_{sj} = p_{bi}$: $p_{bi} - \frac{1}{2}q - \mu_j \cdot \left(2p_{bi} - \frac{3}{2}q\right)^2 > 0$; otherwise, $p_{sj} > p_{bi}$ becomes his best response. In short, the seller's best response function is summarized as:

$$p_{sj} = \begin{cases} \frac{3}{4}q + \frac{1}{8\mu_j}, & \text{if } q \leq \frac{4}{3}p_{bi} - \frac{1}{6\mu_j}. \\ p_{bi}, & \text{if } q > \frac{4}{3}p_{bi} - \frac{1}{6\mu_j} \text{ and } p_{bi} - \frac{1}{2}q - \mu_j \cdot \left(2p_{bi} - \frac{3}{2}q\right)^2 > 0. \\ \text{any } c, \text{s.t. } c > p_{bi}, & \text{if } q > \frac{4}{3}p_{bi} - \frac{1}{6\mu_j} \text{ and } p_{bi} - \frac{1}{2}q - \mu_j \cdot \left(2p_{bi} - \frac{3}{2}q\right)^2 < 0. \end{cases} \quad \text{(A2)}$$

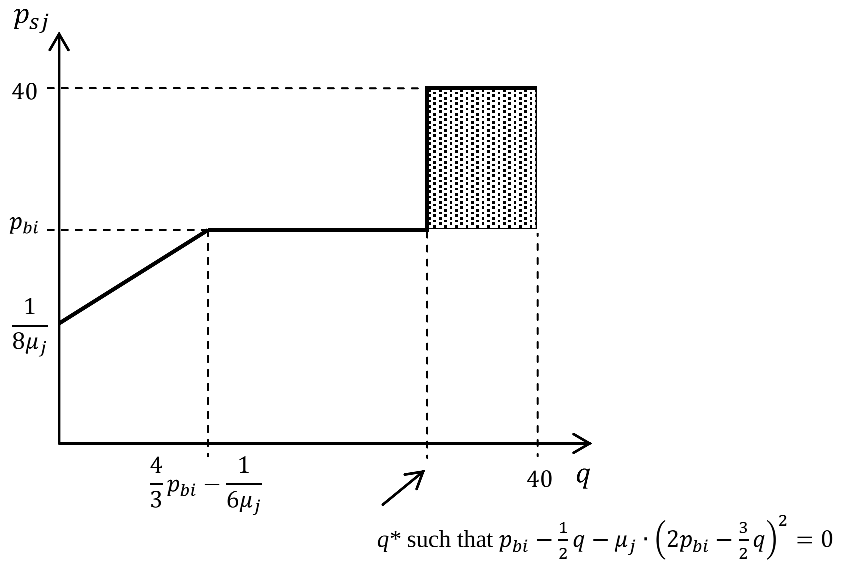

Figure A.2: The Best Response Strategy of the Seller

Here, the acceptance rate of the offering prices in the experiment would be $q^*/40$ in expectation as q is randomly drawn from the uniform distribution between 0 and 40. Note that the intercept in Figure A.2 (the seller's best response price at q = 0) is $\frac{1}{8\mu_j}$. This is not dependent on p_{bi}. If $p_{bi} < \frac{1}{8\mu_j}$, then the seller's best response strategy becomes as follows:

$$p_{sj} = \begin{cases} p_{bi}, & \text{if } p_{bi} - \frac{1}{2}q - \mu_j \cdot \left(2p_{bi} - \frac{3}{2}q\right)^2 > 0 \\ \text{any } c, \text{s.t. } c > p_{bi}, & \text{if } p_{bi} - \frac{1}{2}q - \mu_j \cdot \left(2p_{bi} - \frac{3}{2}q\right)^2 < 0. \end{cases} \quad \text{(A3)}$$

For simplicity, we assume that $p_{bi} > \frac{1}{8\mu_j}$ in the rest of this Appendix A.

Also, note that $\frac{\partial q^*}{\partial p_{bi}} > 0$ since q^* is a point at which $y = p_{bi} - \frac{1}{2}q$ and $y = \mu_j \cdot \left(2p_{bi} - \frac{3}{2}q\right)^2$ intersect; both curves shift to the right when p_{bi} increases as shown in the following figure.[2]

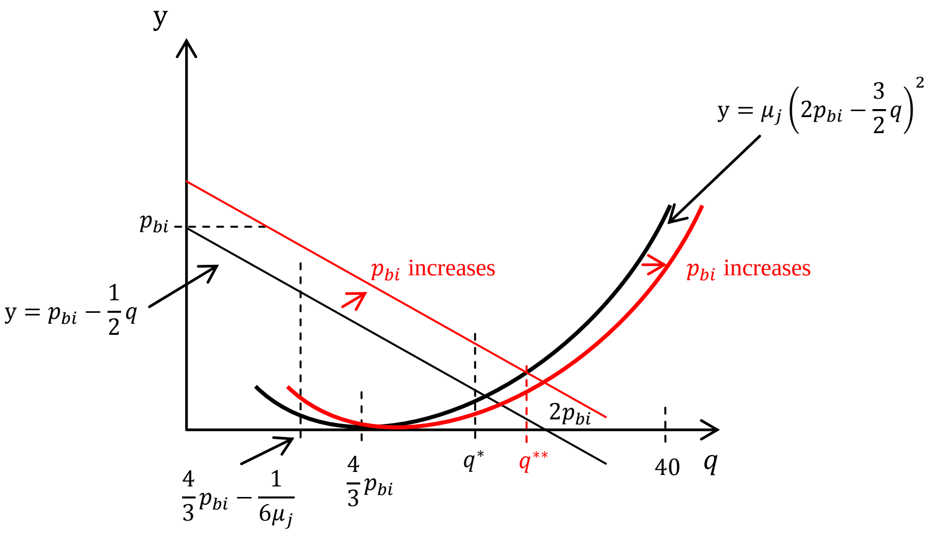

Thus, we find that the seller's best response strategies shift as below responding to a chance in p_{bi}.

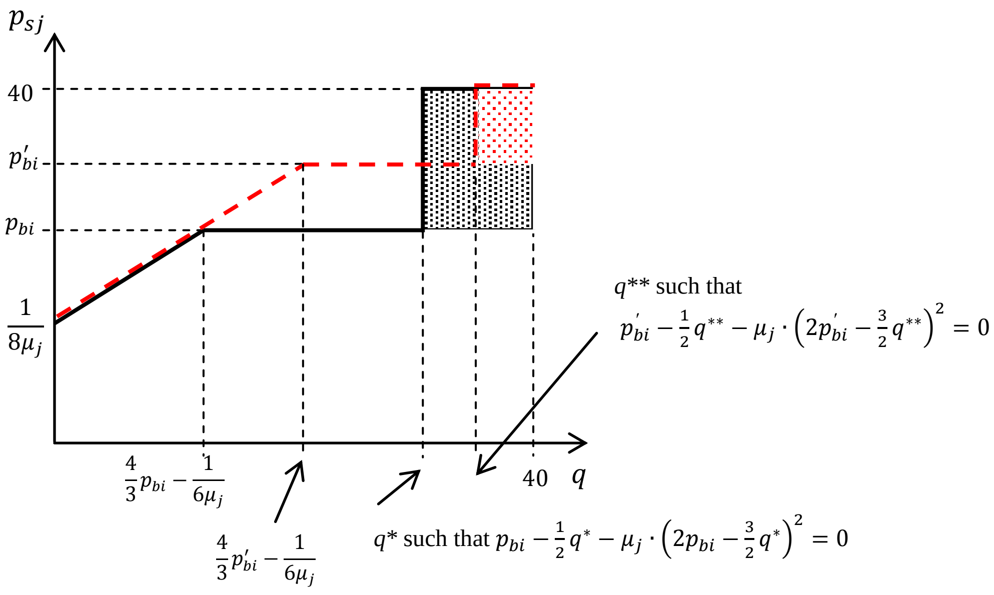

Figure A.3: The Seller's Best Response Strategies for Various p_{bi}

Note: The solid (dashed) line indicates the best response strategy of seller *j* when faced with p_{bi} (p'_{bi}).

---

[2] q^* can be greater than 40. In that case, the seller's best response price is less than or equal to p_{bi} for any value $q \in [0,40]$.

Suppose also that the buyer is inequality-averse, as assumed in the paper: $u_{bi}(\pi_{bi}, \pi_{sj}) = \pi_{bi} - \mu_i \cdot (\pi_{sj} - \pi_{bi})^2$. Notice here that the utility weight on inequality μ_i is different from that of seller μ_j. The buyer maximizes her utility, given p_{sj}:

$$u_{bi}(\pi_{bi}, \pi_{sj}) = \left[q - p_{sj} - \mu_i \cdot \left(2p_{sj} - \tfrac{3}{2}q\right)^2\right] \cdot 1_{\{p_{sj} \leq p_{bi}\}}.$$

This means that the buyer's best response strategy is: $p_{bi} \geq p_{sj}$ when $u_{bi} = q - p_{sj} - \mu_i \cdot \left(2p_{sj} - \tfrac{3}{2}q\right)^2 \geq 0$, but $p_{bi} = \xi$, such that $\xi < p_{sj}$ if $q - p_{sj} - \mu_i \cdot \left(2p_{sj} - \tfrac{3}{2}q\right)^2 < 0$. For a given q, the best response correspondences of the buyer and those of the seller described in Conditions (A2) and (A3) characterize the set of Nash equilibria.

A.3. *Standard Theory Predictions for the N-IC and R-IC Treatments*

For each $q \in [0, 40]$, given the buyer's purchase threshold p_b, the seller maximizes his payoff with respect to the price p_s:

$$max_{p_s}\left\{\left(p_s - \tfrac{1}{2}q\right) \cdot Prob\{p_s \leq p_b\}\right\}.$$

We obtain, from this maximization problem, the best response function of the seller as follows:

$$b_s(q) = \begin{cases} p_b & \text{for } 2p_b \geq q. \\ \tilde{p}\ s.t.\ \tilde{p} > p_b & \text{for } 2p_b < q. \end{cases} \quad \text{(A4)}$$

Likewise, given the seller's strategy $p_s(q)$, the buyer maximizes her expected payoff with respect to p_b as the value of the commodity is unknown to her. This reduces to the following maximization problem:

$$max_{p_b}\left\{\pi_b = \int_0^{40} (q - p_s(q)) \cdot 1_{\{p_s(q) \leq p_b\}} \cdot \tfrac{1}{40}dq\right\}. \quad \text{(A5)}$$

There exist Bayesian Nash equilibria characterized by the seller's best response specified in condition (A4) and by the following best response strategy of the buyer:

$$p_b \geq p_s, \text{ if } p_s \leq 20;\ p_b < p_s, \text{ if } p_s > 20. \quad \text{(A6)}$$

Specifically, the following is an example of the equilibria: $p_s = c$ for q such that $q \leq 2c$; and $p_s(q) = q$ for q such that $q > 2c$, while p_b = c. Here, c is any integer that is less than or equal to 20. With this equilibrium, the expected payoff for the seller is:

$$\pi_s = \int_0^{2c} (c - q/2) \cdot \frac{1}{40} dq = \frac{1}{40} \left[cq - \frac{1}{4} q^2 \right] \Big|_{q=0}^{2c} = \frac{c^2}{40} \; (> 0),$$

and the expected payoff for the buyer is:

$$\int_0^{2c} (q - c) \cdot \frac{1}{40} dq = \left(\frac{1}{2} q^2 - cq \right) \cdot \frac{1}{40} \Big|_0^{2c} = 0.$$

There is no profitable deviation, not only for the seller but also for the buyer. There are many equilibria of this kind.

There is also another kind of equilibrium in which the transaction is not exerted. The following is an example: the seller posts a price that is greater than or equal to 20 always, and the buyer sets her purchase threshold at 0.

A.4. *Inequality-Averse Preferences and Best Response Strategies for the N-IC and R-IC Treatments*

The best response of the seller is the same as that discussed in Section A.2.

For the best response of the buyer, suppose that the buyer is also inequality-averse like the seller: $u_{bi}(\pi_{bi}, \pi_{sj}) = \pi_{bi} - \mu_i \cdot \left(\pi_{sj} - \pi_{bi}\right)^2$. Then, the buyer's best response strategy is derived by maximizing her expected utility given the seller's strategy $p_{sj} = p_s(q)$:

$$E_q\left[u_{bi}(\pi_{bi}, \pi_{sj})\right] = E_q\left[\left\{\pi_{bi} - \mu_i \cdot \left(\pi_{sj} - \pi_{bi}\right)^2\right\} \cdot 1_{\{p_s(q) \leq p_{bi}\}}\right]. \qquad \text{(A7)}$$

That is,

$$p_b(\mu_i) = argmax_x . E_q\left[\left\{\pi_{bi} - \mu_i \cdot \left(\pi_{sj} - \pi_{bi}\right)^2\right\} \cdot 1_{\{p_s(q) \leq x\}}\right]. \qquad \text{(A8)}$$

Here, suppose that $p_s(q)$ is non-decreasing in q. Then (A8) reduces to the following:

$$E_q\left[u_{bi}(\pi_{bi}, \pi_{sj})\right] = \int_0^{p_s^{-1}(p_{bi})} \left[q - p_s(q) - \mu_i \cdot \left(2p_s(q) - \frac{3}{2} q\right)^2\right] \cdot \frac{1}{40} dq. \qquad \text{(A9)}$$

Here, $p_s^{-1}(p_{bi})$ is the upper bound if it has multiple values (correspondence). Since the condition of non-negative utility must be met, we have:

$$\int_0^{p_s^{-1}(p_{bi})} \left[q - p_s(q) - \mu_i \cdot \left(2p_s(q) - \frac{3}{2} q\right)^2\right] dq \geq 0. \qquad \text{(A10)}$$

Although there are multiple equilibria, there is a common feature in that the buyer obtains a positive material payoff in expectation. This is because condition (A10) implies that:

$$\int_0^{p_s^{-1}(p_{bi})} [q - p_s(q)] \; dq \geq \int_0^{p_s^{-1}(p_{bi})} \mu_i \cdot [2p_s(q) - \tfrac{3}{2}q]^2 dq > 0.$$

The BNE is characterized by (A2) (or A3), (A8) and (A10).

From Figure A.3, we have the following features of the equilibria:

(1) The higher the buyer's equilibrium purchase threshold p_{bi}^*, the higher the acceptance rate.

(2) Regardless of which purchase threshold is realized in equilibrium, the seller's equilibrium price is increasing in q and less than p_{bi}^* in a region where $q \leq \frac{4}{3}p_{bi}^* - \frac{1}{6\mu_j}$.

(3) No trades are closed in the region where $q > q^*$.

A.5. *Disapproval Aversion and the Transactions between the Seller and the Buyer in the R-C Treatment*

In this analysis, we assume that the seller's payoff function is expressed as in Eq. (4) of the paper (i.e., $\pi'_{sj} = \pi_{sj} + c \cdot (r - 5)$). Then, the utility of seller j is expressed as:

$$u_{sj}(\pi_{bi}, \pi'_{sj}) = \left\{p_{sj} - \tfrac{1}{2}q + c \cdot (r - 5) - \mu_j \cdot \left(2p_{sj} - \tfrac{3}{2}q + c \cdot (r - 5)\right)^2\right\} \cdot 1_{\{p_{sj} \leq p_{bi}\}}$$

$$+ \left\{c \cdot (r - 5) - \mu_j \cdot \left(c \cdot (r - 5)\right)^2\right\} \cdot 1_{\{p_{sj} > p_{bi}\}}.$$

Likewise, the utility of buyer i is expressed as:

$$u_{bi}(\pi_{bi}, \pi'_{sj}) = \left\{q - p_{sj} - \mu_i \cdot \left(2p_{sj} - \tfrac{3}{2}q + c \cdot (r - 5)\right)^2\right\} \cdot 1_{\{p_{sj} \leq p_{bi}\}}$$

$$+ \left\{-\mu_i \cdot \left(c \cdot (r - 5)\right)^2\right\} \cdot 1_{\{p_{sj} > p_{bi}\}}.$$

We can solve this situation from the second stage (the rating stage) [backward induction].

The second stage (rating stage):

If $p_{sj} > p_{bi}$, buyer i receives a material payoff of 0 points. In the rating stage, the buyer minimizes the term: $\mu_i \cdot \left(c \cdot (r - 5)\right)^2$. This means that the buyer's best response rating score is: $r^* = 5$.

If $p_{sj} \le p_{bi}$, the buyer tries to minimize the term: $\mu_j \cdot \left(2p_{sj} - \frac{3}{2}q + c \cdot (r - 5)\right)^2$ in the rating stage. From $2p_{sj} - \frac{3}{2}q + c \cdot \left(r - 5\right) = 0$, we find:

$$r = 5 + \frac{3}{2c}q - \frac{2}{c}p_{sj}.$$

This is the condition of interior solutions. The negative slope (– 2/c) shows a negative correlation between r and p_{sj}. The buyer's best response correspondence differs by q because $0 \le r \le 10$. We have the following three cases, considering the size of intercept $(5 + \frac{3}{2c}q)$:

(i) If p_{sj} is small enough that $p_{sj} \le -\frac{5}{2}c + \frac{3}{4}q$, $r^* = 10$.

(ii) If p_{sj} is high enough that $p_{sj} > \frac{5}{2}c + \frac{3}{4}q$, $r^* = 0$.

(ii) If $p_{sj} \in [-\frac{5}{2}c + \frac{3}{4}q, \frac{5}{2}c + \frac{3}{4}q]$, $r^* = 5 + \frac{3}{2c}q - \frac{2}{c}p_{sj}$.

[When $q \ge \frac{10c}{3}$:]

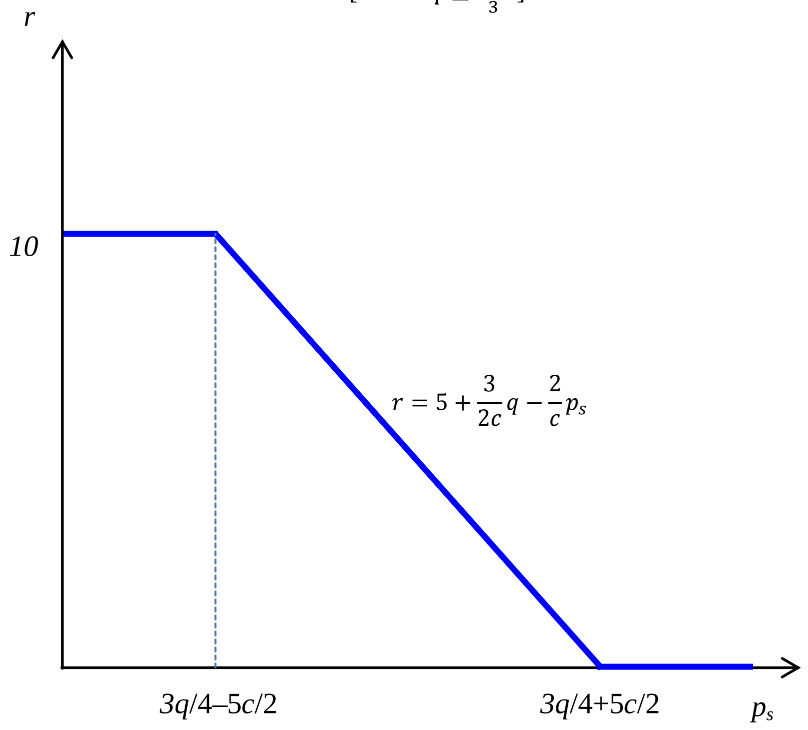

[When $q < \frac{10c}{3}$:]

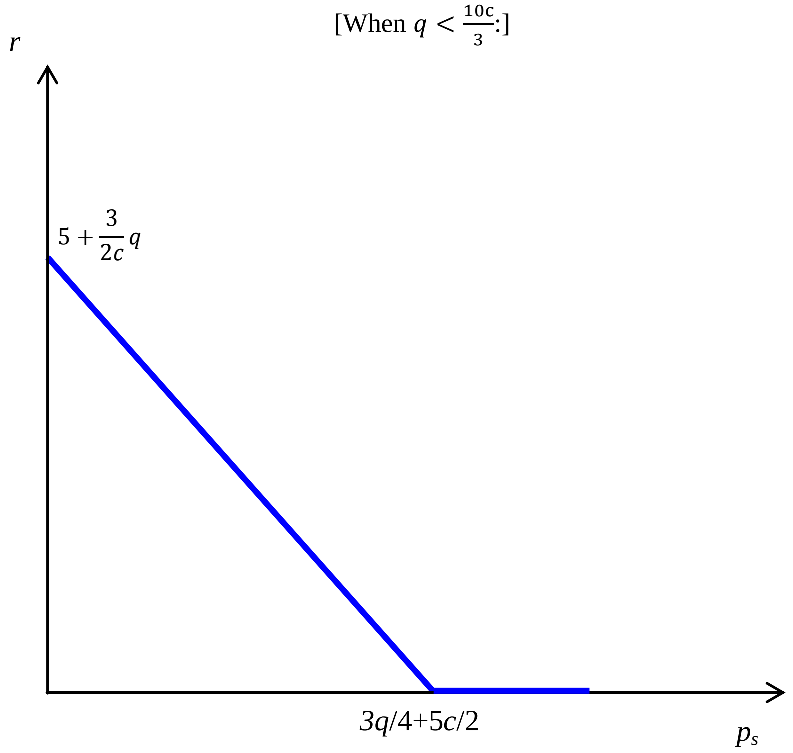

The optimality condition suggests a negative correlation between r and p_{sj}.

In the second stage, there are no real decisions to make for the sellers.

The first stage (the transaction between the seller and buyer):

The buyer's best response can be quickly derived. Given p_{sj}, if $q - p_{sj} - \mu_j \cdot \left(2p_{sj} - \frac{3}{2}q + c \cdot (r^* - 5)\right)^2 \geq 0$, she submits a purchase threshold that is higher than or equal to p_{sj}. Here, r^* is the buyer's best response strategy for rating in the following rating stage. It is clear that because of the rating opportunities, the inequality-averse term in her utility function is smaller in the R-C treatment than in the N-C treatment. Therefore, materially unequal offers by the seller are more likely to be accepted by the buyer. This is consistent with the idea that the buyer substitutes expressing emotions for rejecting offers.

As for the seller, we first consider the interior solution case in the rating stage (see the above). In this case, the seller's utility is: $\left\{p_{sj} - \frac{1}{2}q + c \cdot (r^* - 5)\right\} \cdot 1_{\{p_{sj} \leq p_{bi}\}} = \{q - p_{sj}\} \cdot$

$1_{\{p_{sj} \leq p_{bi}\}}$ whereas the buyers' utility is: $\{q - p_{sj}\} \cdot 1_{\{p_{sj} \leq p_{bi}\}}$. The seller chooses his possible minimum price to offer. The buyer's best response strategy is to submit p_{bi} so that $p_{sj} \leq p_{bi}$ whenever $p_{sj} \leq q$ (her payoff is positive). Thus, in this case, the unique equilibrium is: $p_{sj} = -\frac{5}{2}c + \frac{3}{4}q$ and $p_{bi} = x$ such that $x \geq -\frac{5}{2}c + \frac{3}{4}q$. Thus, we see that the seller chooses to offer lower prices in the R-C treatment in order to avoid receiving disapproval points or to enjoy positive psychological gains.

When r^* = 10 or 0 (corner solution), from $\partial \pi_{sj}/\partial p_{sj} = 0$, we have:

Case 1: $p_{sj} = \frac{1}{8\mu} + \frac{3}{4}q - \frac{5c}{2}$ when r^* = 10, if the seller's utility is then non-negative.

Case 2: $p_{sj} = \frac{1}{8\mu} + \frac{3}{4}q + \frac{5c}{2}$ when r^* = 0, if the seller's utility is then non-negative.

Case 1 is not a solution because the buyer sets r^* = 10 if $p_{sj} < \frac{3}{4}q - \frac{5c}{2}$. In contrast, Case 2 holds as a corner solution because $p_{sj} > \frac{5c}{2} + \frac{3}{4}q$ (see Case II in the analysis of the second stage). The buyer submits p_{bi} so that $p_{sj} \leq p_{bi}$ as long as the buyer's utility is positive. Thus, in the corner solution, the seller offers a materially less fair amount (higher price) to the buyer and the buyer selects r^* = 0 in the second stage.

A.6. *Inequality Aversion, Disappointment Aversion, and Players' Best Responses in N-IC and R-IC treatments*

In this subsection, we study how theoretical predictions may change if inequality-averse actors also exhibit disappointment aversion due to asymmetric information on q in the incomplete information treatments. For simplicity, we assume that sellers do not exhibit social disapproval aversion in this analysis. That is, we assume that buyers' rating behaviors will not affect the utilities of sellers. We note that calculations become messy if we have both disapproval aversion and disappointment aversion in the model. However, even if we incorporate both disapproval aversion and disappointment aversion into the modeling of subjects' inequality-averse preference (Eqs. (1) to (3)) with the assumption that buyers can cancel out negative emotions from disappointment by releasing the emotions, we obtain the same implications (the degree of inequity acceptance is stronger in the incomplete information than in the complete information settings because of disappointment aversion).[3] In this Appendix, we show a simpler version of the analysis for an illustrative purpose.

We assume that with incomplete information (when q is unknown to buyers) buyers select p_b based on the expected value of q. In a model with disappointment aversion (e.g., Bonomo, Garcia, Meddahi, and Tédongap 2010, Gul 1991, Routledge and Zin 2010), buyer *i* will incur a psychological disutility when the realized q_t was *less than* her expectation (i.e., $E(q) = 20$). The absolute value of i's psychological loss is assumed to be increasing in p_b. We will incorporate the model of disappointment aversion into our model with inequality aversion (Eqs. (1) to (3) in the paper) as follows. First, we write the payoff function for buyer *i* in the N-IC treatment as below:

$$\pi_b^N = \{(q_t - p_s) + p_b \alpha(\mathrm{q} - 20) \cdot 1_{\{q<20\}}\} \cdot 1_{\{p_s \leq p_b\}},$$

where $1 > \alpha > 0$.

We further assume that buyers' loss from disappointment would be 0 once it is released (see psychological papers for this argument, such as Xiao and Houser [2005], Campbell-Sills *et al.* [2006], and Gross and John [2003]). This means that buyers strategically utilize the rating opportunity to deal with their negative emotions from disappointment (realized low q_t). In other words, buyers do not utilize rating opportunities to verbally punish or reward the behavior of their

[3] We need to consider both interior solution and corner solution cases as in Section A.5 if we have both disapproval and disappointment aversion in an analysis.

matched sellers. Based on this assumption, we can write the payoff function for buyer *i* in the R-IC treatment as below.

$$\pi_b^R = (q_t - p_s) \cdot 1_{\{p_s \le p_b\}}.$$

Second, we calculate the utilities of buyer *i* and seller *j* in the incomplete information treatments based on Eqs. (1) to (3):

$$u_b^R = \{\pi_b - \mu_i(\pi_{sj} - \pi_{bi})^2\}.$$

$$u_b^N = \{\pi_b^N - \mu_i(\pi_{sj} - \pi_{bi}^N)^2\}.$$

$$u_s^R = \left\{\pi_s - \mu_j(\pi_{sj} - \pi_b)^2\right\}.$$

$$u_s^N = \left\{\pi_s - \mu_j(\pi_{sj} - \pi_b^N)^2\right\}.$$

Here, the superscripts *N* and *R* refer to treatments without and with rating opportunities, respectively.

In Section A.6, we denote $p_b^N = arg\ max\ E(u_b^N)$;

$p_b^R = arg\ max\ E(u_b^R)$;

$p_s^N = arg\ max\ u_s^N$;

$p_s^R = arg\ max\ u_s^R$.

Proposition 1. $p_b^N \le p_b^R$.

<u>Proof</u>.

Let $r^-(q) = \alpha(\mathrm{q} - 20) \cdot 1_{\{q<20\}}$.

We can rewrite π_b^N as:

$$\pi_b^N = \pi_b^R + r^- p_b 1_{\{p_s \le p_b\}}.$$

Thus we have:

$$E(u_b^N) = \frac{1}{40}\int_0^{40} \pi_b^N - \mu(\pi_s - \pi_b^N)^2$$

$$= \frac{1}{40}\int_0^{40} \pi_b^R + r^- p_b 1_{\{p_s \le p_b\}} - \mu(\pi_s - \pi_b^R)^2 + 2\mu(\pi_s - \pi_b^R) r^- p_b - \mu(r^-)^2 p_b^2 1_{\{p_s \le p_b\}}$$

$$= E(u_b^R) + \frac{1}{40}\int_0^{40} r^- p_b 1_{\{p_s \le p_b\}} + 2\mu(\pi_s - \pi_b^R) r^- p_b - \mu (r^-)^2\, p_b^2 1_{\{p_s \le p_b\}}$$

$$= E(u_b^R) + \frac{1}{40}\int_0^{40} r^- p_b 1_{\{p_s \le p_b\}}$$

$$+ \frac{1}{40}\int_0^{40} 2\mu(\pi_s - \pi_b^R) r^- p_b 1_{\{p_s \le p_b\}}$$

$$- \frac{1}{40}\int_0^{40} \mu(r^-)^2 p_b^2 1_{\{p_s \le p_b\}}.$$

Here, we call:

$$\frac{1}{40}\int_0^{40} r^- p_b 1_{\{p_s \le p_b\}} = A(p_b),$$

$$\frac{1}{40}\int_0^{40} 2\mu(\pi_s - \pi_b^R) r^- p_b 1_{\{p_s \le p_b\}} = \mathrm{B}(p_b),$$

$$- \frac{1}{40}\int_0^{40} \mu(r^-)^2 p_b^2 1_{\{p_s \le p_b\}} = C(p_b).$$

We can show that A, B, C are non-positive decreasing functions.

First, because $(\pi_s - \pi_b) \ge 0$, $r^- \le 0$, A and B are non-positive, decreasing functions, respectively. Second, $(r^-)^2 \ge 0$ implies that C is also a non-positive decreasing function.

Now suppose that $\mathrm{y} > p_b^R$. Then,

$$E(u_b^N)(y) = E(u_b^R)(y) + A(y) + B(y) + C(y)$$

$$< E(u_b^R)(p_b^R) + A(p_b^R) + B(p_b^R) + C(p_b^R) = E(u_b^N)(p_b^R).$$

This means that $y \ne argmax(E(u_b^N))$, which implies $p_b^N = argmax(E(u_b^N)) \le p_b^R$.

Proposition 2. When q is small enough, $p_s^N < p_s^R$

Sellers' best responses in the R-IC treatment is (A2). By using the same calculation process as in (A2), we can find sellers' best response function in the N-IC treatment.

$$p_s^N = \begin{cases} \frac{1}{8\mu_j} + \frac{3}{4}q + \frac{f(q)}{2}p_b, & \text{if } \frac{1}{8\mu_j} + \frac{3}{4}q + \frac{f(q)}{2}p_b \le p_b \\ p_b, & \text{if } \frac{1}{8\mu_j} + \frac{3}{4}q + \frac{f(q)}{2}p_b > p_b \text{ and } \pi_s - \mu_j(\pi_s - \pi_b^N)^2 > 0 \\ \text{any } c > p_b, & \text{if } \frac{1}{8\mu_j} + \frac{3}{4}q + \frac{f(q)}{2}p_b > p_b \text{ and } \pi_s - \mu_j(\pi_s - \pi_b^N)^2 \le 0 \\ & \text{where } f(q) = \alpha(q-20)1_{\{q<20\}} \end{cases}$$

REFERENCES

Bonomo, M,, R.Garcia, N. Meddahi, and R. Tédongap. (2010). Generalized disappointment aversion, long-run volatility risk, and asset prices. *Experimental Economics*, 24(1), 82-122.

Campbell-Sills, L., D. H. Barlow, T. A. Brown, and S. G. Hofmann. (2006). Effects of suppression and acceptance on emotional responses of individuals with anxiety and mood disorders. *Behaviour Research and Therapy*, 44(9), 1251-1263.

Gul, F. (1991). A theory of disappointment aversion. *Econometrica*, 59(3), 667-686.

Gross, J. J., and O. P. John. (2003). Individual differences in two emotion regulation processes: Implications for affect, relationships, and well-being. *Journal of Personality and Social Psychology*, 85(2), 348-362.

Routledge, B. R., and S. E. Zin. (2010). Generalized disappointment aversion and asset prices. *Journal of Finance*, 65(4), 1303-1332.

Xiao, E., and D. Houser. (2005). Emotion expression in human punishment behavior. *Proceedings of the National Academy of Sciences of the United States of America*, 102(20), 7398-7401.

Appendix B: Additional Tables

TABLE B.1:

The Determinants of the Acceptance Rates of Offers

Dependent variable: Dummy that equals 1 if a transaction between buyer *i* and seller *j* was closed in period *t*

Independent variables	(1)	(2)
(a) Value of the commodity in period *t* (q_t) {= 0, 1, …, 39, 40}	-0.022***<br>(0.002)	-0.023***<br>(0.004)
(b) Rating dummy {which equals 1 for the R-C or R-IC treatment; 0 otherwise}	0.053<br>(0.052)	0.003<br>(0.133)
(c) Complete information dummy {which equals 1 for the N-C and R-C treatments; 0 otherwise}	0.419***<br>(0.069)	0.482***<br>(0.074)
(d) Period = {1, 2, …, 50}	0.008***<br>(0.001)	0.009***<br>(0.002)
(a) × (b)	---	0.002<br>(0.005)
(b) × (d)	---	0.0002<br>(0.003)
(c) × (d)	---	-0.003<br>(0.002)
Constant	0.611***<br>(0.141)	0.609***<br>(0.156)
# of observations	8000	8000
Log likelihood	-4483.90	-4482.83
Wald chi^2	249.37	221.32
Prob > chi^2	< 0.0001	< 0.0001

Notes: Random-effects probit regressions with bootstrap standard errors (the number of replications is 200). Numbers in parenthesis are robust standard errors. Demographic variables of buyers and sellers are included to control for individual characteristics. Control variables include a USA dummy (=1 if sessions were conducted in the USA; 0 otherwise), a female dummy (=1 if female; and 0 otherwise), number of economics courses taken, general political orientation (1 = very conservative to 7 = very liberal) and income of the subject's family. We omitted the coefficient estimates of these demographic variables to conserve space since these are not related to the hypotheses in the paper.
*, **, and *** indicate significance at the 0.10 level, at the 0.05 level and at the 0.01 level, respectively.

TABLE B.2:

The Determinants of the Rating Decisions by Buyers (Supplementing Table 2 of the paper)

Dependent variable: Rating that buyer *i* gave to the matched seller *j* in period $t \in \{1, 2, ..., 50\}$

The following are the estimation results with the Heckman's two-stage selection model.

[Second Stage Regression]

Independent variables	When deals were closed (1)	When deals were not closed (2)	When deals were closed (3)	When deals were not closed (4)
(a) Seller's keep in period *t* (i.e., $p_{sj,t} - q_t/2$)	-0.594*** (0.014)	-0.388*** (0.022)	----	----
(b) $\frac{\text{Seller's keep in period } t}{q_t/2}$	----	----	-0.324*** (0.016)	-0.235*** (0.026)
(c) Complete information dummy {which equals 1 for the N-C and R-C treatments; 0 otherwise}	-1.448*** (0.202)	-5.704*** (0.408)	3.268*** (0.343)	-1.603 (1.064)
(d) Interaction term between variable (a) and variable (c)	0.484*** (0.025)	0.376*** (0.034)	----	----
(e) Interaction term between variable (b) and variable (d)	----	----	-2.579*** (0.521)	-1.359 (1.413)
Period Number (= {1, 2, …, 50})	-0.001 (0.003)	-0.022*** (0.006)	-0.007* (0.004)	-0.018** (0.007)
Constant	9.292*** (0.318)	7.112*** (0.570)	5.697*** (0.344)	4.661*** (0.504)
# of observations	3,920	3,920	3,720	3,720
Censored observations	1072	2848	1019	2701
Wald chi^2	2358.52	683.52	921.1	364.96
Prob > chi^2	< 0.0001	< 0.0001	< 0.0001	< 0.0001
Two-sided *p*-value for the null:				
H_0: variable (a) = 0	< 0.001	< 0.0001	----	----
H_0: variable (b) = 0	----	----	< 0.0001	< 0.0001
H_0: variable (a) + variable (d) = 0	< 0.001	0.6909	----	----
H_0: variable (b) + variable (e) = 0	----	----	< 0.0001	0.2567

Notes: Numbers in parenthesis are robust standard errors. Control variables include buyers' demographic variables: a USA dummy (=1 if sessions were conducted in the USA; 0 otherwise), a female dummy (=1 if female; 0 otherwise), number of economics courses taken, general political orientation (1 = very conservative to 7 = very liberal) and income of the subject's family. We omitted the coefficient estimates of these demographic variables to conserve space as these are not related to the hypotheses in the paper.

*, **, and *** indicate significance at the 0.10 level, at the 0.05 level and at the 0.01 level, respectively.

[First Stage: Selection Equation]

Please note that the absolute values of coefficient estimates in column (1') are identical to those in column (2') with the sign being opposite. The same holds for columns (3') and (4').

Equations (1'), (2'), (3') and (4') in the table are the selection equations of columns (1), (2), (3),and (4), respectively, on the previous page.

Independent variables	When deals were closed (1')	When deals were not closed (2')	When deals were closed (3')	When deals were not closed (4')
Buyers' last period purchase threshold (i.e., p_{bi}^{t-1}) [instrument]	0.032*** (0.003)	-0.032*** (0.003)	0.035*** (0.003)	-0.035*** (0.003)
(a) Seller's keep in period t (i.e., $p_{sj,t} - q_t/2$)	-0.020*** (0.006)	0.020*** (0.006)	----	----
(b) $\frac{\text{Seller's keep in period } t}{q_t/2}$	----	----	0.025*** (0.007)	-0.025*** (0.007)
(c) Complete information dummy {which equals 1 for the N-C and R-C treatments; 0 otherwise}	0.891*** (0.089)	-0.891*** (0.089)	3.219*** (0.160)	-3.219*** (0.160)
(d) Interaction term between variable (a) and variable (c)	-0.062*** (0.011)	0.062*** (0.011)	----	----
(e) Interaction term between variable (b) and variable (d)	----	----	-4.392*** (0.251)	4.392*** (0.251)
Period number (= {1, 2, …, 50})	0.006*** (0.002)	-0.006*** (0.002)	0.007*** (0.002)	-0.007*** (0.002)
Constant	-0.328*** (0.116)	0.328*** (0.116)	-0.691*** (0.115)	0.691*** (0.115)

Notes: Numbers in parenthesis are robust standard errors. Control variables include buyers' demographic variables: a USA dummy (=1 if sessions were conducted in the USA; 0 otherwise), a female dummy (=1 if female; 0 otherwise), numbers of economics courses taken, general political orientation (1 = very conservative to 7 = very liberal) and income of the subject's family. We omitted the coefficient estimates of these demographic variables to conserve space as these are not related to the hypotheses in the paper.

*, **, and *** indicate significance at the 0.10 level, at the 0.05 level and at the 0.01 level, respectively.
